# Natural history of disease in cynomolgus monkeys exposed to Ebola virus Kikwit strain demonstrates the reliability of this non-human primate model for Ebola virus disease

**DOI:** 10.1371/journal.pone.0252874

**Published:** 2021-07-02

**Authors:** Nancy A. Niemuth, Dawn Fallacara, Cheryl A. Triplett, Sanjay M. Tamrakar, Alisha Rajbhandari, Clint Florence, Lucy Ward, Anthony Griffiths, Ricardo Carrion, Yenny Goez-Gazi, Kendra J. Alfson, Hilary M. Staples, Trevor Brasel, Jason E. Comer, Shane Massey, Jeanon Smith, Andrew Kocsis, Jake Lowry, Sara C. Johnston, Aysegul Nalca, Arthur J. Goff, Amy C. Shurtleff, Margaret L. Pitt, John Trefry, Michael P. Fay

**Affiliations:** 1 Battelle, Columbus, OH, United States of America; 2 Non-Clinical Advisor for Joint Program Manager Chemical, Biological, Radiological and Nuclear Medical (JPM CBRN Medical) Joint Program Executive Office for Chemical, Biological, Radiological and Nuclear Defense (JPEO CBRND), Fort Detrick, MD, United States of America; 3 Advanced Vaccines & Immunologics, JPM CBRN Medical, JPEO CBRND, Fort Detrick, MD, United States of America; 4 Department of Microbiology, National Emerging Infectious Diseases Laboratories, Boston University, Boston, MA, United States of America; 5 Texas Biomedical Research Institute, San Antonio, TX, United States of America; 6 Department of Microbiology and Immunology/Institutional Office of Regulated Nonclinical Studies, University of Texas Medical Branch (UTMB), Galveston, TX, United States of America; 7 Institutional Office of Regulated Nonclinical Studies, UTMB, Galveston, TX, United States of America; 8 Animal Resources Center, UTMB. Galveston, TX, United States of America; 9 Virology Division, United States Army Medical Research Institute of Infectious Diseases (USAMRIID), Fort Detrick, MD, United States of America; 10 Core Support Directorate, USAMRIID, Fort Detrick, MD, United States of America; 11 Commander’s Special Staff, Deputy Director Research Program Office, USAMRIID, Fort Detrick, MD, United States of America; 12 Molecular Biology Division, USAMRIID, Fort Detrick, MD, United States of America; 13 USAMRIID, Fort Detrick, MD, United States of America; 14 Vaccines/Therapeutics Division (CBM), Defense Threat Reduction Agency, Fort Belvoir, Virginia, United States of America; 15 National Institute of Allergy and Infectious Diseases, National Institutes of Health, Bethesda, MD, United States of America; CEA, FRANCE

## Abstract

Filoviruses (Family *Filoviridae* genera *Ebolavirus* and *Marburgvirus*) are negative-stranded RNA viruses that cause severe health effects in humans and non-human primates, including death. Except in outbreak settings, vaccines and other medical countermeasures against Ebola virus (EBOV) will require testing under the FDA Animal Rule. Multiple vaccine candidates have been evaluated using cynomolgus monkeys (CM) exposed to EBOV Kikwit strain. To the best of our knowledge, however, animal model development data supporting the use of CM in vaccine research have not been submitted to the FDA. This study describes a large CM database (122 CM, 62 female and 60 male, age 2 to 9 years) and demonstrates the consistency of the CM model through time to death models and descriptive statistics. CMs were exposed to EBOV doses of 0.1 to 100,000 PFU in 33 studies conducted at three Animal Biosafety Level 4 facilities, by three exposure routes. Time to death was modeled using Cox proportional hazards models with a frailty term that incorporated study-to-study variability. Despite significant differences attributed to exposure variables, all CMs exposed to the 100 to 1,000 pfu doses commonly used in vaccine studies died or met euthanasia criteria within 21 days of exposure, median 7 days, 93% between 5 and 12 days of exposure. Moderate clinical signs were observed 4 to 5 days after exposure and preceded death or euthanasia by approximately one day. Viremia was detected within a few days of infection. Hematology indices were indicative of viremia and the propensity for hemorrhage with progression of Ebola viremia. Changes associated with coagulation parameters and platelets were consistent with coagulation disruption. Changes in leukocyte profiles were indicative of an acute inflammatory response. Increased liver enzymes were observed shortly after exposure. Taken together, these factors suggest that the cynomolgus monkey is a reliable animal model for human disease.

## Introduction

Filoviruses (Family *Filoviridae* genera *Ebolavirus* and *Marburgvirus*) are negative-stranded RNA viruses that are known to infect humans and non-human primates causing severe health consequences, including death. Infection with viruses from the Ebolavirus genus cause Ebola virus disease (EVD), formerly known as Ebola hemorrhagic fever, with death often occurring within 7 to 10 days after onset of symptoms. Past outbreaks have resulted in case fatality rates of 25% to 90% in humans [1]. The 2014–2016 Ebola virus outbreak in West Africa ended in June 2016 with more than 28,600 cases and 11,325 deaths (40% fatality rate) [2]. The recently ended outbreak in North Kivu, Ituri, Democratic Republic of Congo (DRC) had an overall case fatality rate of 66% with 2,299 fatal cases as of July 3, 2020 [3], while an overlapping outbreak that began in June 2020 in Equateur Province, DRC had reached 100 cases by August 2020 with 43 lives lost [4, 5].

The 2014–2016 Ebola virus outbreak in West Africa resulted in the rapid advancement of multiple vaccine and therapeutic candidates from preclinical to clinical testing. Some of these investigational products were tested in the 2018–2020 DRC outbreak [6, 7]. The first of these products was approved by the European Medicines Agency (EMA) and FDA in fall of 2019 [8, 9]. An additional vaccine administered as a two-dose regimen of two components was approved by the EMA in July 2020 [10, 11]. Except in outbreak settings, it is likely that vaccines and other medical countermeasures (MCM) against Ebola virus will require development and testing in accordance with the FDA Animal Rule (AR) [12], or AR-associated concepts, prior to receiving FDA marketing approval. The AR requires that products be tested in well-characterized animal models that are relevant to human disease and that the animal study endpoints be clearly related to the desired benefit in humans, generally the enhancement of survival or prevention of major morbidity.

The cynomolgus monkey (CM, *Macaca fascicularis*) non-human primate (NHP) is the most widely used animal model for Ebola virus vaccine testing [13]. Clinical signs of EVD in CM parallel human disease, but pathogenesis and time to death are more rapid in the CM model. This difference may be due to the high exposure doses (usually 100 to 1000 PFU) used in vaccine research compared to human exposure levels, which are typically unknown but likely lower. Multiple filovirus vaccine candidates have been evaluated using the CM NHP model [13], and it is currently utilized for vaccine studies supporting licensure under the Animal Rule; however, to the best of our knowledge, animal model development data such as natural history studies supporting the use of the CM NHP model have not been submitted to the FDA. The intent of this study is to provide animal model data for the CM NHP model to support MCM licensure under the AR, using historical data to reduce the number of animals required for research, in adherence to the principles of the 3Rs (Replacement, Reduction, and Refinement) of animal research. Because the CM is a highly sentient species, limiting the numbers of CMs used for research is an ethical imperative. This dataset may be used by multiple product developers, thereby reducing the costs for product development and avoiding duplication of research.

This study summarizes a meta-analysis that characterizes the effects of Ebola virus Kikwit strain (EBOV) on CMs using unpublished data provided by study sponsors from a large set of 122 EBOV-exposed, sham-vaccinated control CMs across 33 EBOV vaccine studies, where most studies provided 2 to 4 sham-vaccinated control CMs. The sham-vaccinated control CMs were exposed to EBOV doses ranging from 0.1 to 100,000 PFU, by intramuscular (IM), intranasal (IN), or aerosol exposure routes, in studies conducted at three Animal Biosafety Level 4 (ABSL-4) facilities. The meta-analysis focuses on the post-EBOV exposure phase of the vaccine studies and includes only the sham-vaccinated control CMs.

The nature of the control article varied from saline solutions to a vaccine that harbored an irrelevant antigen. The sham-vaccinated control CMs from these studies were exposed to EBOV but had not been immunized with an EBOV vaccine, and therefore provide valuable information on the progression of EVD in CMs and the consistency of the CM model under varying exposure conditions. The meta-analysis utilizes data from sham-vaccinated control CMs from these 33 studies to describe the pathogenesis and disease progression of EVD in CM in relation to what is currently known about human EVD pathogenesis. Through time to death models and descriptive statistics, we show that the CM model was reliable and consistent despite the variability in animal and exposure characteristics.

In order to maximize the limited capacity of ABSL-4 facilities, EBOV vaccine studies are typically performed in separate immunization and exposure phases, where the immunization phase is conducted in Animal Biosafety Level 2 (ABSL-2) laboratory space, and CMs are transferred to a ABSL-4 laboratory for EBOV exposure. For some studies, the transfer was simply between ABSL-2 and ABSL-4 laboratories at the same facility. In many cases, however, the transfer involved shipping the CMs to a ABSL-4 test facility in a different city or state and acclimation in ABSL-2 housing at the ABSL-4 test facility prior to transfer to the ABSL-4 laboratory. Most CMs were acclimated in the ABSL-4 test facility for 7 to 10 days prior to exposure, even if they originated in the same facility. Possible effects of transport and acclimation have been documented [14] but were not considered in this analysis.

## Materials and methods

### CM animal model

The sham-vaccinated control population consisted of a total of 122 CMs (*Macaca fascicularis*), 62 females and 60 males. Age was available for 96 CMs that ranged from 2 to 9 years of age, with very few younger than 3 years or older than 5 years at the time of first sham vaccination. CMs are considered juveniles through 36 months, females reach sexual maturity at 4 years and males at 7 years [15]. Therefore, CMs aged 2–3 years were considered juveniles and those ages 4 years and older were categorized as adults; the latter includes a mixture of adolescents and adults.

The CMs were categorized as either Mauritian or Asian origin in the meta-analysis. Chinese breeders provide over 65% of CMs imported to the US, and the Chinese colonies generally have wild “founders” from Indonesia, Philippines, and Indochina, including Cambodia, Laos, and Vietnam [16]. Therefore, all CMs identified as Chinese, Asian, Cambodian, or Vietnamese in the study reports were combined as Asian for purposes of the meta-analysis.

The 122 sham-vaccinated control CMs were obtained from 33 EBOV vaccine studies, where most studies provided 2 to 4 sham-vaccinated control CMs. None of the 33 studies were conducted under compliance with Good Laboratory Practice for Nonclinical Laboratory Studies (GLP) regulations. More complete data (i.e., more parameters) were available from some studies than others. In general, data quality was dependent on the quality of the source reports, report appendices, and summary data provided by the sponsors; data entry was verified against the source material. Fourteen of the study reports were final and seven were draft. Summary data were provided by the sponsors for the remaining studies. Most studies provided sham-vaccinated control data from 2 to 4 CMs that were exposed to EBOV but had not been immunized with an EBOV vaccine. Following the last treatment and generally about two weeks prior to exposure, the CMs were moved to the ABSL-4 exposure facility and exposed to EBOV.

### Animal welfare statement

All studies presented in this manuscript were approved by the responsible Texas Biomedical Research Institute Institutional Animal Care and Use Committee (IACUC), U.S. Army Medical Research Institute for Infectious Diseases IACUC, or University of Texas Medical Branch IACUC. This retrospective study did not require the use of additional animals and therefore, did not undergo additional review by an ethics committee. Research was conducted in compliance with the Animal Welfare Act. Experiments involving animals adhered to principles stated in the Guide for the Care and Use of Laboratory Animals from the National Research Council. Studies were performed at institutions which are fully accredited by the American Association for Accreditation of Laboratory Animal Care (AAALAC). Animals were euthanized when moribund or at the end of the study following the American Veterinary Medical Association (AVMA) accepted methods of euthanasia.

### Control articles

Control articles were grouped as either PBS/TBS/saline (phosphate buffered saline, tris-buffered saline, or unspecified saline), vaccine buffer/diluent, or vaccine vector. In some cases, vaccine buffer/diluent included adjuvant; vaccine vector included both empty vaccine vectors and vaccine vectors containing an irrelevant antigen. A total of 42, 7, and 54 CMs were administered PBS/TBS/saline, vaccine buffer/diluent, or vaccine vector, respectively.

### EBOV exposure

The EBOV strain used in all studies was isolated from a 65-year-old female patient during an outbreak that occurred in 1995 in the Democratic Republic of Congo (formerly Zaire). The patient exhibited symptoms of disease, was hospitalized, and died. The isolate is designated Ebola virus H.sapiens-tc/COD/1995/Kikwit-9510621. Three virus stocks, designated I, II and III, were derived from this isolate. Virus stock III was the second passage from this isolate, and virus stocks I and II were passaged from virus stock III. All virus stocks were identified as 7U variants [17–20], the majority of which contain a 7-urdylyl stretch at the at the glycoprotein (GP) editing site 6918 to 6924 that produces soluble GP (sGP). Each virus stock was used at a single ABSL-4 facility, designated as Facilities A, B, and C; therefore, exposure facility and virus stock were considered together. A total of 55, 48, and 19 CM were exposed using stock/facility IA, IIB, and IIIC, respectively (Table 1).

**Table 1:** Summary of exposure characteristics.

Virus	Exposure	Number of CMs Exposed								Total by Route	Total by Virus Stock/Exposure Facility
Stock/Exposure Facility	Route	Target Dose (PFU)									
		0.1	0.5	10	100	120	1,000	10,000	100,000		
IA^a^	IM	10	10		27		4			51	55
	IN					4				4	
IIB^a^	Aerosol				4		8			12	48
	IM				10		12			22	
	IN			2	3		3	3	3	14	
IIIC^b^	Aerosol				2		2			4	19
	IM					2	11			13	
	IN					2				2	
Total		10	10	2	46	8	40	3	3	122	122

a. Virus stock is passage 3 of parent isolate 9510621

b. Virus stock is passage 2 of parent isolate 9510621

Intramuscular (IM), intranasal (IN), and aerosol exposure routes were used to administer the EBOV to CMs (Table 1). IM was the most common route used across studies. Source data included both target doses (target exposure administered to each CM) and estimated doses (based on plaque assay of the exposure material; plaque assay procedures varied between exposure facilities). For IM and IN exposures, a single estimate of the exposure dose was provided for each target exposure dose used in a study. For aerosol exposures, the aerosol systems and sampling methodology varied between exposure facilities; however, each facility measured the volume of air inhaled using real time plethysmography. Thus, inhaled doses were estimated for each aerosol-exposed CM.

A broad range of target exposure doses was administered (0.1 to 100,000 PFU); however, CMs were more commonly exposed to the intermediate target exposure doses of 100 or 1,000 PFU. All CMs exposed to very low target doses (0.1 and 0.5 PFU) were from a single study, as were all CMs exposed to very high target doses (10,000 and 100,000 PFU). Because the target exposure doses spanned 6 orders of magnitude and estimated exposure doses overlapped at the 100 and 1,000 PFU target exposure levels, k-means clustering was used to group the CMs into one of 4 exposure dose groups based on the base 10 logarithm of estimated exposure dose (Table 2); for a small number of animals with no estimated exposure dose, the target dose was used in the cluster analysis. The clusters were generated such that each observation belonged to one of 4 clusters with no overlap. Within-cluster variance was minimized, and the log 10 values were back-transformed into the natural scale after clustering. These exposure dose groups were used for descriptive statistics and plots.

**Table 2:** K-means clustering of estimated exposure dose.

Cluster	Exposure dose	N	Geometric mean
	(PFU)		(PFU)
1	0.1– 0.5	20	0.22
2	25–243	49	96.56
3	320–1650	41	817.88
4	3900–82600	12	10025.16

### Study endpoints

Clinical observations were made a minimum of once daily. In some studies, the frequency of clinical observations increased after clinical signs of illness were observed, up to 6 times daily. Clinical observations varied between exposure facilities but generally included responsiveness, severity and onset of rash, bleeding location and onset of bleeding, respiration, elevated body temperature, food consumption, signs of dehydration, stool, edema, and appearance of hair/coat. These observations were used to assign a clinical score or euthanasia score which was used to assess whether the CM met the criteria for humane termination.

Criteria for moribund euthanasia varied between ABSL-4 facilities. Two facilities used similar two-tiered euthanasia criteria. At these facilities, CMs that were “persistently prostrate, severely or completely unresponsive, may have signs of respiratory distress” met the primary euthanasia criteria. CMs that were “prostrate but able to rise if stimulated or moderate to dramatically reduced to response to external stimuli” met the secondary euthanasia criteria if elevated body temperature or abnormal clinical chemistry were also present. At the third facility, any CM that “lies down; gets up with some prodding but not when approached” met the euthanasia criteria. Moribund CM were euthanized promptly, after facility procedures for veterinary approval and terminal data collection were completed. These procedures take 20 minutes or longer, however, the administration of anesthetic occurs as soon as possible (within 5 minutes) to alleviate further distress.

To evaluate clinical/euthanasia scoring across studies, scores were aligned based on activity levels. A total of six categories were ultimately defined across studies. Clinical signs of EVD infection were categorized normal, mild, or moderate; euthanasia criteria were categorized as secondary or primary; and the final category was used for CMs found dead in cage (FDIC). All animals FDIC died from EVD. When multiple scores were available (i.e., multiple clinical or euthanasia scores on a single day), the maximum daily score was included in the meta-analysis.

Several endpoints were used to evaluate EVD pathogenesis, and parameters and data collection time points varied between studies. Whenever possible, variation in data recording and/or reporting was accounted for through unit conversions (e.g., temperatures were converted to the Celsius scale). Although the timeline for data collection varied between studies, protocol-specified activities generally included scheduled body weight, body temperature, and blood collections (e.g., clinical pathology, viral load measurements) for CMs surviving to post-exposure days (PED) 0, 3, 6, 10, 14, and end of study at PED 21 or 28. Terminal collections were often performed for CMs that met euthanasia criteria; however, the terminal collection was not always clearly identified as such in the source data. For CMs that did not have a clearly identified terminal blood collection, the last post-exposure blood collection was considered “terminal” if it was collected on the day the CM died. No terminal collections were performed for CM that did not meet euthanasia criteria and were FDIC. Given that few CMs remained after PED 10, the analysis is limited to the scheduled and unscheduled collections through day 10, and terminal collections from CMs that met euthanasia criteria. Blood samples were obtained for clinical chemistry, hematology, coagulation and viral load in serum or plasma; however, only IIB stock/facility studies collected tissues for viral load measurements. Endpoints used to evaluate hematology, coagulation, and clinical chemistry varied across studies, and some endpoints were collected at a single facility only. Therefore, endpoints commonly represented across meta-analysis studies, and those considered potential indicators of post-exposure EBOV infection, such as fever, hemorrhage, inflammatory response, or viremia, were included in the analysis. Changes in body weight, body temperature, coagulation parameters, white blood cell (WBC) counts, red blood cell (RBC) counts, clinical chemistry, as well as viral loads in serum and tissue, were used to describe EVD disease progression in the CM animal model post-exposure.

### Statistical methods

For continuous data (body weight, temperature, coagulation, hematology, clinical chemistry, and viral loads), descriptive statistics were generated including the number of CMs, mean and standard deviation (SD) or geometric mean (GM) and coefficient of variation (%CV), 95% confidence interval, minimum, and maximum at each time point. The decision to present the arithmetic vs. geometric mean was based on a Shapiro-Wilk tests applied to the baseline (PED 0) data for each endpoint to determine whether a normal or lognormal distribution provided a better fit to the data. Descriptive statistics were calculated for endpoints collected from at least 30 CMs (~25% of the total CM population of 122) at time points where data were available for at least 3 CMs. The descriptive analysis considered animal and exposure characteristics, but statistical hypothesis tests were not performed due to changes in variability and disparity in group sizes over time; therefore, the evaluation of EVD progression based on these endpoints is entirely descriptive.

Survival and time to death are expected to be the primary efficacy endpoints in AR efficacy studies. In a first stage of analysis for these endpoints, Kaplan-Meier survival curves were plotted together with their pointwise 95% confidence intervals, and they were used to estimate the median survival time [21]. Group level comparisons between variables were conducted for sex, age, animal origin, control article, estimated exposure dose, exposure route, exposure stock/facility, and euthanasia status using log rank tests.

The Kaplan-Meier analysis and log rank tests did not account for variability between studies. Therefore, additional models were fit using Cox proportional hazards (PH) models with a frailty term that explicitly incorporated study-to-study variability [22–24]. The Cox PH-frailty models were developed in several stages. First individual Cox PH-frailty models were fit with sex, age, animal origin, control article, estimated exposure dose, exposure route, exposure stock/facility, and euthanasia status as the predictor variable. Next, a full model was developed using the variables that had significant effects in the individual models. Interactions between estimated exposure dose and the other variables were also considered in the full model. A final stage of modeling added clinical pathology biomarkers of inflammation or coagulation disruption to the full model as time-dependent covariates.

Since the Cox PH-frailty model allows for continuous data, age (years) and estimated exposure dose (PFU) were modeled as continuous covariates, with a log base 10 transformation applied to the estimated exposure dose. Exploratory data analysis showed that exposure dose was partially confounded with exposure facility, as nearly all high dose CMs were exposed at a single exposure facility, while all low dose CMs were from a single study conducted at a different exposure facility. Therefore, a subset analysis was conducted which included only the 90 CMs exposed to 100 to 1,000 PFU. The exposure dose groupings of 25 to 243 PFU and 320 to 1,650 PFU described above were used to select CMs for the subset analysis, but the model was fit to the log of estimated exposure dose for each CM, which was modeled as a continuous covariate.

In the final stage of modeling, the biomarker variables were added individually to the full model as many of the biomarkers are highly correlated. A log base 2 transformation was used in these models.

Cox PH-frailty model parameters were expressed as hazard ratios (HRs), where a HR of 1 is interpreted as no difference in the risk (of death) between groups. A HR<1 indicates that the comparison group has lower risk than the baseline group. A HR>1 indicates the comparison group has higher risk than the baseline group. For continuous parameters (age, dose), the HR is the change in hazard for a unit increase in the parameter value. For log transformed parameters, the HR is the change in hazard for a 10-fold change in dose or a 2-fold change in other endpoints.

## Results

Graphical representations of descriptive statistics are provided to illustrate clinically relevant changes post-EBOV exposure and over time. Tables of descriptive statistics for these endpoints have been provided as Supplemental Data. Endpoints selected for this evaluation are those commonly used to evaluate the progression of EVD in CMs, including clinical signs of illness, body weight and temperature, coagulation, hematology (white blood cells and red blood cells), serum chemistry and viral loads in blood and tissue, and time to death. These data are presented from baseline (pre-exposure; PED 0) through PED 10 and at the terminal collections to show disease progression as it relates to time to onset of clinical symptoms and time to death. Changes from baseline (per endpoint), were often observed beginning on PED 5 (day of first death) or PED 7 (median time to death), and minimum or maximum values were often reached on PED 8 or 9 in the remaining CM. Summary data from both scheduled and unscheduled collections are presented; therefore, there are varying sample sizes over time. Reduced sample size over time is an inherent consequence of continued mortality as EVD infection persists and progresses; therefore, mean ± SD are presented with the sample size (N) at a specific post-exposure day (N*x*, with *x* = PED) for context. Sample sizes at PED 4, 8, 9, and 10 are typically lower than other time points and descriptive statistics at these time points sometimes represent fewer than 10 of the 122 CM.

**Fig 1:**
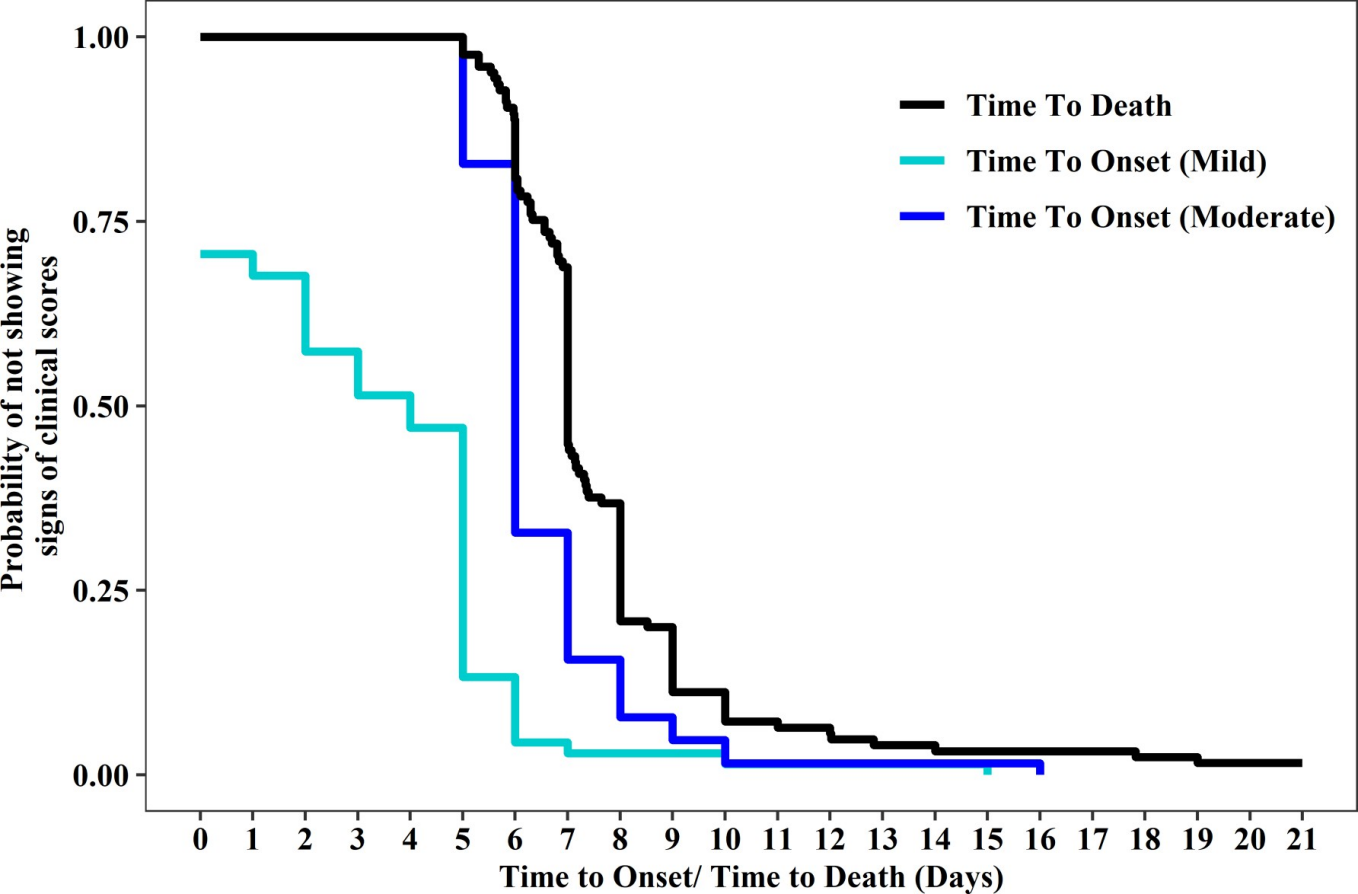
Kaplan-Meier survival probabilities for time to onset of mild and moderate clinical signs and time to death.

Onset times for clinical signs post-EBOV exposure are presented in Fig 1, over all animals and exposure conditions. Mild clinical signs were observed in some CMs at baseline (PED 0) and nearly all by PED 7. Moderate clinical signs were observed beginning on PEDs 4 and 5 and usually preceded death or euthanasia by approximately 1 day. The median time to onset of moderate clinical signs was PED 6 and the median time to death was PED 7. The first death occurred on PED 5 and 93% of deaths occurred between PED 5 and 12. Both CM that survived the EBOV exposure received a very low target dose of 0.1 PFU by the IM route. More CMs met the primary euthanasia criteria than met the secondary euthanasia criteria or were found dead.

**Fig 2:**
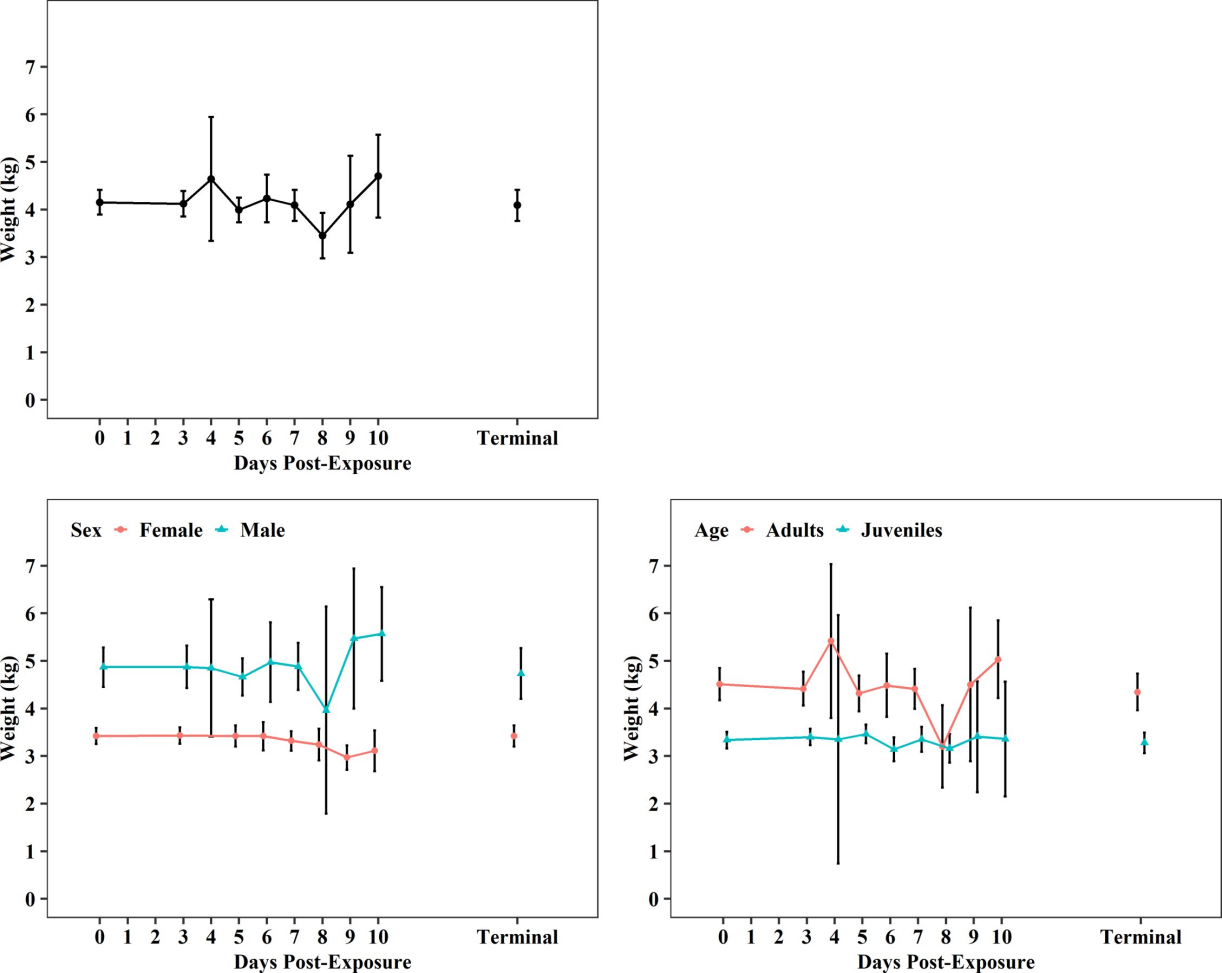
Body weight changes (mean and 95%CI) post-EBOV exposure. Plots show data from all meta-analysis studies as well as differences between sexes and ages.

Body weights were consistent over time from baseline (4.15 ± 1.36 kg, N_0_ = 105) through terminal collections (4.09 ± 1.37 kg, N_T_ = 71), and expected variation was observed between sexes (males > females) and ages (adults > juveniles) of CMs (Fig 2, S1–S3 Tables).

**Fig 3:**
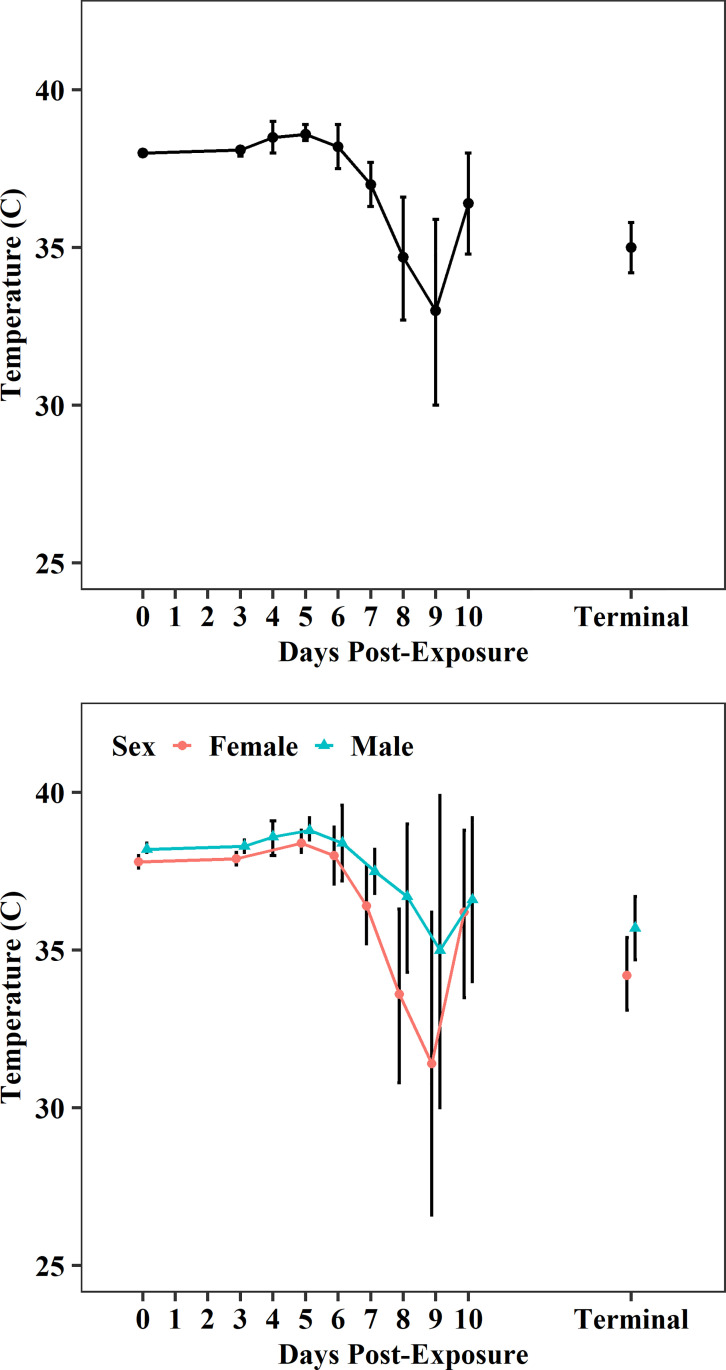
Body temperature changes (mean and 95%CI) post-EBOV exposure. Plots show data from all meta-analysis studies as well as differences between sexes.

Pre-exposure body temperatures (38.0 ± 0.6°C, N_0_ = 105; Fig 3, S4 Table) were within normal ranges reported for CMs (36–38°C) [25]. On average, females exhibited slightly lower body temperatures compared to males (Fig 3, S5 Table). Post-EBOV exposure, mean body temperature changes were not clinically suggestive of fever, although elevated body temperatures were observed in some CMs. On PED 4 and 5, mean temperatures had increased only slightly to 38.5 ± 0.6°C (N_4_ = 8) and 38.6 ± 1.0°C (N_5_ = 63; Fig 3, S4 Table). On PED 5, the variability in the temperature measurements also increased slightly; this was the first day that non-survivors met the euthanasia criteria. Variability in the temperature measurements further increased on PED 6 as EBOV infection progressed and additional non-survivors were euthanized. On PED 7 (the median time to death), body temperatures were highly variable, ranging between 27.4 and 40.4°C (N_7_ = 55; S4 Table). From PED 8 through the terminal collection, body temperatures continued to vary considerably but means were generally lower than baseline (<38°C) and/or lower than normal temperatures reported for CM’s (<36°C), with mean temperatures ranging between 33.0 ± 3.8°C (N_9_ = 9) and 35.0 ± 3.2°C (N_T_ = 68; Fig 3, S4 Table). Terminal body temperatures collected at the time of euthanasia (PED 5–12) may best describe the relationship between alterations in body temperature and EBOV disease progression in CMs; terminal body temperatures ranged between 27.3 and 40.8°C (n = 68), with a 95% CI of (34.2, 35.8), well below baseline values (Fig 3, S4 Table).

**Fig 4:**
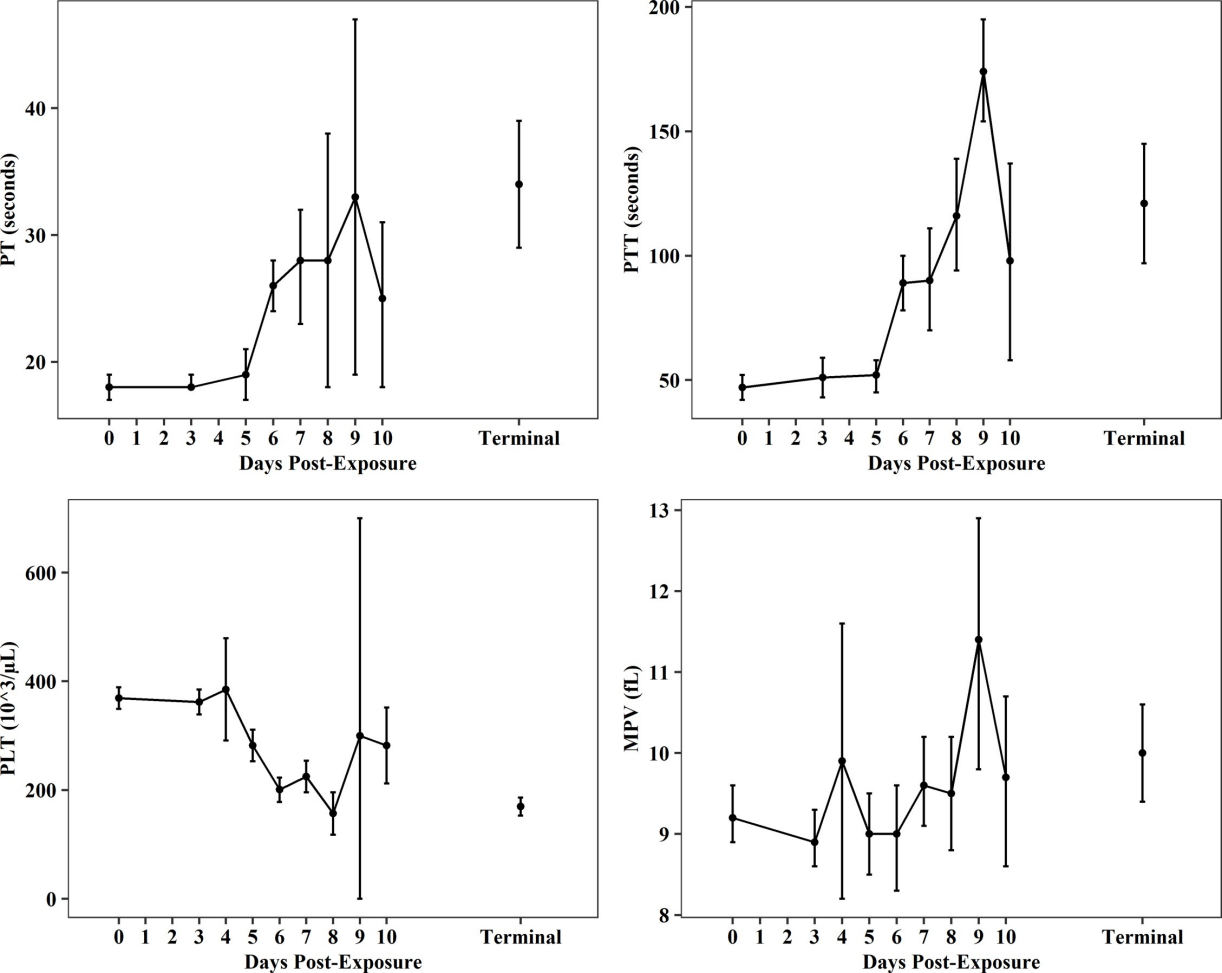
Coagulation and platelet parameters post-EBOV exposure. Plots show data (mean and 95%CI) from all meta-analysis studies for prothrombin time (PT), partial thromboplastin time (PTT), platelets (PLT), and mean platelet volume (MPV).

Changes associated with coagulation parameters and platelets were consistent with coagulation disruption (Fig 4, S6–S9 Tables). Variability in the data increased over time as sample size at scheduled collections decreased with continued mortality post-exposure. Baseline values for mean prothrombin time (PT) and mean partial thromboplastin time (PTT) were higher than normal ranges reported for CMs (PT: 9.7–9.8 sec; PTT: 19.1–19.3 sec) [26]; however, changes from baseline to terminal collections increased 2-3-fold. Mean PT increased from 18 ± 2 (N_0_ = 42) to 34 ±13 (N_T_ = 27) seconds (sec), and PTT increased from 47 ± 19 (N_0_ = 62) to 121 ± 71 sec (N_T_ = 37; Fig 4, S6 and S7 Tables). Changes in platelet concentrations suggest hemorrhage occurred shortly after exposure. From baseline through terminal collections, platelet counts (PLT) decreased from 369 ± 103 (N_0_ = 102) to 170 ± 67 10^3/μL (N_T_ = 68; Fig 4, S8 Table) and were ~40–60% lower than normal values reported for CMs (438–459.1 10^3/μL) [26]. Mean platelet volume (MPV) increased approximately 20% from baseline (9.2 ±1.8 fL, N_0_ = 106) to PED 9 (11.4 ± 2 fL, N_9_ = 9) and terminal collections (10.0 ± 2.6 fL, N_T_ = 69; Fig 4, S9 Table). Terminal MPV was also increased when compared to reference values (8.7–9.3 fL) [26].

**Fig 5:**
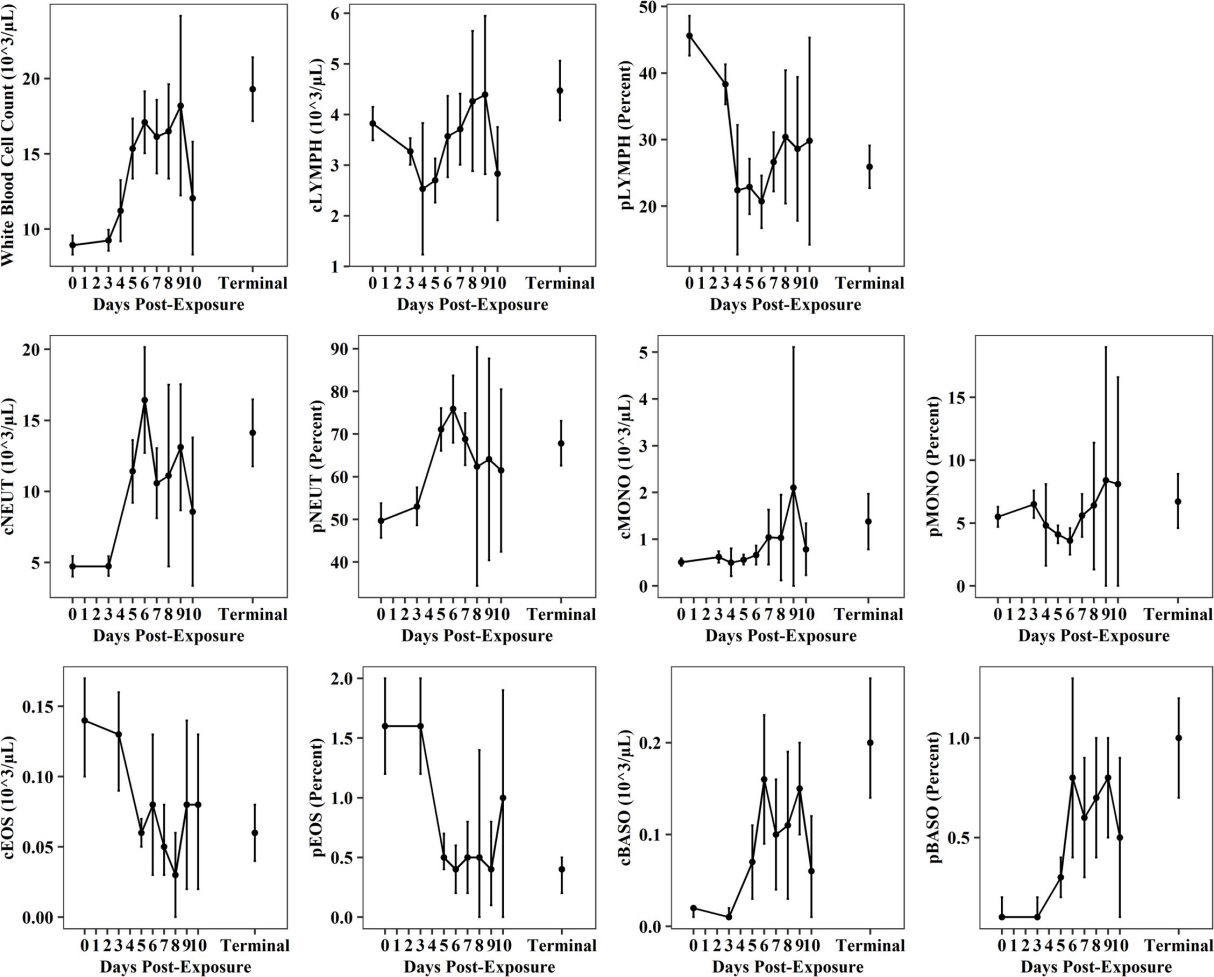
Absolute (c) and differential (p) white blood cell profiles post-EBOV exposure. Plots show data (mean and 95%CI) from all meta-analysis studies for total white blood cell counts, lymphocytes (LYMPH), neutrophils (NEUT), monocytes (MONO), eosinophils (EOS), and basophils (BASO).

Changes in white blood cell profiles observed as early as PED 4–5 were indicative of an acute inflammatory response post-exposure (Fig 5, S10–S20 Tables). From baseline through terminal collections, total WBC counts increased 2-fold from 8.94 ± 3.32 (N_0_ = 106) to 19.29 ± 8.88 10^3/μL (N_T_ = 70; Fig 5, S10 Table) and also increased in variability. Terminal WBC counts were elevated compared to normal ranges for CMs (11.42–11.51 10^3/μL) [26]. Absolute neutrophil counts (cNEUT) also increased rapidly post-exposure (Fig 5, S13 Table), with a 3-fold increase from baseline 4.73 ± 2.84 (N_0_ = 61) to 14.12 ± 7.75 10^3/μL (N_T_ = 44) and when compared to normal ranges for CMs (4.19–5.19 10^3/μL) [26]. Percentage of neutrophils (pNEUT, Fig 5, S14 Table) increased from 49.7 ± 15.5% (N_0_ = 59) to 67.8 ± 17.3% (N_T_ = 44) and were elevated compared to normal ranges for CMs (48.4–50.7%) [26]. Baseline absolute lymphocyte counts (cLYMPH) were lower than normal ranges reported for CMs (4.91–5.19 10^3/μL) [26]. Although small decreases were observed immediately post-exposure, comparison between baseline (3.82 ± 1.72 10^3/μL, N_0_ = 106) and terminal collections (4.47 ± 2.48 10^3/μL, N_T_ = 70) indicated similar counts (Fig 5, S11 Table). On PED 0 and at terminal collections, the percentage of lymphocytes (pLYMPH) decreased from 45.6 ± 15.1% (N_0_ = 98) to 25.9 ± 13.1% (N_T_ = 67; Fig 5, S12 Table), respectively, and were also decreased when compared to normal ranges for CMs (45.2–47.5%) [26]. Decreased pLYMPH were largely attributed to proportional shifts in relation to increased pNEUT. Notably, both the absolute count (cBASO) and percentage of basophils (pBASO) in peripheral blood increased 10-fold post-exposure (Fig 5, S19 and S20 Tables) and were also higher than reference values reported for CMs (0.04 10^3/μL; 0.3%) [26]. From PED 0 to terminal collections, cBASO increased from 0.02 ± 0.01 (N_0_ = 61) to 0.20 ± 0.22 10^3/μL (N_T_ = 44) and pBASO increased from 0.1 ± 0.1% (N_0_ = 61) to 1.0 ± 0.8% (N_T_ = 44). There were minimal changes in monocytes (Fig 5, S15 and S16 Tables) and eosinophils (Fig 5, S17 and S18 Tables) post-exposure. From PED 0 to terminal collections, the monocyte counts(cMONO) increased from 0.51 ± 0.41 (N_0_ = 104) to 1.38 ± 2.48 10^3/μL (N_T_ = 68) and the percentage of monocytes (pMONO) increased from 5.5 ± 3.8% (N_0_ = 96) to 6.7 ± 8.6% (N_T_ = 65). Baseline and terminal concentrations of absolute and percent monocytes were somewhat higher than normal values reported for CMs (0.28–0.30 10^3/μL; 2.5–2.7%) [26]. Eosinophil counts (cEOS) decreased from 0.14 ± 0.13 (N_0_ = 61) to 0.06 ± 0.06 10^3/μL (N_T_ = 44); and percent eosinophils (pEOS) decreased from 1.6 ± 1.6% (N_0_ = 61) to 0.4 ± 0.5% (N_T_ = 44). Terminal concentrations of absolute and percent eosinophils were slightly lower than reference values reported for CMs (0.07 10^3/μL; 0.6–0.7%) [26].

**Fig 6:**
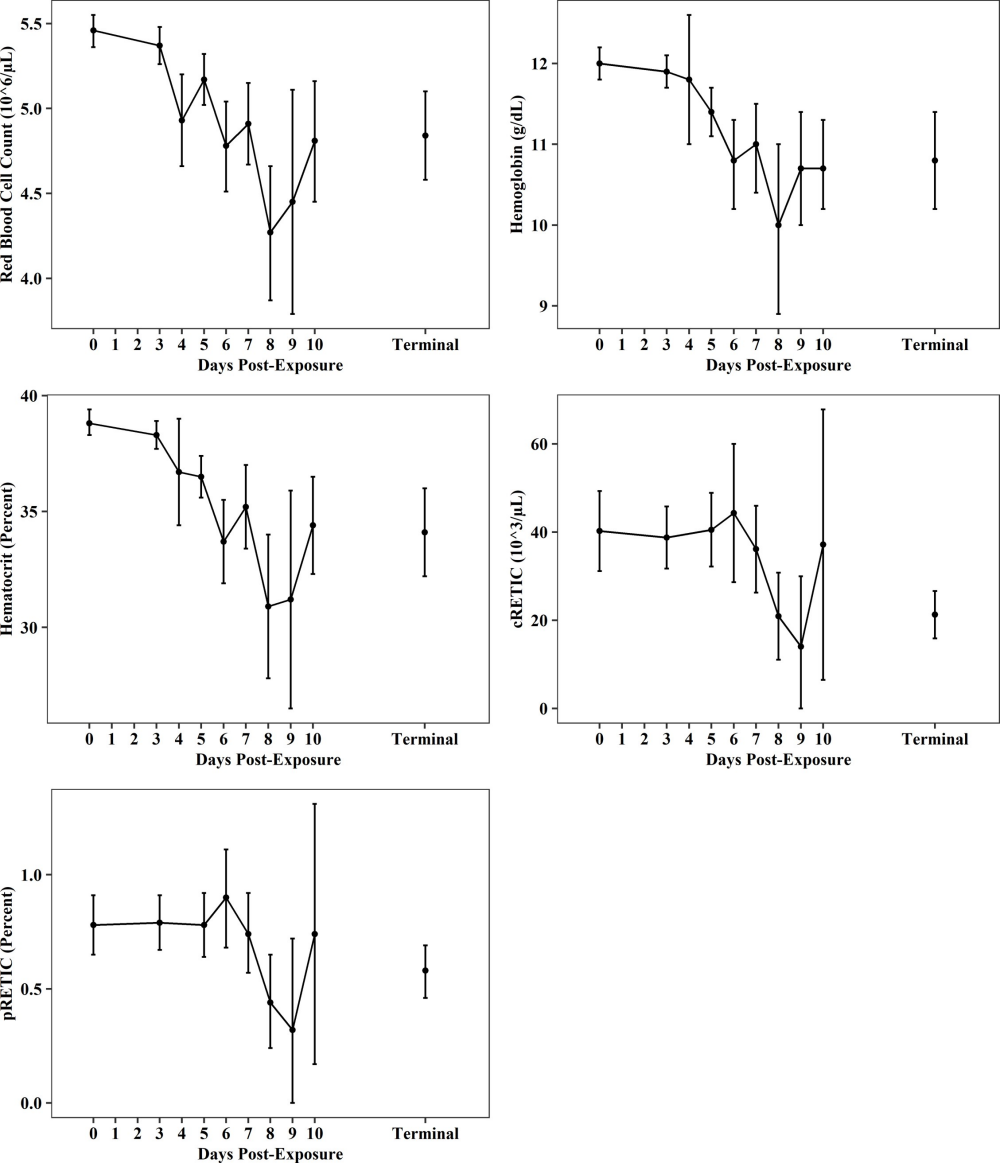
Red blood cell parameters post-EBOV exposure. Plots shows data (mean and 95%CI) from all meta-analysis studies for total red blood cell counts, hemoglobin, hematocrit, total reticulocytes (cRETIC), and percent reticulocytes (pRETIC).

Changes in red blood cell concentrations were observed as early as PED 5, with minimum concentrations typically reached on PED 8 or 9 (Fig 6, S21–S25 Tables). Total red blood cell counts (RBC), hemoglobin (Hb), and hematocrit ratios (HCT) generally decreased 10–12% from baseline through terminal collections. From PED 0 through terminal collections, total RBC counts decreased from 5.46 ± 0.48 (N_0_ = 106) to 4.84 ± 1.1 10^6/μL (N_T_ = 70; Fig 6, S21 Table), Hb decreased from 12.0 ± 1.0 g/dL (N_0_ = 104) to 10.8 ± 2.4 g/dL (N_T_ = 68; Fig 6, S22 Table), and hematocrit decreased from 38.8 ± 2.8% (N_0_ = 106) to 34.1 ± 8.0% (N_T_ = 70; Fig 6, S23 Table). Baseline values for RBC, Hb, and HCT were generally lower than reference values reported for CMs (RBC: 5.89–5.58 10^6/μL; Hb:13.5–13.9 g/dL; HCT: 44.3–45.7%) [26]. Mean values of reticulocytes (cRETIC) at were largely unchanged through PED 7; thereafter, reticulocytes were decreased compared to baseline. From PED 0 through terminal collections, cRETIC decreased nearly 50% from 40.22 ± 28.0 (N_0_ = 39) to 21.26 ± 13.04 10^3/μL (N_T_ = 25; Fig 6, S24 Table) and the percentage of reticulocytes (pRETIC) in peripheral blood decreased from 0.78 ± 0.48% (N = 58) to 0.58 ± 0.37% (N_T_ = 42; Fig 6, S25 Table). Baseline values (PED 0) for cRETIC and pRETIC were lower than normal ranges reported for CMs (cRETIC: 73.5–79.8 10^3/μL; pRETIC: 1.25–1.44%) [26]. All other RBC parameters either showed no changes post-exposure, or sample size was too small to draw meaningful conclusions. Specifically, there were no changes noted for cellular hemoglobin content (CHC), mean corpuscular volume (MCV), mean corpuscular hemoglobin (MCH), or mean corpuscular hemoglobin concentration (MCHC).

**Fig 7:**
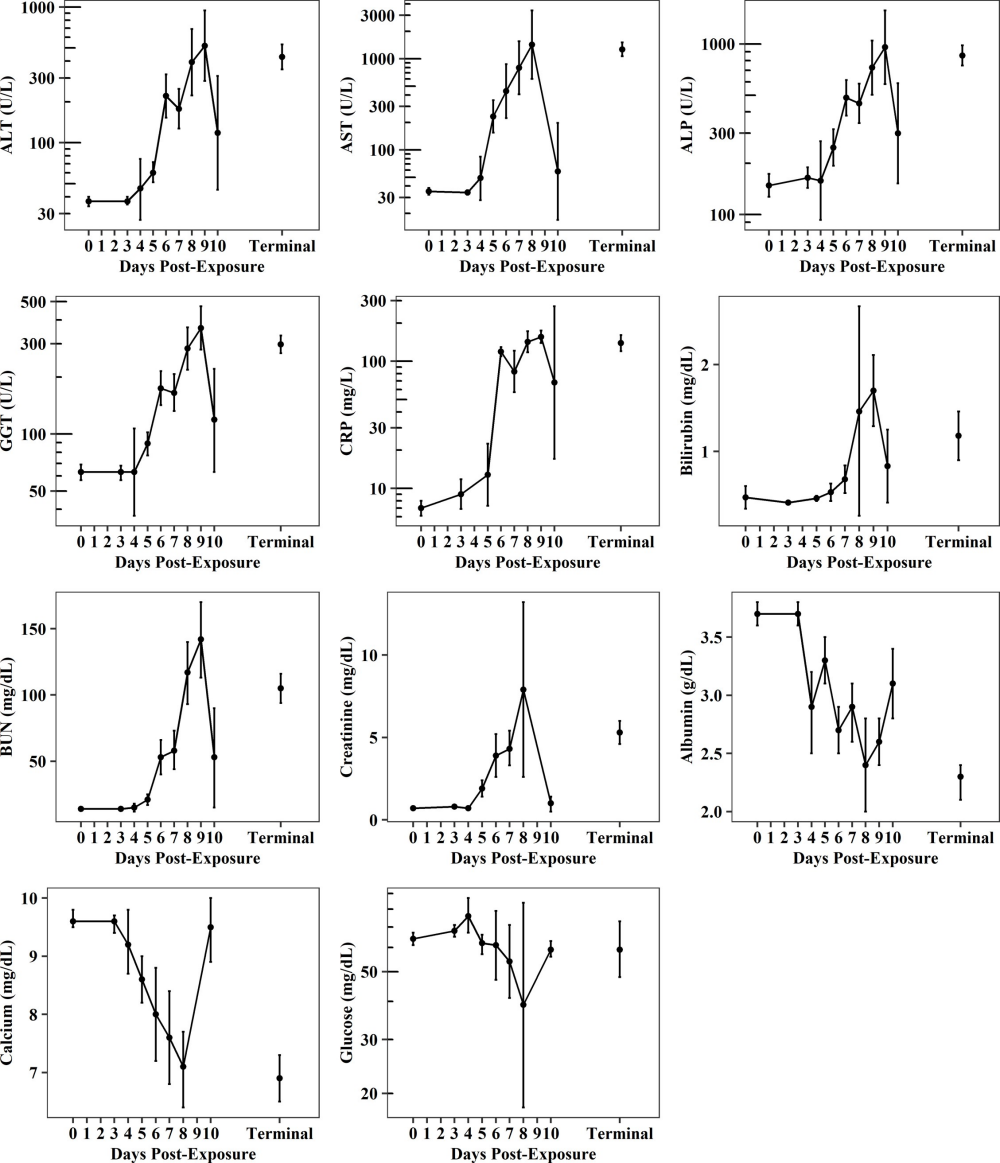
Serum chemistry parameters post-EBOV exposure. Plots shows geometric mean and 95%CI from all meta-analysis studies for alanine aminotransferase (ALT), aspartate aminotransferase (AST), alkaline phosphatase (ALP), C-reactive protein (CRP), gamma-glutamyl transferase (GGT) and glucose, and mean and 95% CI for bilirubin, blood urea nitrogen (BUN), creatinine, albumin, and calcium.

Increased liver enzymes, including alanine aminotransferase (ALT), aspartate aminotransferase (AST), alkaline phosphatase (ALP), and gamma-glutamyl transferase (GGT), were observed shortly after exposure (Fig 7, S26–S29 Tables). Changes were generally several-fold higher than baseline beginning on PED 5, with maximum levels typically reached on or near PED 8–9. From PED 0 through terminal collections, GM ALT increased from 37 U/L (44%CV, N_0_ = 104) to 429 U/L (103%CV, N_T_ = 64); GM AST increased from 35 U/L (30% CV, N_0_ = 50) to 1267 U/L (51%CV, N_T_ = 31); GM ALP increased from 148 U/L (95%CV, N_0_ = 104) to 856 U/L (62%CV, N_T_ = 70); and GM GGT increased from 63 U/L (51%CV, N_0_ = 104) to 297 (47%CV, N_T_ = 68). Although baseline concentrations of ALT, AST, and ALP were lower than normal values (ALT: 51.8–60.0 IU/L; AST: 61.4–62.7 IU/L; ALP: 1059.4–1283.7 IU/L; GGT: 46.32–64.68 IU/L) [26], changes from baseline were also several-fold higher than reference ranges. Increased serum concentrations of C-reactive protein (CRP) and bilirubin were further indications of liver changes post-exposure (Fig 7, S30 and S31 Tables). From PED 0 through terminal collections, CRP increased nearly 20-fold from 7.0 mg/L (27.3%CV, N_0_ = 17) to 139.0 mg/L (30.8%CV, N_T_ = 19); and total bilirubin increased from 0.47 U/L (0.60%CV, N_0_ = 88) to 1.18 U/L (1.07%CV, N_T_ = 59). Total bilirubin concentrations were also several-fold higher compared to reference values (total bilirubin 0.213–0.269 U/L) [26]. Increased serum concentrations of BUN and creatinine were indications of kidney changes post-exposure (Fig 7, S32 and S33 Tables). BUN increased 7-fold from 14 ± 7 mg/dL (N_0_ = 103) to 105 ± 44 mg/dL (N_T_ = 63); and creatinine levels increased 7-from 0.7 ± 0.2 mg/dL (N_0_ = 54) to 5.3 ± 2 mg/dL (N_T_ = 34). Changes were also several fold higher than normal ranges (BUN 22.6–25.5 mg/dL; creatinine 0.90–0.94 mg/dL) [26]. In the CM model, blood calcium levels typically decline during EVD progression and in conjunction with increased blood urea and creatinine levels [27]. Our results are consistent with these changes. Although baseline calcium levels were lower than reference values reported for CMs (calcium 10.52–10.63 mg/dL) [26], calcium decreased from baseline (9.6 ± 0.4 mg/dL, N_0_ = 54) through terminal collections (6.9 ± 1.1 mg/dL, N_T_ = 28; Fig 7, S34 Table). Decreased serum concentrations of albumin and glucose were observed post-exposure (Fig 7, S35 and S36 Tables). From PED 0 through terminal collections, albumin decreased from 3.7 ± 0.6 g/dL (N_0_ = 103) to 2.3 ± 0.6 g/dL (N_T_ = 65); and was also decreased compared to normal values (albumin 4.47–4.55 g/dL) [26]. On PEDs 7 and 8 GM glucose concentrations decreased from 64 mg/dL (17%CV, N_0_ = 54) at PED 0 to 54 mg/dL (67%CV, N_7_ = 22) at PED 7 and 39 (69%CV, N_8_ = 5) at PED 8; overall, however, levels were similar to baseline at terminal collections (59 ± mg/dL; N_T_ = 33).

**Fig 8:**
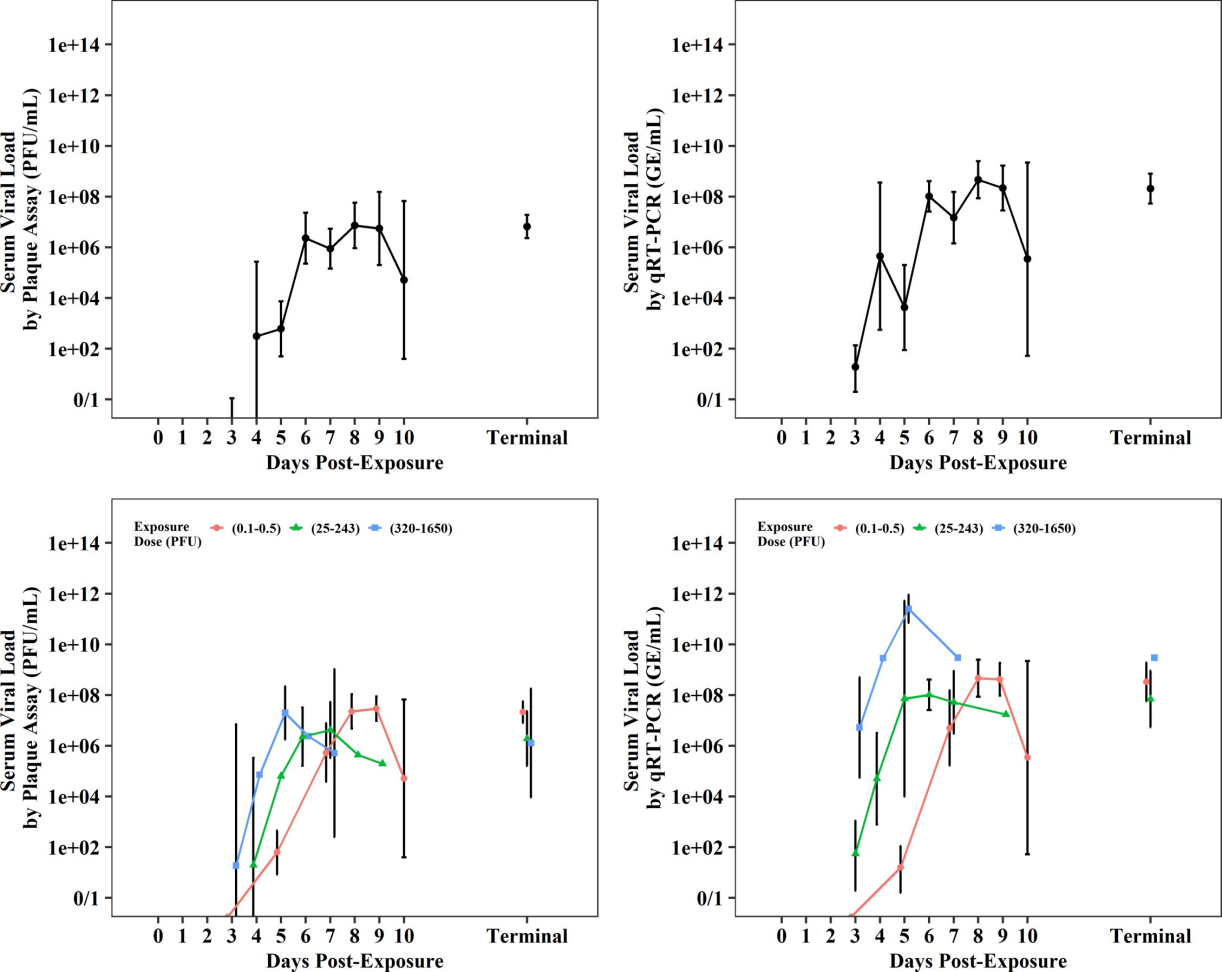
Serum viral load post-EBOV exposure measured by plaque assay (PFU/mL) displayed on left and qRT-PCR (GE/mL) displayed on right. Plots shows data (geometric mean and 95%CI) from all meta-analysis studies as well as differences observed by exposure dose.

**Fig 9:**
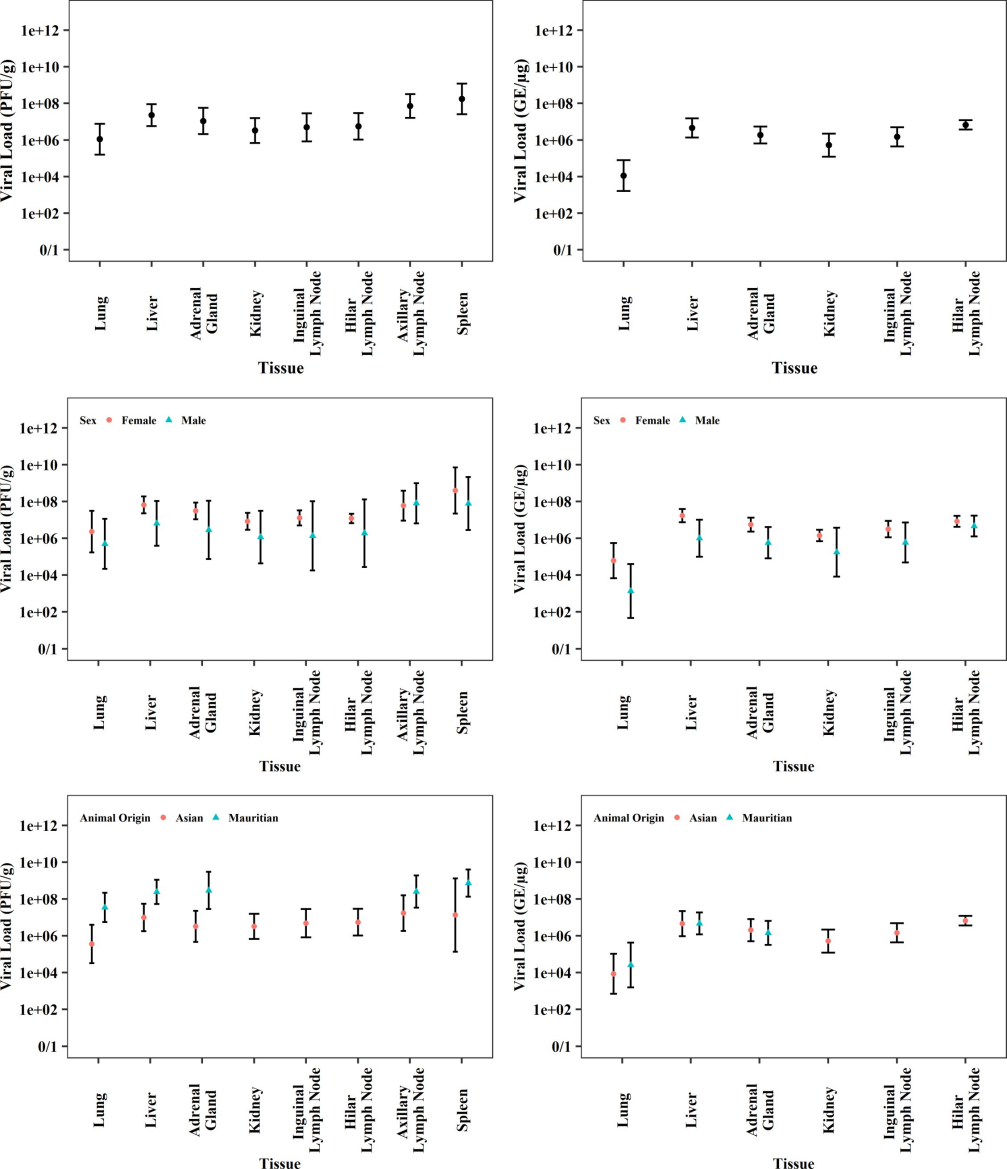
**Tissue viral load collected from CMs at termination measured by plaque assay (PFU/g) displayed on left and qRT-PCR (GE/μg) displayed on right.** Plots shows data (geometric mean and 95%CI) from all meta-analysis studies as well as differences observed by sex and animal origin.

Viral loads in blood (serum) and selected tissues were measured using plaque assays and qRT-PCR (S37–S46 Tables). Standardization across studies was not possible, but distinct trends were noted over time in both assays. The onset of active viremia was observed within a few days of infection, as serum viral loads increased by several orders of magnitude from PED 3–4 through PED 6–8 (Fig 8, S37 and S39 Tables). Although based on small sample sizes, serum viral loads appeared to be dose-dependent whereby viral loads peaked earlier at higher exposure doses (Fig 8, S38 and S40 Tables). Tissue viral load data were limited to IM exposures performed at two ABSL-4 facilities and represented terminal collections from PED 6–12. The geometric means across the tissues spanned roughly two orders of magnitude in both assays, with lowest viral loads in lung tissues. Tissue viral loads tended to be somewhat higher for females when compared to males and higher for Mauritian when compared to Asian CMs in the plaque assay (Fig 9, S41–S46 Tables). The biological significance of these differences is uncertain due to the low magnitude of differences and/or the detection of the trend in only one of the two assays.

**Fig 10:**
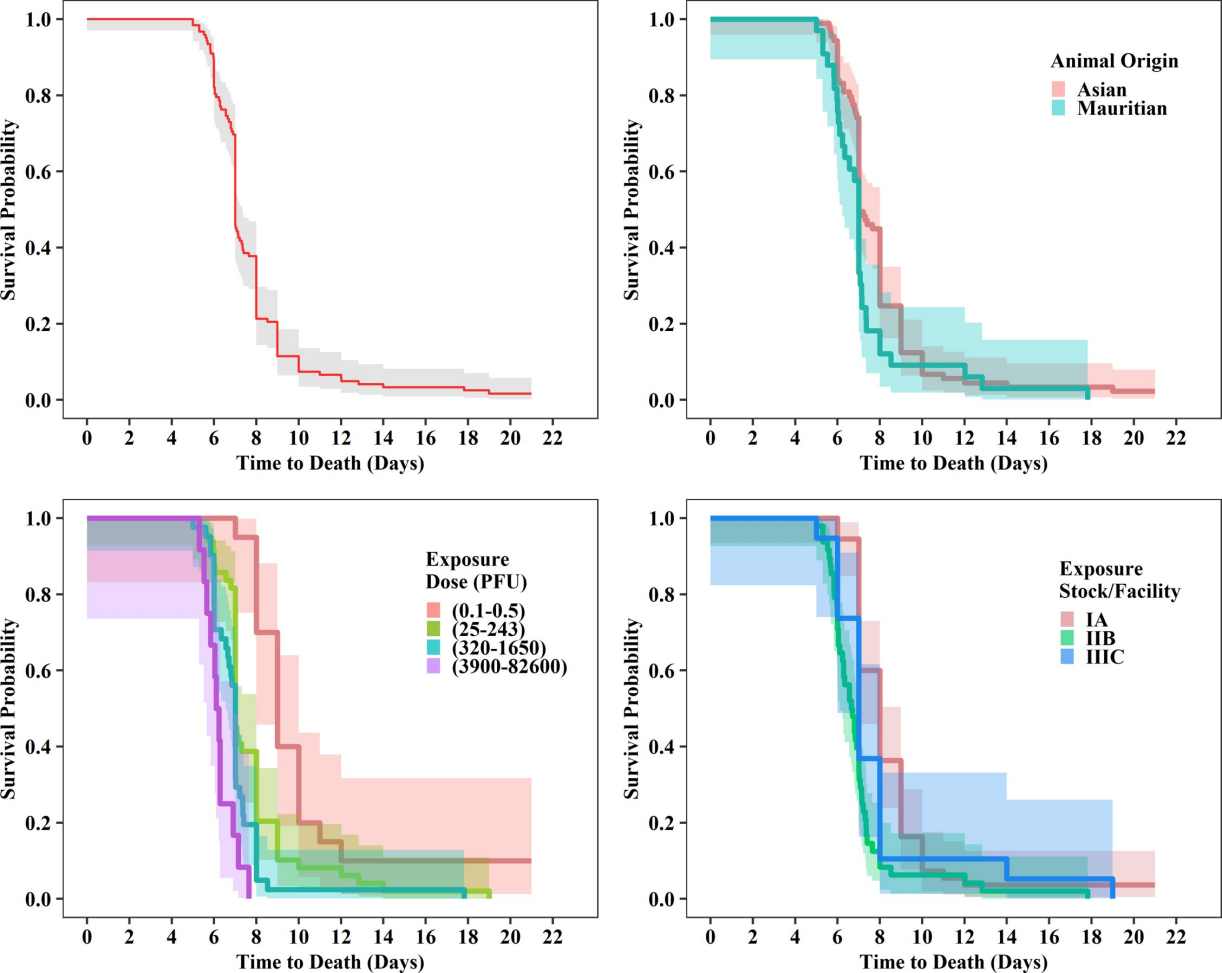
Kaplan-Meier survival probabilities plotted over time. Plots show data from all meta-analysis studies and differences observed by exposure dose, origin, and exposure stock/facility.

The median time to death was 7 days for all categories for animal model characteristics (Table 3) but varied over exposure characteristics (Table 4) from 6.2 to 9.0 days. This range was observed across exposure doses, as the median time to death decreased with increasing dose. For the low (0.1–0.5 PFU), intermediate (25–243 and 320–1650 PFU), and high dose groups (3900–82,60 PFU), median times to death were 9, 7, and 6.2 days, respectively. Additionally, time to death was statistically different across exposure dose levels based on the log rank test (p<0.001). Statistically significant differences in time to death were also identified for animal origin and exposure stock/facility based on log rank tests (Table 5). Kaplan-Meier plots for these parameters show later time to death for CMs of Asian origin and at very low exposure doses, and earlier time to death for CMs in stock/facility IIB studies (Fig 10). Note that the Kaplan-Meier plots and log rank tests do not account for the study-to-study variability and interactions between variables; the Cox PH-frailty models provide a more extensive evaluation of time to death.

**Table 3:** Time to death (days post-exposure) for CM animal model characteristics.

CM Characteristics		N	Median	Min	Max	95% CI
Overall		122	7.0	5.0	21.0	(7.0, 7.3)
Sex	Female	62	7.0	5.0	19.0	(7.0, 8.0)
	Male	60	7.0	5.0	21.0	(7.0, 8.0)
Age^a^	Adults	56	7.0	5.0	21.0	(7.0, 8.0)
	Juveniles	40	7.0	5.3	17.8	(6.9, 8.0)
Animal Origin	Asian	89	7.0	5.0	21.0	(7.0, 8.0)
	Mauritian	33	7.0	5.0	17.8	(6.3, 7.1)
Euthanasia Status^b^	FDIC	25	7.0	5.0	17.8	(6.7, 9.0)
	Primary	51	7.0	5.3	12.0	(7.0, 8.0)
	Secondary	20	7.1	5.7	9.0	(7.0, 7.6)

a. Juvenile = 2–3 years; adult = ≥4 years

b. FDIC = found dead in cage; primary and secondary euthanasia criteria were used qualify moribund animals for humane termination

**Table 4:** Time to death (days post-exposure) for exposure characteristics.

Exposure Characteristics		N	Days (post-exposure)			
			Median	Min	Max	95% CI
Dose (PFU)	0.1–0.5	20	9.0	7.0	21.0	(9.0, 10.0)
	25–243	49	7.0	5.0	19.0	(7.0, 8.0)
	320–1650	41	7.0	5.0	17.8	(6.7, 7.0)
	3900–82600	12	6.2	5.3	7.6	(5.8, NA)
Route^a^	Aerosol	16	7.0	5.6	8.0	(6.8, NA)
	IM	86	7.0	5.0	21.0	(7.0, 7.4)
	IN	20	7.7	5.3	17.8	(6.3, 10.0)
Stock/facility	IA	55	8.0	6.0	21.0	(7.0, 9.0)
	IIB	48	6.7	5.0	17.8	(6.3, 7.0)
	IIIC	19	7.0	5.0	19.0	(7.0, 8.0)

a. IM = intramuscular; IN = intranasal

**Table 5:** Log rank test comparisons of time to death.

Characteristics		Comparison groups	N	Log rank p-value
Animal model	Sex	Male; Female	122	0.6858
	Age^a^	Juvenile; Adult	96	0.1991
	Origin	Asian; Mauritian	122	0.0228*
Control article		PBS/TBS/saline^b^	100	0.8713
		Vaccine buffer/diluent		
		Vaccine vector		
Exposure	Dose	0.1–0.5; 25–243;	122	<0.0001*
		320–1650; 3900–82600 PFU		
	Route^c^	IM; IN; Aerosol	122	0.2014
	Stock/Facility	IA; IIB; IIIC	122	<0.0001*
Euthanasia status^d^		FDIC; Primary; Secondary	96	0.2573

a. Juvenile = 2–3 years; adult = ≥4 years

b. PBS = phosphate buffered saline; TBS = Tris-buffered saline

c. IM = intramuscular; IN = intranasal

d. FDIC = found dead in cage; primary and secondary euthanasia criteria were used qualify moribund animals for humane termination

* Variable is statistically significant (p<0.05) in the model.

Table 6 summarizes the individual Cox PH-frailty models for each predictor variable fit to all available data and for the subset of CM exposed to 100 to 1,000 PFU. The number of CMs included in each model varied due to missing data for some of the predictor variables. Demographic variables (sex, age, animal origin), control article, and euthanasia criteria were not statistically significant and hazard ratios were near 1. Animal origin had been identified as a potential predictor of time to death based on the log rank test but was not significant in the Cox PH-frailty model that accounted for study as a random effect. The HR>1, which suggests higher risk for CM of Mauritian origin (consistent with the log rank test), but 95% confidence intervals were wide. Individually (Table 6), all exposure characteristics had statistically significant effects in the model fit to all data. Hazard ratios and confidence intervals were similar between models fit to all available data and the subset of CM exposed to 100 to 1,000 PFU, so differences in significant findings are attributed to reduced sample size in the subset analysis. Therefore, the following discussion is based on the model fit to all data. For exposure route, the IN route had significantly lower risk than IM, while risk for the IM route was not significantly different from aerosol. Similarly, exposure stock/facility IA had lower risk than IIB, while other combinations were not statistically different. Finally, there was significantly greater risk at higher doses. The hazard ratio for log10 dose is 2.09 which means that the risk of death is increased by 2.09-fold when the log10 dose is increased by 1 unit. A 1 unit increase in log10 dose corresponds to a 10-fold increase in dose, consistent with the change from 10 to 100 or 100 to 1,000 PFU.

**Table 6:** Univariate Cox PH-frailty models fit to all CM and to a subset of CM exposed to estimated target doses of 100 to 1,000 PFU.

Effect	Comparison group	Reference group	All CM		CM exposed to	
					100 to 1,000 PFU	
			N	Hazard Ratio	N	Hazard Ratio
				(95% CI)		(95% CI)
Sex	Male	Female	122	0.95 (0.64, 1.4)	90	1.27 (0.8,2.02)
Age	Age (years)	NA	96	0.92 (0.71, 1.18)	64	1.04 (0.78,1.39)
Origin	Mauritian	Asian	122	1.45 (0.63,3.36)	90	1.38 (0.62,3.07)
Control Article	Vaccine Buffer/Diluent	PBS/TBS/Saline^a^	100	0.83 (0.29,2.38)	88	0.90 (0.25,3.27)
	Vaccine Vector	PBS/TBS/Saline	100	0.97 (0.54,1.74)	88	0.92 (0.44,1.94)
Exposure Dose	Log10(dose)	NA	122	2.09 (1.5,2.92)*	90	1.42 (0.81,2.5)
Route^b^	Aerosol	IM	122	1.05 (0.46,2.37)	90	0.99 (0.51,1.95)
	IN	IM	122	0.28 (0.1,0.81)*	90	0.26 (0.12,0.59)*
Stock/Facility	IA	IIB	122	0.37 (0.17,0.81)*	90	0.39 (0.17,0.89)*
	IIIC	IIB	122	0.43 (0.18,1)	90	0.39 (0.14,1.04)
Euthanasia	Primary	FDIC	96	1.22 (0.69,2.15)	66	1.19 (0.62,2.28)
Criteria^c^	Secondary	FDIC	96	1.04 (0.52,2.09)	66	0.86 (0.39,1.87)

a. PBS = phosphate buffered saline; TBS = Tris-buffered saline

b. IM = intramuscular; IN = intranasal

c. FDIC = found dead in cage; primary and secondary euthanasia criteria were used qualify moribund animals for humane termination

* Variable is statistically significant (p<0.05) in the model.

NA–Not Applicable.

**Fig 11:**
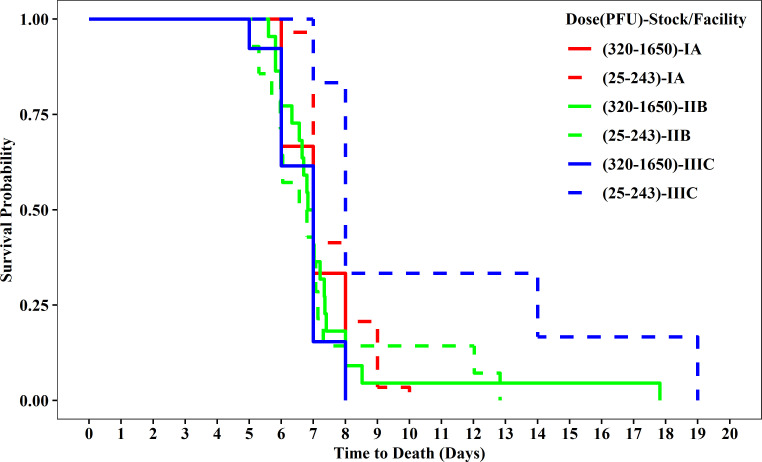
Kaplan-Meier survival probabilities over time for subset of CMs exposed to 100 to 1,000 pfu, by exposure dose group and exposure stock/facility.

Based on the univariate Cox PH-frailty models, only the exposure variables (route, stock/facility, and log10 dose) were statistically significant predictors of time to death. The next Cox PH-frailty models (Table 7) considered these exposure variables and interactions between exposure dose and the other variables in the fixed effects portion of the model. After accounting for the other predictors, only log10 dose, exposure stock/facility (IIIC compared to IIB), exposure route (IN route compared to IM), and the interaction between log10 dose and stock/facility, expressed as a difference in dose effects between IIIC and IIB, were significant in the model fit to all data. With the exception of log10 dose, the same effects were significant in the model fit to the subset of CM exposed to 100 to 1,000 PFU although the hazard ratios varied more between these models than the corresponding individual models. The log10 dose effect must be interpreted together with the interaction term with dose effect because changes in the interaction effect modify the overall dose effect. The hazard ratios for exposure routes were consistent with those in the individual models. The interaction between exposure dose and stock/facility indicates that for a 10-fold increase in exposure dose, the risk of death for exposure stock/facility IIIC is increased compared to exposure stock/facility IIB after adjusting for other variables in the model. Fig 11 displays a Kaplan-Meier survival plot of the 25 to 243 PFU and 320 to 1650 PFU exposure dose groups at each exposure stock/facility. Although the Cox PH-frailty models did not rely on the dose groups, the Kaplan-Meier plot is useful in interpreting the interaction. At lower doses, the time to death was much later for stock/facility IIIC than the other exposure stock/facilities and stock/facility IIIC at higher doses. The significant model effect and interaction can be attributed to this difference. Although the effects of virus stock cannot be separated from other differences between exposure facilities, note that virus stock III was passage 2 from the original isolate and the parent stock for the other virus stocks.

**Table 7:** Multivariate Cox PH-frailty models fit to all CM and to a subset of CM exposed to estimated target doses of 100 to 1,000 PFU.

Effect	Comparison group	Reference group	All CM	CM exposed to 100 to 1,000 PFU
			(N = 122)	(N = 90)
			Hazard ratio	Hazard ratio
			(95% CI)	(95% CI)
Exposure Dose	Log10(dose)	IM, IIB	2.01 (1.23,3.30)*	0.81 (0.38,1.74)
Stock/Facility	IIIC	IIB	1.04 (0.22,4.86)	0.20 (0.01,2.86)
	IA	IIB	0.01 (0.00,0.51)*	0.00 (0.00,0.11)*
Route^a^	Aerosol	IM	0.86 (0.46,1.61)	0.82 (0.43,1.58)
	IN	IM	0.28 (0.15,0.52)*	0.23 (0.11,0.48)*
Interaction:	IIIC	IIB	0.83 (0.49,1.40)	1.46 (0.47,4.54)
Dose × Stock/Facility	IA	IIB	4.13 (1.03,16.64)*	9.48 (1.82,49.50)*

a. IM = intramuscular; IN = intranasal

* Variable is statistically significant (p<0.05) in the model.

The final models considered clinical pathology biomarkers of inflammation or coagulation disruption, which were added individually to the full model as time-dependent covariates. After accounting for the exposure variables included in the models, biomarkers of coagulation disruption (PTT, PLT, MPV), inflammation (WBC and differentials), red blood cells (hemoglobin, hematocrit, reticulocytes, and RBC distribution width), liver enzymes (ALT, AST, ALP, GGT, bilirubin, BUN, and creatinine), and other serum chemistry markers (albumin, calcium, and glucose) were significant predictors of death (Table 8). As before, hazard ratios and confidence intervals were similar between models fit to all available data and the subset of CM exposed to 100 to 1,000 PFU, so differences in significant findings are attributed to reduced sample size in the subset analysis.

**Table 8:** Multivariate Cox PH-frailty models with a single time dependent covariate fit to all CM and to a subset of CM exposed to estimated target doses of 100 to 1,000 PFU.

Time Dependent Covariate	Unit	All CM			CM exposed to 100 to 1,000 PFU		
		N	Total Obs	Hazard ratio	N	Total Obs	Hazard ratio
				(95% CI)			(95% CI)
PTT^	seconds	65	225	2.51 (1.27, 4.97)*	39	122	2.02 (0.88, 4.63)
PLT^	10^3/μL	109	355	0.74 (0.63, 0.87)*	77	229	0.78 (0.65, 0.94)*
MPV	fL	113	370	1.23 (1.07, 1.41)*	81	244	1.15 (0.98, 1.35)
White Blood Cell Count^	10^3/μL	113	371	2.45 (1.78, 3.39)*	81	245	2.47 (1.67, 3.65)*
Lymphocyte Count^	10^3/μL	113	371	1.19 (0.94, 1.51)	81	245	1.31 (0.98, 1.76)
Lymphocyte Percent	(%)	105	340	0.98 (0.97, 1)*	73	214	0.99 (0.97, 1.01)
Neutrophil Count^	10^3/μL	65	235	1.94 (1.44, 2.61)*	33	109	2.44 (1.43, 4.16)*
Neutrophil Percent	(%)	63	229	1.02 (1.01, 1.04)*	31	103	1.04 (1, 1.07)*
Monocyte Count^	10^3/μL	111	367	1.07 (0.89, 1.29)	79	241	1.11 (0.88, 1.39)
Monocyte Percent	(%)	103	335	0.99 (0.94, 1.05)	71	210	1.00 (0.93, 1.07)
Eosinophil Count^	10^3/μL	65	234	0.85 (0.71, 1.01)	33	109	0.94 (0.75, 1.19)
Eosinophil Percent	(%)	65	235	0.37 (0.23, 0.6)*	33	109	0.32 (0.15, 0.68)*
Basophil Count^	10^3/μL	65	235	1.98 (1.58, 2.47)*	33	109	1.6 (1.18, 2.17)*
Basophil Percent	(%)	65	235	1.51 (1.08, 2.1)*	33	109	1.1 (0.76, 1.6)
Red Blood Cell Count	10^6/μL	113	371	0.87 (0.64, 1.2)	81	245	1.27 (0.77, 2.1)
Hemoglobin	g/dL	111	366	0.79 (0.66, 0.94)*	79	240	0.89 (0.69, 1.13)
Hematocrit	(%)	113	371	0.94 (0.89, 1)*	81	245	0.98 (0.9, 1.06)
Reticulocyte Count^	10^3/μL	41	160	0.64 (0.45, 0.93)*	15	56	0.94 (0.43, 2.04)
Reticulocyte Percent	(%)	63	229	0.71 (0.39, 1.29)	31	104	1.62 (0.64, 4.12)
RDW	(%)	109	359	1.3 (1.14, 1.49)*	77	233	1.18 (1.01, 1.37)*
ALT^	U/L	109	363	1.56 (1.35, 1.8)*	77	237	1.41 (1.18, 1.68)*
AST^	U/L	56	175	1.68 (1.37, 2.05)*	44	137	1.66 (1.33, 2.09)*
ALP^	U/L	109	364	1.86 (1.49, 2.34)*	77	238	1.6 (1.24, 2.06)*
GGT^	U/L	109	364	2.33 (1.81, 3)*	77	238	1.93 (1.44, 2.59)*
Bilirubin	mg/dL	90	311	1.52 (1.09, 2.1)*	58	187	1.41 (0.96, 2.05)
BUN^	mg/dL	109	361	2.58 (2.02, 3.29)*	77	235	2.33 (1.75, 3.11)*
Creatinine	mg/dL	60	188	1.52 (1.33, 1.75)*	48	148	1.68 (1.41, 2)*
Albumin	g/dL	109	361	0.23 (0.14, 0.36)*	77	235	0.3 (0.17, 0.52)*
Calcium	mg/dL	60	184	0.65 (0.52, 0.81)*	48	146	0.64 (0.5, 0.83)*
Glucose^	mg/dL	60	189	0.34 (0.19, 0.62)*	48	148	0.24 (0.11, 0.54)*

* Variable is statistically significant (p<0.05) in the model.

^ Log base 2 was used; hazard ratio indicates change in hazard associated with 2-fold change in response.

## Discussion

Non-human primates can be lethally infected with non-adapted human isolates of EBOV and show a high degree of similarity with human pathogenesis in both the etiology of disease (e.g., virulence) and in the progression of disease symptoms [28], although the pathogenesis and time to death are more rapid in NHP, most likely due to the high exposure doses administered. EBOV being a highly virulent Category A priority pathogen [29], a highly conserved approach is needed in the characterization of mechanisms and developing therapeutic agents for EBOV infection in humans. Under the conditions described across exposure studies, the CM model is well aligned with meeting these objectives.

In the CM sham-vaccinated control population, viral loads in circulating blood were observed within 3–5 days of the initial infection, resulting in rapid disease progression and humane euthanasia or death generally within 5–12 days post-infection. Reduced activity and responsiveness were observed beginning 4–6 days after infection and usually preceded death or euthanasia by approximately 1 day. Notably, median time to death estimates were dose-dependent, ranging between 6.2 to 9 days. In humans, an incubation period of 2–21 days (typically 5–10 days) is generally followed by an abrupt but non-specific viral syndrome characterized by fever, chills, and myalgia [30]. The incubation period was abbreviated for CMs in the sham-vaccinated control population, likely due to the high exposure doses; however, rapid progression of disease was evident, as described by changes in body temperature and blood profiles, particularly those indicative of coagulation disruption. In humans, viremia kinetics are important bioindicators of infection and differences in trends between survivors and non-survivors have been well documented in the available literature following EBOV outbreaks [31–35]. In the sham-vaccinated control population, all but two CMs were non-survivors; therefore, comparison of viremia between survivors and non-survivors was not possible. Beginning on PED 4, substantial increases in serum viral loads were observed. Most notably, serum data exhibited dose-dependent viremia whereby viral loads were detected and peaked earlier at higher exposure doses. In tissue, dose-dependent viral loads were less clear, but were consistent with rapid disease progression.

Clinical signs of EBOV infection in CMs can occur within 4–5 days of the initial infection [27, 36]. Typically, CMs become lethargic and less responsive, and a decrease in body temperatures usually precedes deaths [27]. For both humans and non-human primates, EVD-induced weight loss is typically associated with dehydration, although mobilization of body reserves and catabolism can also be contributing factors. Body weight did not fluctuate in the sham-vaccinated control population and clinical signs were not summarized to describe gastrointestinal distress typically associated with EVD-induced intestinal hemorrhage. Reduced activity and responsiveness were observed in most animals by PED 6. Fever was not clinically observed on average; after PED 5, however, body temperatures decreased on average and variability increased as the first animals met euthanasia criteria. Terminal body temperatures collected at the time of euthanasia (PED 5–12) were well below baseline values, a likely indicator of moribundity (i.e., hemorrhage, lowered cardiac output).

In the sham-vaccinated control population, changes in circulating white blood cell populations, particularly leukocytopenia and increased C-reactive protein, were indicative of an acute inflammatory response by PED 4–5. In addition to leukopenia, lower platelet counts and elevated liver enzymes (e.g., ALT, ALP), are indications of hematologic and liver dysfunction commonly associated with EBOV infection. As the disease progresses, severe lymphopenia, thrombocytopenia, and prolongation of the PTT have been reported as early as day 6 post-infection, and by 10–12 days post infection, blood samples may be unable to clot [37]. In the sham-vaccinated control population, prolongation of PT and PTT were first observed on PED 6 along with increased D-dimer, suggestive of fibrinolysis, and decreased platelets. These results suggest PED 6 as the onset for early stages of coagulation disruption and potential hemorrhage in the CM model. Filovirus infection in human and non-human primates typically results in prolongation of PT and PTT, circulating, circulating fibrin degradation products, and decreased fibrin deposition [28, 38, 39]. As early as 5 days post EBOV infection, extensive EBOV-related gastrointestinal changes attributed to extensive hemorrhage in the duodenum and colon have also been reported in the NHP IM rhesus Kikwit model with mean viral loads of 8.68 log 10 copies/mL EBOV on PED 5 [40]. In the sham-vaccinated control population reported here, decreased hematocrit ratios and diminished circulation of red blood cells, reticulocytes, and hemoglobin were observed beginning on PED 5, suggestive of active hemorrhage in the CM model.

Increased liver enzymes (ALT, AST, ALP, GGT), C-reactive protein (CRP), bilirubin, BUN, and creatinine levels were observed shortly after exposure, generally by PED 5. These elevations were often several-fold higher than baseline and reference values reported for CMs [26]. Moderate decreases in glucose, albumin, and calcium were also noted when compared to baseline and reference values [26]. Decreased calcium and albumin (PED 4–5 through termination) are suggestive of hemorrhage, as calcium is a key cofactor in the coagulation cascade and is involved in platelet function [41]. Blood sodium, potassium and calcium levels typically decline in the CM model during EVD progression in conjunction with increased BUN and creatinine levels [27], and at approximately 5 days post-EBOV infection, liver transaminases (AST and ALT) can increase and remain elevated through termination of the CM [37, 42]. In the sham-vaccinated control population, elevated liver enzymes were attributed to active coagulation disruption, as the liver is the site for synthesis and metabolism of fibrinolytic factors and various blood-clotting factors including fibrinogen [43]. These results are consistent with human pathogenesis, as the final stages of disease in humans are characterized by coagulopathy and vascular leakage resulting in hemorrhage and shock [44].

Due to the targeted routes of exposure at high dosages, exposure studies in CM generally result in higher mortality rates and accelerated time to death [45]. Comparisons of time to death between CM and humans is difficult since humans typically seek medical treatment approximately 6 days post-onset of symptoms [45]. Due to this delay, in addition to unknown etiology, dose, or exposure route in humans, viral kinetics and time to onset of symptoms in humans can be difficult to quantify. In the CM model described here, viral kinetics and changes in biomarkers associated with EVD indicate a similar etiology of disease as seen in humans; however, due to difference in dose, exposure route, the ability to describe disease progression in real time results in a more rapid onset of clinical signs, disease progression, and time to death.

## Conclusions

The CM NHP is the most widely used animal model for filovirus (ebolavirus and margburgvirus) vaccine testing and will be required for MCM licensed under the AR. Multiple filovirus vaccine candidates have been evaluated using the CM NHP model; to the best of our knowledge however, animal model development data such as natural history studies supporting the use of CM have not been submitted to the FDA. This study describes a large CM database and demonstrates the consistency of the CM model through descriptive statistics. Using data from a total of 122 sham-vaccinated control CM from 33 studies, we have shown EVD progression occurred within a few days post-exposure in the CM model under varied exposure conditions and although more rapid, was generally similar to reported cases of human EVD. In particular, hematology indices were indicative of early signs of viremia and the propensity for hemorrhage with progression of EBOV viremia. As expected, there was a clear dose-dependent association between time to death and exposure dose, which was evident in both Kaplan-Meier analysis and Cox PH-frailty regression models, but a significant interaction between exposure dose and stock/facilities indicated that the effect of dose on the risk of death varied between exposure stocks/facilities. In the subset of CM exposed to 100 to 1,000 PFU doses most commonly used in vaccine studies and therefore most relevant to the FDA Animal Rule, these significant effects were attributed to later time to death at low doses for one of the virus stocks. Therefore, caution is required when comparing studies at different facilities, virus stocks, and exposure doses. Despite the differences, all CMs exposed to doses of 100 to 1,000 PFU died or met euthanasia criteria within 21 days of exposure, 93% between 5 and 12 days of exposure. Taken together, these factors suggest that the cynomolgus monkey is a reliable animal model for human EBOV infection and disease progression. The sham-vaccinated control database and meta-analysis may be used to support vaccine programs using the Animal Rule.

## Supporting information

S1 Table Descriptive statistics for weight (kg) over time, overall. (DOCX)

S2 Table Descriptive statistics for weight (kg) over time, by sex. (DOCX)

S3 Table Descriptive statistics for weight (kg) over time, by age. (DOCX)

S4 Table Descriptive statistics for temperature (C) over time, overall. (DOCX)

S5 Table Descriptive statistics for temperature (C) over time, by sex. (DOCX)

S6 Table Descriptive statistics for PT (seconds) over time, overall. (DOCX)

S7 Table Descriptive statistics for PTT (seconds) over time, overall. (DOCX)

S8 Table Descriptive statistics for PLT (10^3/μL) over time, overall. (DOCX)

S9 Table Descriptive statistics for MPV (fL) over time, overall. (DOCX)

S10 Table Descriptive statistics for white blood cell count (10^3/μL) over time, overall. (DOCX)

S11 Table Descriptive statistics for cLYMPH (10^3/μL) over time, overall. (DOCX)

S12 Table Descriptive statistics for pLYMPH (percent) over time, overall. (DOCX)

S13 Table Descriptive statistics for cNEUT (10^3/μL) over time, overall. (DOCX)

S14 Table Descriptive statistics for pNEUT (percent) over time, overall. (DOCX)

S15 Table Descriptive statistics for cMONO (10^3/μL) over time, overall. (DOCX)

S16 Table Descriptive statistics for pMONO (percent) over time, overall. (DOCX)

S17 Table Descriptive statistics for cEOS (10^3/μL) over time, overall. (DOCX)

S18 Table Descriptive statistics for pEOS (percent) over time, overall. (DOCX)

S19 Table Descriptive statistics for cBASO (10^3/μL) over time, overall. (DOCX)

S20 Table Descriptive statistics for pBASO (percent) over time, overall. (DOCX)

S21 Table Descriptive statistics for red blood cell count (10^6/μL) over time, overall. (DOCX)

S22 Table Descriptive statistics for hemoglobin (g/dL) over time, overall. (DOCX)

S23 Table Descriptive statistics for hematocrit (percent) over time, overall. (DOCX)

S24 Table Descriptive statistics for cRETIC (10^3/μL) over time, overall. (DOCX)

S25 Table Descriptive statistics for pRETIC (percent) over time, overall. (DOCX)

S26 Table Descriptive statistics for ALT (U/L) over time, overall. (DOCX)

S27 Table Descriptive statistics for AST (U/L) over time, overall. (DOCX)

S28 Table Descriptive statistics for ALP (U/L) over time, overall. (DOCX)

S29 Table Descriptive statistics for GGT (U/L) over time, overall. (DOCX)

S30 Table Descriptive statistics over for CRP (mg/L) time, overall. (DOCX)

S31 Table Descriptive statistics for bilirubin (mg/dL) over time, overall. (DOCX)

S32 Table Descriptive statistics for BUN (mg/dL) over time, overall. (DOCX)

S33 Table Descriptive statistics for creatinine (mg/dL) over time, overall. (DOCX)

S34 Table Descriptive statistics for calcium (mg/dL) over time, overall. (DOCX)

S35 Table Descriptive statistics for albumin (g/dL) over time, overall. (DOCX)

S36 Table Descriptive statistics for glucose (mg/dL) over time, overall. (DOCX)

S37 Table Descriptive statistics for serum viral load by plaque assay (PFU/mL) over time, overall. (DOCX)

S38 Table Descriptive statistics for serum viral load by plaque assay (PFU/mL) over time, by exposure dose (PFU). (DOCX)

S39 Table Descriptive statistics for serum viral load by qRT-PCR (GE/mL) over time, overall. (DOCX)

S40 Table Descriptive statistics for serum viral load by qRT-PCR (GE/mL) over time, by exposure dose (PFU). (DOCX)

S41 Table Descriptive statistics for tissue viral load by plaque assay (PFU/g), overall. (DOCX)

S42 Table Descriptive statistics for tissue viral load by plaque assay (PFU/g), by sex. (DOCX)

S43 Table Descriptive statistics for tissue viral load by plaque assay (PFU/g), by animal origin. (DOCX)

S44 Table Descriptive statistics for tissue viral load by qRT-PCR (GE/μg), overall. (DOCX)

S45 Table Descriptive statistics for tissue viral load by qRT-PCR (GE/μg), by sex. (DOCX)

S46 Table Descriptive statistics for tissue viral load by qRT-PCR (GE/μg), by animal origin. (DOCX)
