# Supplementary material for: Natural history of disease in cynomolgus monkeys exposed to Ebola virus Kikwit strain demonstrates the reliability of this non-human primate model for Ebola virus disease
Source: PLoS One. 2021 Jul 2;16(7):e0252874. doi: 10.1371/journal.pone.0252874 (PMC8253449; doi:10.1371/journal.pone.0252874)
Supplement: S1 Table — (DOCX) [file pone.0252874.s001.docx]

### S1 Table. Descriptive Statistics for Weight (kg) over Time, Overall

| Days Post-Exposure | N | Mean | SD | Min | Max | 95% CI |
| --- | --- | --- | --- | --- | --- | --- |
| 0 | 105 | 4.15 | 1.36 | 2.42 | 9.34 | 3.89, 4.41 |
| 1 | 2 | 3.80 | 0.87 | 3.18 | 4.41 | 0, 11.61 |
| 3 | 98 | 4.12 | 1.34 | 2.60 | 9.32 | 3.85, 4.39 |
| 4 | 8 | 4.64 | 1.56 | 2.40 | 7.44 | 3.34, 5.94 |
| 5 | 63 | 3.99 | 1.03 | 2.60 | 6.90 | 3.73, 4.25 |
| 6 | 44 | 4.23 | 1.64 | 2.58 | 9.28 | 3.73, 4.73 |
| 7 | 63 | 4.09 | 1.3 | 2.46 | 8.12 | 3.76, 4.41 |
| 8 | 14 | 3.45 | 0.83 | 2.69 | 6.00 | 2.97, 3.93 |
| 9 | 11 | 4.11 | 1.52 | 2.71 | 7.16 | 3.09, 5.13 |
| 10 | 17 | 4.70 | 1.69 | 2.60 | 9.26 | 3.83, 5.57 |
| 11 | 2 | 4.97 | 0.3 | 4.76 | 5.18 | 2.3, 7.64 |
| 12 | 1 | 5.40 | - - | 5.40 | 5.40 | - -, - - |
| 14 | 4 | 5.46 | 2.94 | 3.16 | 9.38 | 0.79, 10.14 |
| 19 | 1 | 2.98 | - - | 2.98 | 2.98 | - -, - - |
| 21 | 1 | 3.38 | - - | 3.38 | 3.38 | - -, - - |
| T | 71 | 4.09 | 1.37 | 2.46 | 9.38 | 3.76, 4.41 |

### 
