# Supplementary material for: Natural history of disease in cynomolgus monkeys exposed to Ebola virus Kikwit strain demonstrates the reliability of this non-human primate model for Ebola virus disease
Source: PLoS One. 2021 Jul 2;16(7):e0252874. doi: 10.1371/journal.pone.0252874 (PMC8253449; doi:10.1371/journal.pone.0252874)
Supplement: S2 Table — (DOCX) [file pone.0252874.s002.docx]

### S2 Table. Descriptive Statistics for Weight (kg) over Time, by Sex

| Sex | Days Post-Exposure | N | Mean | SD | Min | Max | 95% CI |
| --- | --- | --- | --- | --- | --- | --- | --- |
| Female | 0 | 52 | 3.42 | 0.61 | 2.70 | 5.20 | 3.25, 3.59 |
| Female | 1 | 1 | 3.18 | - - | 3.18 | 3.18 | - -, - - |
| Female | 3 | 51 | 3.43 | 0.6 | 2.60 | 5.20 | 3.26, 3.6 |
| Female | 4 | 1 | 3.18 | - - | 3.18 | 3.18 | - -, - - |
| Female | 5 | 34 | 3.42 | 0.63 | 2.60 | 5.00 | 3.2, 3.64 |
| Female | 6 | 21 | 3.42 | 0.64 | 2.74 | 5.11 | 3.12, 3.71 |
| Female | 7 | 32 | 3.32 | 0.57 | 2.60 | 5.06 | 3.11, 3.52 |
| Female | 8 | 10 | 3.24 | 0.46 | 2.69 | 3.92 | 2.91, 3.57 |
| Female | 9 | 6 | 2.97 | 0.24 | 2.71 | 3.36 | 2.71, 3.22 |
| Female | 10 | 6 | 3.11 | 0.41 | 2.60 | 3.74 | 2.68, 3.54 |
| Female | 14 | 1 | 3.16 | - - | 3.16 | 3.16 | - -, - - |
| Female | 19 | 1 | 2.98 | - - | 2.98 | 2.98 | - -, - - |
| Female | T | 35 | 3.42 | 0.64 | 2.69 | 5.06 | 3.2, 3.64 |
| Male | 0 | 53 | 4.87 | 1.51 | 2.42 | 9.34 | 4.45, 5.28 |
| Male | 1 | 1 | 4.41 | - - | 4.41 | 4.41 | - -, - - |
| Male | 3 | 47 | 4.87 | 1.5 | 2.71 | 9.32 | 4.43, 5.32 |
| Male | 4 | 7 | 4.85 | 1.55 | 2.40 | 7.44 | 3.41, 6.29 |
| Male | 5 | 29 | 4.66 | 1.02 | 2.90 | 6.90 | 4.27, 5.05 |
| Male | 6 | 23 | 4.97 | 1.92 | 2.58 | 9.28 | 4.14, 5.81 |
| Male | 7 | 31 | 4.88 | 1.36 | 2.46 | 8.12 | 4.39, 5.38 |
| Male | 8 | 4 | 3.96 | 1.36 | 3.08 | 6.00 | 1.79, 6.14 |
| Male | 9 | 5 | 5.47 | 1.18 | 4.24 | 7.16 | 4, 6.94 |
| Male | 10 | 11 | 5.57 | 1.47 | 3.19 | 9.26 | 4.58, 6.55 |
| Male | 11 | 2 | 4.97 | 0.3 | 4.76 | 5.18 | 2.3, 7.64 |
| Male | 12 | 1 | 5.40 | - - | 5.40 | 5.40 | - -, - - |
| Male | 14 | 3 | 6.23 | 3.07 | 3.25 | 9.38 | 0, 13.85 |
| Male | 21 | 1 | 3.38 | - - | 3.38 | 3.38 | - -, - - |
| Male | T | 36 | 4.73 | 1.58 | 2.46 | 9.38 | 4.2, 5.27 |

### 
