# Supplementary material for: Natural history of disease in cynomolgus monkeys exposed to Ebola virus Kikwit strain demonstrates the reliability of this non-human primate model for Ebola virus disease
Source: PLoS One. 2021 Jul 2;16(7):e0252874. doi: 10.1371/journal.pone.0252874 (PMC8253449; doi:10.1371/journal.pone.0252874)
Supplement: S3 Table — (DOCX) [file pone.0252874.s003.docx]

### S3 Table. Descriptive Statistics for Weight (kg) over Time, by Age

| Age | Days Post-Exposure | N | Mean | SD | Min | Max | 95% CI |
| --- | --- | --- | --- | --- | --- | --- | --- |
| Adults | 0 | 54 | 4.51 | 1.25 | 2.70 | 8.02 | 4.17, 4.85 |
| Adults | 3 | 49 | 4.41 | 1.23 | 2.78 | 8.03 | 4.06, 4.77 |
| Adults | 4 | 5 | 5.42 | 1.3 | 4.16 | 7.44 | 3.8, 7.03 |
| Adults | 5 | 39 | 4.32 | 1.15 | 2.75 | 6.90 | 3.94, 4.69 |
| Adults | 6 | 18 | 4.49 | 1.34 | 2.80 | 8.01 | 3.82, 5.15 |
| Adults | 7 | 39 | 4.41 | 1.3 | 2.71 | 7.96 | 3.99, 4.83 |
| Adults | 8 | 4 | 3.20 | 0.54 | 2.77 | 3.92 | 2.34, 4.07 |
| Adults | 9 | 7 | 4.50 | 1.75 | 2.71 | 7.16 | 2.89, 6.12 |
| Adults | 10 | 10 | 5.03 | 1.14 | 2.73 | 6.11 | 4.22, 5.85 |
| Adults | 11 | 1 | 5.18 | - - | 5.18 | 5.18 | - -, - - |
| Adults | 12 | 1 | 5.40 | - - | 5.40 | 5.40 | - -, - - |
| Adults | 14 | 2 | 4.65 | 1.99 | 3.25 | 6.06 | 0, 22.51 |
| Adults | 21 | 1 | 3.38 | - - | 3.38 | 3.38 | - -, - - |
| Adults | T | 41 | 4.34 | 1.23 | 2.71 | 7.96 | 3.96, 4.73 |
| Juveniles | 0 | 34 | 3.34 | 0.49 | 2.42 | 4.31 | 3.16, 3.51 |
| Juveniles | 1 | 2 | 3.80 | 0.87 | 3.18 | 4.41 | 0, 11.61 |
| Juveniles | 3 | 32 | 3.40 | 0.48 | 2.60 | 4.43 | 3.23, 3.57 |
| Juveniles | 4 | 3 | 3.35 | 1.05 | 2.40 | 4.48 | 0.74, 5.96 |
| Juveniles | 5 | 24 | 3.46 | 0.46 | 2.60 | 4.40 | 3.27, 3.66 |
| Juveniles | 6 | 11 | 3.14 | 0.37 | 2.58 | 3.59 | 2.89, 3.39 |
| Juveniles | 7 | 21 | 3.35 | 0.58 | 2.46 | 4.52 | 3.09, 3.61 |
| Juveniles | 8 | 7 | 3.16 | 0.32 | 2.69 | 3.60 | 2.86, 3.46 |
| Juveniles | 9 | 4 | 3.41 | 0.73 | 2.80 | 4.45 | 2.24, 4.57 |
| Juveniles | 10 | 4 | 3.36 | 0.76 | 2.60 | 4.40 | 2.15, 4.56 |
| Juveniles | 11 | 1 | 4.76 | - - | 4.76 | 4.76 | - -, - - |
| Juveniles | T | 21 | 3.28 | 0.48 | 2.46 | 4.44 | 3.06, 3.49 |
| Unknown | 0 | 17 | 4.64 | 2.06 | 2.88 | 9.34 | 3.58, 5.7 |
| Unknown | 3 | 17 | 4.64 | 2.06 | 2.94 | 9.32 | 3.58, 5.7 |
| Unknown | 6 | 15 | 4.73 | 2.17 | 2.98 | 9.28 | 3.52, 5.93 |
| Unknown | 7 | 3 | 5.04 | 2.7 | 3.13 | 8.12 | 0, 11.73 |
| Unknown | 8 | 3 | 4.45 | 1.34 | 3.58 | 6.00 | 1.12, 7.79 |
| Unknown | 10 | 3 | 5.39 | 3.37 | 3.16 | 9.26 | 0, 13.75 |
| Unknown | 14 | 2 | 6.27 | 4.4 | 3.16 | 9.38 | 0, 45.79 |
| Unknown | 19 | 1 | 2.98 | - - | 2.98 | 2.98 | - -, - - |
| Unknown | T | 9 | 4.81 | 2.39 | 2.98 | 9.38 | 2.97, 6.65 |
