# Supplementary material for: Natural history of disease in cynomolgus monkeys exposed to Ebola virus Kikwit strain demonstrates the reliability of this non-human primate model for Ebola virus disease
Source: PLoS One. 2021 Jul 2;16(7):e0252874. doi: 10.1371/journal.pone.0252874 (PMC8253449; doi:10.1371/journal.pone.0252874)
Supplement: S4 Table — (DOCX) [file pone.0252874.s004.docx]

### S4 Table. Descriptive Statistics for Temperature (C) over Time, Overall

| Days Post-Exposure | N | Mean | SD | Min | Max | 95% CI |
| --- | --- | --- | --- | --- | --- | --- |
| 0 | 105 | 38.0 | 0.6 | 36.3 | 39.4 | 37.9, 38.1 |
| 1 | 2 | 37.3 | 0.8 | 36.7 | 37.9 | 29.7, 44.9 |
| 3 | 97 | 38.1 | 0.8 | 35.9 | 40.1 | 37.9, 38.2 |
| 4 | 8 | 38.5 | 0.6 | 37.9 | 39.4 | 38, 39 |
| 5 | 63 | 38.6 | 1 | 36.5 | 40.4 | 38.4, 38.9 |
| 6 | 40 | 38.2 | 2.2 | 32.1 | 41.1 | 37.5, 38.9 |
| 7 | 55 | 37.0 | 2.6 | 27.4 | 40.4 | 36.3, 37.7 |
| 8 | 11 | 34.7 | 2.9 | 29.6 | 38.8 | 32.7, 36.6 |
| 9 | 9 | 33.0 | 3.8 | 27.3 | 39.1 | 30, 35.9 |
| 10 | 14 | 36.4 | 2.8 | 30.7 | 39.7 | 34.8, 38 |
| 11 | 2 | 32.8 | 7.6 | 27.4 | 38.2 | 0, 101.2 |
| 12 | 1 | 32.1 | - - | 32.1 | 32.1 | - -, - - |
| 14 | 4 | 38.0 | 0.9 | 36.7 | 38.7 | 36.6, 39.3 |
| 19 | 1 | 32.6 | - - | 32.6 | 32.6 | - -, - - |
| 21 | 1 | 37.4 | - - | 37.4 | 37.4 | - -, - - |
| T | 68 | 35.0 | 3.2 | 27.3 | 40.8 | 34.2, 35.8 |
