# Supplementary material for: Natural history of disease in cynomolgus monkeys exposed to Ebola virus Kikwit strain demonstrates the reliability of this non-human primate model for Ebola virus disease
Source: PLoS One. 2021 Jul 2;16(7):e0252874. doi: 10.1371/journal.pone.0252874 (PMC8253449; doi:10.1371/journal.pone.0252874)
Supplement: S5 Table — (DOCX) [file pone.0252874.s005.docx]

### S5 Table. Descriptive Statistics for Temperature (C) over Time, by Sex

| Sex | Days Post-Exposure | N | Mean | SD | Min | Max | 95% CI |
| --- | --- | --- | --- | --- | --- | --- | --- |
| Female | 0 | 52 | 37.8 | 0.6 | 36.3 | 38.9 | 37.6, 38 |
| Female | 1 | 1 | 37.9 | - - | 37.9 | 37.9 | - -, - - |
| Female | 3 | 51 | 37.9 | 0.8 | 35.9 | 39.7 | 37.7, 38.1 |
| Female | 4 | 1 | 37.9 | - - | 37.9 | 37.9 | - -, - - |
| Female | 5 | 34 | 38.4 | 1 | 36.5 | 40.2 | 38.1, 38.8 |
| Female | 6 | 19 | 38.0 | 1.9 | 32.8 | 40.1 | 37.1, 38.9 |
| Female | 7 | 26 | 36.4 | 3.1 | 27.4 | 40.2 | 35.2, 37.7 |
| Female | 8 | 7 | 33.6 | 3 | 29.6 | 37.1 | 30.8, 36.3 |
| Female | 9 | 5 | 31.4 | 3.9 | 27.3 | 37.8 | 26.6, 36.2 |
| Female | 10 | 6 | 36.2 | 2.5 | 32.2 | 38.5 | 33.5, 38.8 |
| Female | 14 | 1 | 38.3 | - - | 38.3 | 38.3 | - -, - - |
| Female | 19 | 1 | 32.6 | - - | 32.6 | 32.6 | - -, - - |
| Female | T | 33 | 34.2 | 3.3 | 27.3 | 39.6 | 33.1, 35.4 |
| Male | 0 | 53 | 38.2 | 0.5 | 37.2 | 39.4 | 38.1, 38.4 |
| Male | 1 | 1 | 36.7 | - - | 36.7 | 36.7 | - -, - - |
| Male | 3 | 46 | 38.3 | 0.7 | 36.5 | 40.1 | 38.1, 38.5 |
| Male | 4 | 7 | 38.6 | 0.6 | 37.9 | 39.4 | 38, 39.1 |
| Male | 5 | 29 | 38.8 | 0.9 | 37.5 | 40.4 | 38.5, 39.2 |
| Male | 6 | 21 | 38.4 | 2.6 | 32.1 | 41.1 | 37.2, 39.6 |
| Male | 7 | 29 | 37.5 | 1.9 | 32.7 | 40.4 | 36.8, 38.2 |
| Male | 8 | 4 | 36.7 | 1.5 | 35.4 | 38.8 | 34.3, 39 |
| Male | 9 | 4 | 35.0 | 3.1 | 31.8 | 39.1 | 30, 39.9 |
| Male | 10 | 8 | 36.6 | 3.1 | 30.7 | 39.7 | 34, 39.2 |
| Male | 11 | 2 | 32.8 | 7.6 | 27.4 | 38.2 | 0, 101.2 |
| Male | 12 | 1 | 32.1 | - - | 32.1 | 32.1 | - -, - - |
| Male | 14 | 3 | 37.9 | 1 | 36.7 | 38.7 | 35.3, 40.4 |
| Male | 21 | 1 | 37.4 | - - | 37.4 | 37.4 | - -, - - |
| Male | T | 35 | 35.7 | 3 | 27.4 | 40.8 | 34.7, 36.7 |

### 
