# Supplementary material for: Natural history of disease in cynomolgus monkeys exposed to Ebola virus Kikwit strain demonstrates the reliability of this non-human primate model for Ebola virus disease
Source: PLoS One. 2021 Jul 2;16(7):e0252874. doi: 10.1371/journal.pone.0252874 (PMC8253449; doi:10.1371/journal.pone.0252874)
Supplement: S6 Table — (DOCX) [file pone.0252874.s006.docx]

### S6 Table. Descriptive Statistics for PT (seconds) over Time, Overall

| Days Post-Exposure | N | Mean | SD | Min | Max | 95% CI |
| --- | --- | --- | --- | --- | --- | --- |
| 0 | 42 | 18 | 2 | 12 | 22 | 17, 19 |
| 3 | 44 | 18 | 2 | 11 | 23 | 18, 19 |
| 5 | 28 | 19 | 5 | 10 | 33 | 17, 21 |
| 6 | 18 | 26 | 4 | 21 | 33 | 24, 28 |
| 7 | 28 | 28 | 11 | 12 | 63 | 23, 32 |
| 8 | 6 | 28 | 9 | 19 | 42 | 18, 38 |
| 9 | 6 | 33 | 13 | 22 | 57 | 19, 47 |
| 10 | 7 | 25 | 7 | 16 | 37 | 18, 31 |
| 11 | 1 | 10 | - - | 10 | 10 | - -, - - |
| 14 | 2 | 18 | 1 | 18 | 19 | 12, 25 |
| 21 | 1 | 19 | - - | 19 | 19 | - -, - - |
| T | 27 | 34 | 13 | 10 | 63 | 29, 39 |
