# Supplementary material for: Natural history of disease in cynomolgus monkeys exposed to Ebola virus Kikwit strain demonstrates the reliability of this non-human primate model for Ebola virus disease
Source: PLoS One. 2021 Jul 2;16(7):e0252874. doi: 10.1371/journal.pone.0252874 (PMC8253449; doi:10.1371/journal.pone.0252874)
Supplement: S7 Table — (DOCX) [file pone.0252874.s007.docx]

### S7 Table. Descriptive Statistics for PTT (seconds) over Time, Overall

| Days Post-Exposure | N | Mean | SD | Min | Max | 95% CI |
| --- | --- | --- | --- | --- | --- | --- |
| 0 | 62 | 47 | 19 | 21 | 78 | 42, 52 |
| 3 | 65 | 51 | 32 | 20 | 250 | 43, 59 |
| 5 | 48 | 52 | 23 | 20 | 105 | 45, 58 |
| 6 | 19 | 89 | 23 | 45 | 140 | 78, 100 |
| 7 | 38 | 90 | 63 | 21 | 350 | 70, 111 |
| 8 | 5 | 116 | 18 | 100 | 147 | 94, 139 |
| 9 | 6 | 174 | 19 | 154 | 211 | 154, 195 |
| 10 | 9 | 98 | 51 | 22 | 171 | 58, 137 |
| 11 | 1 | 136 | - - | 136 | 136 | - -, - - |
| 14 | 2 | 68 | 4 | 66 | 71 | 37, 100 |
| 21 | 1 | 59 | - - | 59 | 59 | - -, - - |
| T | 37 | 121 | 71 | 27 | 350 | 97, 145 |
