# Supplementary material for: Natural history of disease in cynomolgus monkeys exposed to Ebola virus Kikwit strain demonstrates the reliability of this non-human primate model for Ebola virus disease
Source: PLoS One. 2021 Jul 2;16(7):e0252874. doi: 10.1371/journal.pone.0252874 (PMC8253449; doi:10.1371/journal.pone.0252874)
Supplement: S8 Table — (DOCX) [file pone.0252874.s008.docx]

### S8 Table. Descriptive Statistics for PLT (10^3/µL) over Time, Overall

| Days Post-Exposure | N | Mean | SD | Min | Max | 95% CI |
| --- | --- | --- | --- | --- | --- | --- |
| 0 | 102 | 369 | 103 | 4 | 577 | 349, 389 |
| 1 | 2 | 504 | 6 | 500 | 509 | 447, 562 |
| 3 | 98 | 362 | 113 | 4 | 666 | 339, 385 |
| 4 | 8 | 385 | 113 | 216 | 498 | 291, 479 |
| 5 | 68 | 282 | 121 | 2 | 631 | 253, 311 |
| 6 | 45 | 201 | 76 | 85 | 399 | 178, 223 |
| 7 | 52 | 225 | 104 | 99 | 499 | 196, 254 |
| 8 | 16 | 157 | 73 | 50 | 321 | 118, 196 |
| 9 | 8 | 300 | 478 | 101 | 1481 | 0, 700 |
| 10 | 12 | 282 | 110 | 61 | 435 | 212, 352 |
| 11 | 1 | 32 | - - | 32 | 32 | - -, - - |
| 14 | 4 | 308 | 166 | 148 | 512 | 44, 571 |
| 21 | 1 | 466 | - - | 466 | 466 | - -, - - |
| T | 68 | 170 | 67 | 32 | 324 | 153, 186 |
