# Supplementary material for: Natural history of disease in cynomolgus monkeys exposed to Ebola virus Kikwit strain demonstrates the reliability of this non-human primate model for Ebola virus disease
Source: PLoS One. 2021 Jul 2;16(7):e0252874. doi: 10.1371/journal.pone.0252874 (PMC8253449; doi:10.1371/journal.pone.0252874)
Supplement: S9 Table — (DOCX) [file pone.0252874.s009.docx]

### S9 Table. Descriptive Statistics for MPV (fL) over Time, Overall

| Days Post-Exposure | N | Mean | SD | Min | Max | 95% CI |
| --- | --- | --- | --- | --- | --- | --- |
| 0 | 106 | 9.2 | 1.8 | 0.5 | 15.4 | 8.9, 9.6 |
| 1 | 2 | 8.2 | 0.4 | 8.0 | 8.5 | 5.1, 11.4 |
| 3 | 102 | 8.9 | 1.8 | 0.3 | 14.8 | 8.6, 9.3 |
| 4 | 8 | 9.9 | 2.1 | 8.2 | 14.0 | 8.2, 11.6 |
| 5 | 72 | 9.0 | 2 | 0.2 | 13.4 | 8.5, 9.5 |
| 6 | 44 | 9.0 | 2.1 | 0.1 | 14.3 | 8.3, 9.6 |
| 7 | 55 | 9.6 | 2.1 | 0.1 | 14.5 | 9.1, 10.2 |
| 8 | 17 | 9.5 | 1.4 | 7.2 | 13.5 | 8.8, 10.2 |
| 9 | 9 | 11.4 | 2 | 9.0 | 14.7 | 9.8, 12.9 |
| 10 | 12 | 9.7 | 1.7 | 7.4 | 12.8 | 8.6, 10.7 |
| 11 | 1 | 12.4 | - - | 12.4 | 12.4 | - -, - - |
| 14 | 4 | 10.3 | 1 | 8.9 | 11.0 | 8.8, 11.9 |
| 21 | 1 | 9.0 | - - | 9.0 | 9.0 | - -, - - |
| T | 69 | 10.0 | 2.6 | 0.1 | 14.5 | 9.4, 10.6 |
