# Supplementary material for: Natural history of disease in cynomolgus monkeys exposed to Ebola virus Kikwit strain demonstrates the reliability of this non-human primate model for Ebola virus disease
Source: PLoS One. 2021 Jul 2;16(7):e0252874. doi: 10.1371/journal.pone.0252874 (PMC8253449; doi:10.1371/journal.pone.0252874)
Supplement: S10 Table — (DOCX) [file pone.0252874.s010.docx]

### S10 Table. Descriptive Statistics for White Blood Cell Count (10^3/µL) over Time, Overall

| Days Post-Exposure | N | Mean | SD | Min | Max | 95% CI |
| --- | --- | --- | --- | --- | --- | --- |
| 0 | 106 | 8.94 | 3.32 | 3.54 | 17.27 | 8.3, 9.58 |
| 1 | 2 | 6.30 | 2.47 | 4.55 | 8.05 | 0, 28.54 |
| 3 | 102 | 9.25 | 3.54 | 3.62 | 22.33 | 8.56, 9.95 |
| 4 | 8 | 11.22 | 2.43 | 7.63 | 14.40 | 9.18, 13.25 |
| 5 | 72 | 15.35 | 8.52 | 4.46 | 50.08 | 13.35, 17.35 |
| 6 | 45 | 17.09 | 6.86 | 4.77 | 32.32 | 15.03, 19.15 |
| 7 | 56 | 16.14 | 9.14 | 4.88 | 43.09 | 13.69, 18.59 |
| 8 | 17 | 16.49 | 6.11 | 2.98 | 28.10 | 13.35, 19.63 |
| 9 | 9 | 18.20 | 7.77 | 5.89 | 32.36 | 12.23, 24.17 |
| 10 | 12 | 12.05 | 5.9 | 5.23 | 20.79 | 8.3, 15.8 |
| 11 | 1 | 3.42 | - - | 3.42 | 3.42 | - -, - - |
| 14 | 4 | 11.73 | 5.41 | 6.39 | 18.10 | 3.12, 20.34 |
| 21 | 1 | 7.27 | - - | 7.27 | 7.27 | - -, - - |
| T | 70 | 19.29 | 8.88 | 2.98 | 43.09 | 17.17, 21.41 |
