# Supplementary material for: Natural history of disease in cynomolgus monkeys exposed to Ebola virus Kikwit strain demonstrates the reliability of this non-human primate model for Ebola virus disease
Source: PLoS One. 2021 Jul 2;16(7):e0252874. doi: 10.1371/journal.pone.0252874 (PMC8253449; doi:10.1371/journal.pone.0252874)
Supplement: S11 Table — (DOCX) [file pone.0252874.s011.docx]

### S11 Table. Descriptive Statistics for cLYMPH (10^3/µL) over Time, Overall

| Days Post-Exposure | N | Mean | SD | Min | Max | 95% CI |
| --- | --- | --- | --- | --- | --- | --- |
| 0 | 106 | 3.82 | 1.72 | 1.56 | 10.27 | 3.49, 4.15 |
| 1 | 2 | 1.61 | 0.25 | 1.43 | 1.79 | 0, 3.9 |
| 3 | 102 | 3.27 | 1.33 | 1.33 | 8.19 | 3.01, 3.53 |
| 4 | 8 | 2.53 | 1.56 | 1.38 | 5.36 | 1.23, 3.83 |
| 5 | 72 | 2.70 | 1.86 | 0.54 | 12.70 | 2.26, 3.13 |
| 6 | 45 | 3.57 | 2.68 | 0.50 | 13.30 | 2.76, 4.37 |
| 7 | 56 | 3.71 | 2.61 | 0.60 | 15.00 | 3.01, 4.41 |
| 8 | 17 | 4.26 | 2.7 | 1.10 | 9.90 | 2.88, 5.65 |
| 9 | 9 | 4.39 | 2.04 | 1.90 | 8.35 | 2.82, 5.95 |
| 10 | 12 | 2.83 | 1.44 | 1.24 | 5.98 | 1.91, 3.75 |
| 11 | 1 | 2.06 | - - | 2.06 | 2.06 | - -, - - |
| 14 | 4 | 3.95 | 1.37 | 2.47 | 5.20 | 1.77, 6.13 |
| 21 | 1 | 5.94 | - - | 5.94 | 5.94 | - -, - - |
| T | 70 | 4.47 | 2.48 | 0.73 | 12.70 | 3.88, 5.06 |
