# Supplementary material for: Natural history of disease in cynomolgus monkeys exposed to Ebola virus Kikwit strain demonstrates the reliability of this non-human primate model for Ebola virus disease
Source: PLoS One. 2021 Jul 2;16(7):e0252874. doi: 10.1371/journal.pone.0252874 (PMC8253449; doi:10.1371/journal.pone.0252874)
Supplement: S12 Table — (DOCX) [file pone.0252874.s012.docx]

### S12 Table. Descriptive Statistics for pLYMPH (Percent) over Time, Overall

| Days Post-Exposure | N | Mean | SD | Min | Max | 95% CI |
| --- | --- | --- | --- | --- | --- | --- |
| 0 | 98 | 45.6 | 15.1 | 10.0 | 82.0 | 42.6, 48.6 |
| 1 | 2 | 28.5 | 15.3 | 17.7 | 39.3 | 0, 165.7 |
| 3 | 94 | 38.3 | 14.7 | 11.7 | 83.0 | 35.3, 41.3 |
| 4 | 8 | 22.4 | 11.7 | 12.2 | 43.0 | 12.7, 32.2 |
| 5 | 68 | 22.9 | 17.2 | 2.7 | 82.0 | 18.8, 27.1 |
| 6 | 41 | 20.7 | 12.7 | 3.1 | 53.7 | 16.7, 24.6 |
| 7 | 52 | 26.6 | 16 | 4.5 | 79.2 | 22.2, 31.1 |
| 8 | 16 | 30.4 | 18.7 | 6.3 | 78.5 | 20.4, 40.4 |
| 9 | 8 | 28.6 | 12.9 | 15.7 | 53.5 | 17.8, 39.4 |
| 10 | 10 | 29.8 | 21.7 | 6.0 | 72.5 | 14.2, 45.3 |
| 11 | 1 | 60.2 | - - | 60.2 | 60.2 | - -, - - |
| 14 | 2 | 48.0 | 43.3 | 17.4 | 78.6 | 0, 436.8 |
| 21 | 1 | 81.7 | - - | 81.7 | 81.7 | - -, - - |
| T | 67 | 25.9 | 13.1 | 6.3 | 78.5 | 22.7, 29.1 |
