# Supplementary material for: Natural history of disease in cynomolgus monkeys exposed to Ebola virus Kikwit strain demonstrates the reliability of this non-human primate model for Ebola virus disease
Source: PLoS One. 2021 Jul 2;16(7):e0252874. doi: 10.1371/journal.pone.0252874 (PMC8253449; doi:10.1371/journal.pone.0252874)
Supplement: S13 Table — (DOCX) [file pone.0252874.s013.docx]

### S13 Table. Descriptive Statistics for cNEUT (10^3/µL) over Time, Overall

| Days Post-Exposure | N | Mean | SD | Min | Max | 95% CI |
| --- | --- | --- | --- | --- | --- | --- |
| 0 | 61 | 4.73 | 2.84 | 0.81 | 13.04 | 4, 5.45 |
| 1 | 2 | 4.34 | 2.76 | 2.39 | 6.30 | 0, 29.19 |
| 3 | 60 | 4.74 | 2.67 | 0.61 | 11.32 | 4.05, 5.43 |
| 4 | 2 | 9.59 | 0.06 | 9.55 | 9.63 | - -*, - -* |
| 5 | 61 | 11.41 | 8.63 | 0.93 | 44.86 | 9.2, 13.62 |
| 6 | 12 | 16.43 | 5.86 | 2.94 | 24.92 | 12.71, 20.16 |
| 7 | 38 | 10.58 | 7.49 | 0.81 | 35.92 | 8.12, 13.04 |
| 8 | 6 | 11.12 | 6.1 | 0.39 | 17.68 | 4.72, 17.51 |
| 9 | 6 | 13.11 | 4.23 | 8.42 | 20.62 | 8.67, 17.54 |
| 10 | 10 | 8.57 | 7.31 | 1.69 | 19.17 | 3.35, 13.8 |
| 11 | 1 | 0.50 | - - | 0.50 | 0.50 | - -, - - |
| 14 | 2 | 5.84 | 7.21 | 0.74 | 10.94 | 0, 70.64 |
| 21 | 1 | 0.70 | - - | 0.70 | 0.70 | - -, - - |
| T | 44 | 14.12 | 7.75 | 0.39 | 35.92 | 11.76, 16.47 |
