# Supplementary material for: Natural history of disease in cynomolgus monkeys exposed to Ebola virus Kikwit strain demonstrates the reliability of this non-human primate model for Ebola virus disease
Source: PLoS One. 2021 Jul 2;16(7):e0252874. doi: 10.1371/journal.pone.0252874 (PMC8253449; doi:10.1371/journal.pone.0252874)
Supplement: S14 Table — (DOCX) [file pone.0252874.s014.docx]

### S14 Table. Descriptive Statistics for pNEUT (Percent) over Time, Overall

| Days Post-Exposure | N | Mean | SD | Min | Max | 95% CI |
| --- | --- | --- | --- | --- | --- | --- |
| 0 | 59 | 49.7 | 15.5 | 11.4 | 77.0 | 45.7, 53.8 |
| 1 | 2 | 65.4 | 18.1 | 52.6 | 78.2 | 0, 228 |
| 3 | 58 | 53.0 | 17.1 | 9.5 | 83.0 | 48.6, 57.5 |
| 4 | 2 | 82.2 | 3.7 | 79.6 | 84.9 | 48.6, 115.9 |
| 5 | 61 | 71.1 | 19.5 | 12.4 | 95.6 | 66.1, 76.1 |
| 6 | 10 | 75.9 | 11 | 56.4 | 94.8 | 68, 83.7 |
| 7 | 38 | 68.8 | 18.6 | 12.9 | 92.9 | 62.7, 74.9 |
| 8 | 6 | 62.4 | 26.7 | 13.2 | 83.4 | 34.4, 90.4 |
| 9 | 5 | 64.1 | 19.1 | 36.5 | 82.3 | 40.4, 87.7 |
| 10 | 10 | 61.5 | 26.6 | 20.4 | 92.2 | 42.4, 80.5 |
| 11 | 1 | 14.6 | - - | 14.6 | 14.6 | - -, - - |
| 14 | 2 | 44.2 | 46.1 | 11.6 | 76.8 | 0, 458.4 |
| 21 | 1 | 9.7 | - - | 9.7 | 9.7 | - -, - - |
| T | 44 | 67.8 | 17.3 | 13.2 | 88.7 | 62.6, 73.1 |
