# Supplementary material for: Natural history of disease in cynomolgus monkeys exposed to Ebola virus Kikwit strain demonstrates the reliability of this non-human primate model for Ebola virus disease
Source: PLoS One. 2021 Jul 2;16(7):e0252874. doi: 10.1371/journal.pone.0252874 (PMC8253449; doi:10.1371/journal.pone.0252874)
Supplement: S15 Table — (DOCX) [file pone.0252874.s015.docx]

### S15 Table. Descriptive Statistics for cMONO (10^3/µL) over Time, Overall

| Days Post-Exposure | N | Mean | SD | Min | Max | 95% CI |
| --- | --- | --- | --- | --- | --- | --- |
| 0 | 104 | 0.51 | 0.41 | 0.05 | 2.60 | 0.43, 0.59 |
| 1 | 2 | 0.16 | 0.1 | 0.09 | 0.23 | 0, 1.05 |
| 3 | 102 | 0.62 | 0.62 | 0.03 | 3.80 | 0.5, 0.74 |
| 4 | 6 | 0.50 | 0.28 | 0.23 | 0.86 | 0.21, 0.8 |
| 5 | 72 | 0.56 | 0.45 | 0.07 | 2.90 | 0.46, 0.67 |
| 6 | 45 | 0.66 | 0.67 | 0.04 | 3.43 | 0.46, 0.86 |
| 7 | 54 | 1.04 | 2.15 | 0.02 | 14.00 | 0.46, 1.63 |
| 8 | 16 | 1.03 | 1.71 | 0.03 | 5.85 | 0.12, 1.95 |
| 9 | 9 | 2.10 | 3.91 | 0.07 | 12.01 | 0, 5.11 |
| 10 | 12 | 0.78 | 0.87 | 0.04 | 2.41 | 0.23, 1.34 |
| 11 | 1 | 0.78 | - - | 0.78 | 0.78 | - -, - - |
| 14 | 4 | 1.14 | 0.75 | 0.52 | 2.20 | 0, 2.33 |
| 21 | 1 | 0.57 | - - | 0.57 | 0.57 | - -, - - |
| T | 68 | 1.38 | 2.48 | 0.03 | 14.00 | 0.78, 1.97 |
