# Supplementary material for: Natural history of disease in cynomolgus monkeys exposed to Ebola virus Kikwit strain demonstrates the reliability of this non-human primate model for Ebola virus disease
Source: PLoS One. 2021 Jul 2;16(7):e0252874. doi: 10.1371/journal.pone.0252874 (PMC8253449; doi:10.1371/journal.pone.0252874)
Supplement: S16 Table — (DOCX) [file pone.0252874.s016.docx]

### S16 Table. Descriptive Statistics for pMONO (Percent) over Time, Overall

| Days Post-Exposure | N | Mean | SD | Min | Max | 95% CI |
| --- | --- | --- | --- | --- | --- | --- |
| 0 | 96 | 5.5 | 3.8 | 0.9 | 25.7 | 4.7, 6.3 |
| 1 | 2 | 2.3 | 0.6 | 1.9 | 2.8 | 0, 8.1 |
| 3 | 94 | 6.5 | 5.5 | 0.5 | 31.0 | 5.4, 7.6 |
| 4 | 6 | 4.8 | 3.1 | 2.0 | 10.3 | 1.6, 8.1 |
| 5 | 68 | 4.1 | 2.9 | 0.7 | 11.8 | 3.4, 4.8 |
| 6 | 40 | 3.6 | 3.3 | 0.2 | 15.7 | 2.5, 4.6 |
| 7 | 51 | 5.6 | 6 | 0.2 | 32.5 | 3.9, 7.3 |
| 8 | 15 | 6.4 | 9.1 | 0.2 | 29.9 | 1.3, 11.4 |
| 9 | 8 | 8.4 | 12.6 | 0.4 | 37.1 | 0, 19 |
| 10 | 10 | 8.1 | 11.8 | 0.4 | 34.2 | 0, 16.6 |
| 11 | 1 | 22.8 | - - | 22.8 | 22.8 | - -, - - |
| 14 | 2 | 6.7 | 2.1 | 5.2 | 8.1 | 0, 25.1 |
| 21 | 1 | 7.8 | - - | 7.8 | 7.8 | - -, - - |
| T | 65 | 6.7 | 8.6 | 0.2 | 37.1 | 4.6, 8.9 |
