# Supplementary material for: Natural history of disease in cynomolgus monkeys exposed to Ebola virus Kikwit strain demonstrates the reliability of this non-human primate model for Ebola virus disease
Source: PLoS One. 2021 Jul 2;16(7):e0252874. doi: 10.1371/journal.pone.0252874 (PMC8253449; doi:10.1371/journal.pone.0252874)
Supplement: S18 Table — (DOCX) [file pone.0252874.s018.docx]

### S18 Table. Descriptive Statistics for pEOS (Percent) over Time, Overall

| Days Post-Exposure | N | Mean | SD | Min | Max | 95% CI |
| --- | --- | --- | --- | --- | --- | --- |
| 0 | 61 | 1.6 | 1.6 | 0.0 | 9.0 | 1.2, 2 |
| 1 | 2 | 2.9 | 3.7 | 0.3 | 5.5 | 0, 35.9 |
| 3 | 60 | 1.6 | 1.6 | 0.1 | 8.2 | 1.2, 2 |
| 4 | 2 | 1.3 | 1.6 | 0.2 | 2.4 | 0, 15.3 |
| 5 | 61 | 0.5 | 0.6 | 0.1 | 2.9 | 0.4, 0.7 |
| 6 | 12 | 0.4 | 0.3 | 0.1 | 1.1 | 0.2, 0.6 |
| 7 | 38 | 0.5 | 0.8 | 0.0 | 3.3 | 0.2, 0.8 |
| 8 | 6 | 0.5 | 0.9 | 0.0 | 2.3 | 0, 1.4 |
| 9 | 6 | 0.4 | 0.3 | 0.1 | 1.0 | 0.1, 0.8 |
| 10 | 10 | 1.0 | 1.3 | 0.1 | 4.6 | 0, 1.9 |
| 11 | 1 | 1.2 | - - | 1.2 | 1.2 | - -, - - |
| 14 | 2 | 1.1 | 0.8 | 0.5 | 1.7 | 0, 8.7 |
| 21 | 1 | 0.7 | - - | 0.7 | 0.7 | - -, - - |
| T | 44 | 0.4 | 0.5 | 0.0 | 2.3 | 0.2, 0.5 |
