# Supplementary material for: Natural history of disease in cynomolgus monkeys exposed to Ebola virus Kikwit strain demonstrates the reliability of this non-human primate model for Ebola virus disease
Source: PLoS One. 2021 Jul 2;16(7):e0252874. doi: 10.1371/journal.pone.0252874 (PMC8253449; doi:10.1371/journal.pone.0252874)
Supplement: S19 Table — (DOCX) [file pone.0252874.s019.docx]

### S19 Table. Descriptive Statistics for cBASO (10^3/µL) over Time, Overall

| Days Post-Exposure | N | Mean | SD | Min | Max | 95% CI |
| --- | --- | --- | --- | --- | --- | --- |
| 0 | 61 | 0.02 | 0.01 | 0.00 | 0.05 | 0.01, 0.02 |
| 1 | 2 | 0.02 | 0.02 | 0.01 | 0.04 | 0, 0.22 |
| 3 | 60 | 0.01 | 0.01 | 0.00 | 0.05 | 0.01, 0.02 |
| 4 | 2 | 0.02 | 0.01 | 0.02 | 0.03 | 0, 0.09 |
| 5 | 61 | 0.07 | 0.15 | 0.00 | 1.15 | 0.03, 0.11 |
| 6 | 12 | 0.16 | 0.11 | 0.00 | 0.35 | 0.09, 0.23 |
| 7 | 38 | 0.10 | 0.19 | 0.00 | 1.04 | 0.04, 0.16 |
| 8 | 6 | 0.11 | 0.08 | 0.03 | 0.21 | 0.03, 0.19 |
| 9 | 6 | 0.15 | 0.05 | 0.06 | 0.19 | 0.1, 0.2 |
| 10 | 10 | 0.06 | 0.08 | 0.00 | 0.21 | 0.01, 0.12 |
| 11 | 1 | 0.04 | - - | 0.04 | 0.04 | - -, - - |
| 14 | 2 | 0.01 | 0 | 0.00 | 0.01 | 0, 0.04 |
| 21 | 1 | 0.01 | - - | 0.01 | 0.01 | - -, - - |
| T | 44 | 0.20 | 0.22 | 0.03 | 1.15 | 0.14, 0.27 |
