# Supplementary material for: Natural history of disease in cynomolgus monkeys exposed to Ebola virus Kikwit strain demonstrates the reliability of this non-human primate model for Ebola virus disease
Source: PLoS One. 2021 Jul 2;16(7):e0252874. doi: 10.1371/journal.pone.0252874 (PMC8253449; doi:10.1371/journal.pone.0252874)
Supplement: S21 Table — (DOCX) [file pone.0252874.s021.docx]

### S21 Table. Descriptive Statistics for Red Blood Cell Count (10^6/µL) over Time, Overall

| Days Post-Exposure | N | Mean | SD | Min | Max | 95% CI |
| --- | --- | --- | --- | --- | --- | --- |
| 0 | 106 | 5.46 | 0.48 | 4.25 | 6.60 | 5.36, 5.55 |
| 1 | 2 | 4.90 | 0.39 | 4.63 | 5.18 | 1.41, 8.4 |
| 3 | 102 | 5.37 | 0.55 | 4.10 | 6.71 | 5.26, 5.48 |
| 4 | 8 | 4.93 | 0.32 | 4.31 | 5.28 | 4.66, 5.2 |
| 5 | 72 | 5.17 | 0.63 | 3.60 | 6.80 | 5.02, 5.32 |
| 6 | 45 | 4.78 | 0.88 | 2.88 | 9.10 | 4.51, 5.04 |
| 7 | 56 | 4.91 | 0.89 | 3.72 | 9.13 | 4.67, 5.15 |
| 8 | 17 | 4.27 | 0.77 | 2.14 | 5.23 | 3.87, 4.66 |
| 9 | 9 | 4.45 | 0.86 | 2.38 | 5.15 | 3.79, 5.11 |
| 10 | 12 | 4.81 | 0.55 | 3.49 | 5.40 | 4.45, 5.16 |
| 11 | 1 | 5.25 | - - | 5.25 | 5.25 | - -, - - |
| 14 | 4 | 4.99 | 0.47 | 4.41 | 5.42 | 4.25, 5.74 |
| 21 | 1 | 5.37 | - - | 5.37 | 5.37 | - -, - - |
| T | 70 | 4.84 | 1.1 | 2.14 | 9.13 | 4.58, 5.1 |
