# Supplementary material for: Natural history of disease in cynomolgus monkeys exposed to Ebola virus Kikwit strain demonstrates the reliability of this non-human primate model for Ebola virus disease
Source: PLoS One. 2021 Jul 2;16(7):e0252874. doi: 10.1371/journal.pone.0252874 (PMC8253449; doi:10.1371/journal.pone.0252874)
Supplement: S22 Table — (DOCX) [file pone.0252874.s022.docx]

### S22 Table. Descriptive Statistics for Hemoglobin (g/dL) over Time, Overall

| Days Post-Exposure | N | Mean | SD | Min | Max | 95% CI |
| --- | --- | --- | --- | --- | --- | --- |
| 0 | 104 | 12.0 | 1 | 8.9 | 14.1 | 11.8, 12.2 |
| 1 | 2 | 11.9 | 1.7 | 10.7 | 13.1 | 0, 27.1 |
| 3 | 102 | 11.9 | 1.1 | 8.8 | 14.5 | 11.7, 12.1 |
| 4 | 8 | 11.8 | 0.9 | 10.7 | 13.2 | 11, 12.6 |
| 5 | 70 | 11.4 | 1.3 | 7.0 | 14.8 | 11.1, 11.7 |
| 6 | 45 | 10.8 | 1.8 | 6.8 | 19.0 | 10.2, 11.3 |
| 7 | 55 | 11.0 | 2 | 6.9 | 20.4 | 10.4, 11.5 |
| 8 | 17 | 10.0 | 2 | 5.2 | 12.4 | 8.9, 11 |
| 9 | 9 | 10.7 | 0.9 | 9.3 | 12.4 | 10, 11.4 |
| 10 | 12 | 10.7 | 0.9 | 8.7 | 11.7 | 10.2, 11.3 |
| 11 | 1 | 12.4 | - - | 12.4 | 12.4 | - -, - - |
| 14 | 4 | 11.4 | 1 | 10.0 | 12.3 | 9.8, 13 |
| 21 | 1 | 11.6 | - - | 11.6 | 11.6 | - -, - - |
| T | 68 | 10.8 | 2.4 | 5.2 | 20.4 | 10.2, 11.4 |
