# Supplementary material for: Natural history of disease in cynomolgus monkeys exposed to Ebola virus Kikwit strain demonstrates the reliability of this non-human primate model for Ebola virus disease
Source: PLoS One. 2021 Jul 2;16(7):e0252874. doi: 10.1371/journal.pone.0252874 (PMC8253449; doi:10.1371/journal.pone.0252874)
Supplement: S23 Table — (DOCX) [file pone.0252874.s023.docx]

### S23 Table. Descriptive Statistics for Hematocrit (Percent) over Time, Overall

| Days Post-Exposure | N | Mean | SD | Min | Max | 95% CI |
| --- | --- | --- | --- | --- | --- | --- |
| 0 | 106 | 38.8 | 2.8 | 30.7 | 45.5 | 38.3, 39.4 |
| 1 | 2 | 37.7 | 4.2 | 34.7 | 40.7 | 0, 75.8 |
| 3 | 102 | 38.3 | 3.1 | 29.4 | 45.8 | 37.7, 38.9 |
| 4 | 8 | 36.7 | 2.7 | 32.7 | 41.7 | 34.4, 39 |
| 5 | 72 | 36.5 | 3.7 | 24.1 | 43.5 | 35.6, 37.4 |
| 6 | 45 | 33.7 | 6 | 22.2 | 65.9 | 31.9, 35.5 |
| 7 | 56 | 35.2 | 6.7 | 23.6 | 63.8 | 33.4, 37 |
| 8 | 17 | 30.9 | 6 | 17.0 | 37.2 | 27.8, 34 |
| 9 | 9 | 31.2 | 6.2 | 16.7 | 36.9 | 26.5, 35.9 |
| 10 | 12 | 34.4 | 3.3 | 27.1 | 39.3 | 32.3, 36.5 |
| 11 | 1 | 39.6 | - - | 39.6 | 39.6 | - -, - - |
| 14 | 4 | 36.7 | 3.9 | 31.5 | 40.6 | 30.5, 42.9 |
| 21 | 1 | 39.3 | - - | 39.3 | 39.3 | - -, - - |
| T | 70 | 34.1 | 8 | 17.0 | 65.9 | 32.2, 36 |
