# Supplementary material for: Natural history of disease in cynomolgus monkeys exposed to Ebola virus Kikwit strain demonstrates the reliability of this non-human primate model for Ebola virus disease
Source: PLoS One. 2021 Jul 2;16(7):e0252874. doi: 10.1371/journal.pone.0252874 (PMC8253449; doi:10.1371/journal.pone.0252874)
Supplement: S24 Table — (DOCX) [file pone.0252874.s024.docx]

### S24 Table. Descriptive Statistics for cRETIC (10^3/µL) over Time, Overall

| Days Post-Exposure | N | Mean | SD | Min | Max | 95% CI |
| --- | --- | --- | --- | --- | --- | --- |
| 0 | 39 | 40.22 | 28 | 8.0 | 143.6 | 31.14, 49.3 |
| 1 | 2 | 70.40 | 13.01 | 61.2 | 79.6 | 0, 187.3 |
| 3 | 39 | 38.75 | 21.77 | 4.2 | 99.4 | 31.69, 45.81 |
| 4 | 2 | 80.75 | 46.32 | 48.0 | 113.5 | 0, 496.88 |
| 5 | 37 | 40.52 | 25.07 | 5.2 | 111.9 | 32.17, 48.88 |
| 6 | 6 | 44.28 | 14.94 | 21.3 | 63.2 | 28.61, 59.96 |
| 7 | 29 | 36.11 | 25.86 | 7.9 | 112.1 | 26.27, 45.94 |
| 8 | 6 | 20.92 | 9.41 | 9.4 | 36.1 | 11.04, 30.79 |
| 9 | 6 | 14.05 | 15.15 | 6.0 | 44.9 | 0, 29.95 |
| 10 | 10 | 37.14 | 42.84 | 4.0 | 143.6 | 6.49, 67.79 |
| 11 | 1 | 4.70 | - - | 4.7 | 4.7 | - -, - - |
| 14 | 2 | 138.70 | 58.12 | 97.6 | 179.8 | 0, 660.93 |
| 21 | 1 | 205.10 | - - | 205.1 | 205.1 | - -, - - |
| T | 25 | 21.26 | 13.04 | 4.7 | 49.1 | 15.87, 26.64 |

### 
