# Supplementary material for: Natural history of disease in cynomolgus monkeys exposed to Ebola virus Kikwit strain demonstrates the reliability of this non-human primate model for Ebola virus disease
Source: PLoS One. 2021 Jul 2;16(7):e0252874. doi: 10.1371/journal.pone.0252874 (PMC8253449; doi:10.1371/journal.pone.0252874)
Supplement: S25 Table — (DOCX) [file pone.0252874.s025.docx]

### S25 Table. Descriptive Statistics for pRETIC (Percent) over Time, Overall

| Days Post-Exposure | N | Mean | SD | Min | Max | 95% CI |
| --- | --- | --- | --- | --- | --- | --- |
| 0 | 58 | 0.78 | 0.48 | 0.15 | 2.61 | 0.65, 0.91 |
| 1 | 2 | 1.43 | 0.16 | 1.32 | 1.54 | 0.03, 2.83 |
| 3 | 60 | 0.79 | 0.47 | 0.08 | 2.21 | 0.67, 0.91 |
| 4 | 2 | 1.60 | 0.78 | 1.04 | 2.15 | 0, 8.65 |
| 5 | 59 | 0.78 | 0.53 | 0.08 | 2.90 | 0.64, 0.92 |
| 6 | 12 | 0.90 | 0.34 | 0.39 | 1.40 | 0.68, 1.11 |
| 7 | 37 | 0.74 | 0.53 | 0.15 | 2.24 | 0.57, 0.92 |
| 8 | 6 | 0.44 | 0.19 | 0.20 | 0.74 | 0.24, 0.65 |
| 9 | 6 | 0.32 | 0.38 | 0.12 | 1.09 | 0, 0.72 |
| 10 | 10 | 0.74 | 0.8 | 0.09 | 2.71 | 0.17, 1.31 |
| 11 | 1 | 0.09 | - - | 0.09 | 0.09 | - -, - - |
| 14 | 2 | 2.59 | 1.12 | 1.80 | 3.38 | 0, 12.63 |
| 21 | 1 | 3.82 | - - | 3.82 | 3.82 | - -, - - |
| T | 42 | 0.58 | 0.37 | 0.09 | 1.94 | 0.46, 0.69 |
