# Supplementary material for: Natural history of disease in cynomolgus monkeys exposed to Ebola virus Kikwit strain demonstrates the reliability of this non-human primate model for Ebola virus disease
Source: PLoS One. 2021 Jul 2;16(7):e0252874. doi: 10.1371/journal.pone.0252874 (PMC8253449; doi:10.1371/journal.pone.0252874)
Supplement: S26 Table — (DOCX) [file pone.0252874.s026.docx]

### S26 Table. Descriptive Statistics for ALT (U/L) over Time, Overall

| Days Post-Exposure | N | Geometric Mean | Geometric CV(%) | Min | Max | 95% CI |
| --- | --- | --- | --- | --- | --- | --- |
| 0 | 104 | 37 | 44 | 19 | 452 | 34, 40 |
| 1 | 2 | 57 | 92 | 33 | 100 | 0, 65794 |
| 3 | 99 | 37 | 38 | 19 | 170 | 35, 40 |
| 4 | 8 | 46 | 68 | 25 | 151 | 27, 76 |
| 5 | 71 | 60 | 87 | 22 | 557 | 51, 72 |
| 6 | 42 | 221 | 172 | 31 | 2000 | 153, 319 |
| 7 | 54 | 178 | 187 | 28 | 2000 | 127, 249 |
| 8 | 13 | 392 | 118 | 66 | 999 | 223, 688 |
| 9 | 7 | 518 | 72 | 188 | 1608 | 285, 943 |
| 10 | 12 | 118 | 304 | 29 | 1649 | 45, 310 |
| 11 | 1 | 660 | - - | 660 | 660 | - -, - - |
| 14 | 4 | 52 | 70 | 31 | 127 | 19, 140 |
| 21 | 1 | 37 | - - | 37 | 37 | - -, - - |
| T | 64 | 429 | 103 | 39 | 2000 | 347, 530 |

### 
