# Supplementary material for: Natural history of disease in cynomolgus monkeys exposed to Ebola virus Kikwit strain demonstrates the reliability of this non-human primate model for Ebola virus disease
Source: PLoS One. 2021 Jul 2;16(7):e0252874. doi: 10.1371/journal.pone.0252874 (PMC8253449; doi:10.1371/journal.pone.0252874)
Supplement: S27 Table — (DOCX) [file pone.0252874.s027.docx]

### S27 Table. Descriptive Statistics for AST (U/L) over Time, Overall

| Days Post-Exposure | N | Mean | SD | Min | Max | 95% CI |
| --- | --- | --- | --- | --- | --- | --- |
| 0 | 50 | 37 | 15 | 22 | 110 | 32, 41 |
| 1 | 2 | 46 | 13 | 37 | 55 | 0, 160 |
| 3 | 50 | 35 | 9 | 23 | 63 | 32, 37 |
| 4 | 4 | 51 | 17 | 33 | 67 | 25, 77 |
| 5 | 39 | 439 | 446 | 32 | 1522 | 294, 583 |
| 6 | 16 | 781 | 752 | 34 | 2410 | 380, 1181 |
| 7 | 20 | 1297 | 810 | 28 | 2734 | 918, 1676 |
| 8 | 3 | 1480 | 459 | 958 | 1821 | 339, 2621 |
| 10 | 5 | 96 | 130 | 29 | 328 | 0, 257 |
| 14 | 2 | 240 | 293 | 32 | 447 | 0, 2876 |
| T | 31 | 1406 | 631 | 418 | 2734 | 1175, 1638 |
