# Supplementary material for: Natural history of disease in cynomolgus monkeys exposed to Ebola virus Kikwit strain demonstrates the reliability of this non-human primate model for Ebola virus disease
Source: PLoS One. 2021 Jul 2;16(7):e0252874. doi: 10.1371/journal.pone.0252874 (PMC8253449; doi:10.1371/journal.pone.0252874)
Supplement: S28 Table — (DOCX) [file pone.0252874.s028.docx]

### S28 Table. Descriptive Statistics for ALP (U/L) over Time, Overall

| Days Post-Exposure | N | Geometric Mean | Geometric CV(%) | Min | Max | 95% CI |
| --- | --- | --- | --- | --- | --- | --- |
| 0 | 104 | 148 | 95 | 11 | 779 | 127, 173 |
| 1 | 2 | 143 | 30 | 116 | 176 | 10, 2020 |
| 3 | 99 | 164 | 79 | 16 | 885 | 143, 189 |
| 4 | 8 | 158 | 71 | 55 | 357 | 93, 269 |
| 5 | 71 | 247 | 139 | 14 | 1938 | 193, 316 |
| 6 | 43 | 483 | 91 | 76 | 1963 | 380, 614 |
| 7 | 56 | 448 | 131 | 14 | 2376 | 343, 585 |
| 8 | 17 | 727 | 81 | 131 | 1712 | 504, 1047 |
| 9 | 8 | 956 | 65 | 257 | 1974 | 581, 1570 |
| 10 | 12 | 299 | 145 | 83 | 2250 | 152, 588 |
| 11 | 1 | 559 | - - | 559 | 559 | - -, - - |
| 14 | 4 | 175 | 141 | 94 | 839 | 33, 929 |
| 21 | 1 | 44 | - - | 44 | 44 | - -, - - |
| T | 70 | 856 | 62 | 131 | 2250 | 747, 981 |

### 
