# Supplementary material for: Natural history of disease in cynomolgus monkeys exposed to Ebola virus Kikwit strain demonstrates the reliability of this non-human primate model for Ebola virus disease
Source: PLoS One. 2021 Jul 2;16(7):e0252874. doi: 10.1371/journal.pone.0252874 (PMC8253449; doi:10.1371/journal.pone.0252874)
Supplement: S29 Table — (DOCX) [file pone.0252874.s029.docx]

### S29 Table. Descriptive Statistics for GGT (U/L) over Time, Overall

| Days Post-Exposure | N | Geometric Mean | Geometric CV(%) | Min | Max | 95% CI |
| --- | --- | --- | --- | --- | --- | --- |
| 0 | 104 | 63 | 51 | 22 | 222 | 57, 69 |
| 1 | 2 | 51 | 20 | 44 | 58 | 9, 292 |
| 3 | 99 | 63 | 48 | 17 | 171 | 57, 68 |
| 4 | 8 | 63 | 71 | 30 | 196 | 37, 107 |
| 5 | 71 | 89 | 66 | 24 | 284 | 77, 102 |
| 6 | 43 | 174 | 76 | 33 | 623 | 142, 215 |
| 7 | 56 | 165 | 101 | 27 | 813 | 132, 207 |
| 8 | 15 | 283 | 49 | 86 | 581 | 218, 366 |
| 9 | 8 | 363 | 32 | 198 | 521 | 279, 472 |
| 10 | 12 | 119 | 128 | 32 | 656 | 63, 221 |
| 11 | 1 | 222 | - - | 222 | 222 | - -, - - |
| 14 | 4 | 64 | 81 | 32 | 165 | 21, 198 |
| 21 | 1 | 49 | - - | 49 | 49 | - -, - - |
| T | 68 | 297 | 47 | 86 | 813 | 267, 331 |

### 
