# Supplementary material for: Natural history of disease in cynomolgus monkeys exposed to Ebola virus Kikwit strain demonstrates the reliability of this non-human primate model for Ebola virus disease
Source: PLoS One. 2021 Jul 2;16(7):e0252874. doi: 10.1371/journal.pone.0252874 (PMC8253449; doi:10.1371/journal.pone.0252874)
Supplement: S30 Table — (DOCX) [file pone.0252874.s030.docx]

### S30 Table. Descriptive Statistics for CRP (mg/L) over Time, Overall

| Days Post-Exposure | N | Geometric Mean | Geometric CV(%) | Min | Max | 95% CI |
| --- | --- | --- | --- | --- | --- | --- |
| 0 | 17 | 7.0 | 27.3 | 5.0 | 12.7 | 6.1, 8 |
| 3 | 21 | 9.0 | 63.6 | 5.1 | 75.9 | 6.9, 11.8 |
| 4 | 2 | 30.2 | 12.9 | 27.6 | 33.1 | 9.5, 95.9 |
| 5 | 16 | 12.8 | 144.3 | 5.1 | 137.7 | 7.3, 22.5 |
| 6 | 12 | 119.1 | 12.8 | 89.2 | 134.1 | 109.8, 129.2 |
| 7 | 25 | 83.0 | 113.4 | 9.5 | 179.0 | 57, 120.8 |
| 8 | 8 | 142.3 | 23.2 | 88.2 | 185.0 | 117.5, 172.2 |
| 9 | 4 | 155.7 | 7.1 | 147.0 | 171.0 | 139.2, 174.2 |
| 10 | 7 | 68.0 | 287 | 7.2 | 177.0 | 17.1, 270.2 |
| 11 | 1 | 141.0 | - - | 141.0 | 141.0 | - -, - - |
| 14 | 2 | 38.5 | 622.7 | 9.9 | 149.4 | 0, 1184383949.7 |
| T | 19 | 139.0 | 30.8 | 51.4 | 185.0 | 120.2, 160.7 |

### 
