# Supplementary material for: Natural history of disease in cynomolgus monkeys exposed to Ebola virus Kikwit strain demonstrates the reliability of this non-human primate model for Ebola virus disease
Source: PLoS One. 2021 Jul 2;16(7):e0252874. doi: 10.1371/journal.pone.0252874 (PMC8253449; doi:10.1371/journal.pone.0252874)
Supplement: S31 Table — (DOCX) [file pone.0252874.s031.docx]

### S31 Table. Descriptive Statistics for Bilirubin (mg/dL) over Time, Overall

| Days Post-Exposure | N | Mean | SD | Min | Max | 95% CI |
| --- | --- | --- | --- | --- | --- | --- |
| 0 | 88 | 0.47 | 0.6 | 0.3 | 6.0 | 0.34, 0.6 |
| 1 | 2 | 0.35 | 0.07 | 0.3 | 0.4 | 0, 0.99 |
| 3 | 86 | 0.41 | 0.09 | 0.3 | 0.7 | 0.39, 0.43 |
| 4 | 2 | 0.40 | - - | 0.4 | 0.4 | - -, - - |
| 5 | 70 | 0.46 | 0.13 | 0.2 | 0.8 | 0.43, 0.49 |
| 6 | 28 | 0.53 | 0.25 | 0.3 | 1.3 | 0.43, 0.63 |
| 7 | 48 | 0.68 | 0.54 | 0.3 | 2.6 | 0.52, 0.84 |
| 8 | 13 | 1.46 | 1.99 | 0.4 | 7.9 | 0.26, 2.67 |
| 9 | 7 | 1.70 | 0.45 | 0.8 | 2.1 | 1.29, 2.11 |
| 10 | 10 | 0.83 | 0.59 | 0.3 | 2.1 | 0.41, 1.25 |
| 11 | 1 | 1.00 | - - | 1.0 | 1.0 | - -, - - |
| 14 | 2 | 0.35 | 0.07 | 0.3 | 0.4 | 0, 0.99 |
| 21 | 1 | 0.30 | - - | 0.3 | 0.3 | - -, - - |
| T | 59 | 1.18 | 1.07 | 0.4 | 7.9 | 0.9, 1.46 |
