# Supplementary material for: Natural history of disease in cynomolgus monkeys exposed to Ebola virus Kikwit strain demonstrates the reliability of this non-human primate model for Ebola virus disease
Source: PLoS One. 2021 Jul 2;16(7):e0252874. doi: 10.1371/journal.pone.0252874 (PMC8253449; doi:10.1371/journal.pone.0252874)
Supplement: S32 Table — (DOCX) [file pone.0252874.s032.docx]

### S32 Table. Descriptive Statistics for BUN (mg/dL) over Time, Overall

| Days Post-Exposure | N | Mean | SD | Min | Max | 95% CI |
| --- | --- | --- | --- | --- | --- | --- |
| 0 | 103 | 14 | 7 | 4 | 62 | 13, 15 |
| 1 | 2 | 14 | 2 | 12 | 15 | 0, 33 |
| 3 | 103 | 14 | 4 | 5 | 25 | 13, 15 |
| 4 | 4 | 15 | 2 | 13 | 18 | 12, 18 |
| 5 | 71 | 21 | 17 | 6 | 100 | 17, 25 |
| 6 | 44 | 53 | 43 | 11 | 169 | 40, 66 |
| 7 | 50 | 58 | 50 | 6 | 171 | 44, 73 |
| 8 | 15 | 117 | 42 | 40 | 200 | 93, 140 |
| 9 | 6 | 142 | 27 | 101 | 170 | 113, 170 |
| 10 | 12 | 53 | 59 | 6 | 161 | 15, 90 |
| 11 | 1 | 97 | - - | 97 | 97 | - -, - - |
| 14 | 4 | 21 | 13 | 13 | 40 | 1, 41 |
| 21 | 1 | 18 | - - | 18 | 18 | - -, - - |
| T | 63 | 105 | 44 | 26 | 200 | 94, 116 |
