# Supplementary material for: Natural history of disease in cynomolgus monkeys exposed to Ebola virus Kikwit strain demonstrates the reliability of this non-human primate model for Ebola virus disease
Source: PLoS One. 2021 Jul 2;16(7):e0252874. doi: 10.1371/journal.pone.0252874 (PMC8253449; doi:10.1371/journal.pone.0252874)
Supplement: S33 Table — (DOCX) [file pone.0252874.s033.docx]

### S33 Table. Descriptive Statistics for Creatinine (mg/dL) over Time, Overall

| Days Post-Exposure | N | Mean | SD | Min | Max | 95% CI |
| --- | --- | --- | --- | --- | --- | --- |
| 0 | 54 | 0.7 | 0.2 | 0.4 | 1.2 | 0.7, 0.8 |
| 1 | 2 | 0.8 | - - | 0.8 | 0.8 | - -, - - |
| 3 | 54 | 0.8 | 0.2 | 0.5 | 1.2 | 0.7, 0.8 |
| 4 | 4 | 0.7 | 0.1 | 0.6 | 0.8 | 0.6, 0.8 |
| 5 | 39 | 1.9 | 1.5 | 0.6 | 6.3 | 1.4, 2.4 |
| 6 | 23 | 3.9 | 3 | 0.8 | 10.7 | 2.6, 5.2 |
| 7 | 22 | 4.3 | 2.4 | 0.6 | 8.6 | 3.3, 5.4 |
| 8 | 5 | 7.9 | 4.3 | 3.4 | 14.3 | 2.6, 13.2 |
| 10 | 5 | 1.0 | 0.4 | 0.6 | 1.4 | 0.5, 1.4 |
| 14 | 2 | 2.7 | 2.5 | 0.9 | 4.5 | 0, 25.6 |
| T | 34 | 5.3 | 2 | 2.2 | 10.7 | 4.6, 6 |
