# Supplementary material for: Natural history of disease in cynomolgus monkeys exposed to Ebola virus Kikwit strain demonstrates the reliability of this non-human primate model for Ebola virus disease
Source: PLoS One. 2021 Jul 2;16(7):e0252874. doi: 10.1371/journal.pone.0252874 (PMC8253449; doi:10.1371/journal.pone.0252874)
Supplement: S34 Table — (DOCX) [file pone.0252874.s034.docx]

### S34 Table. Descriptive Statistics for Calcium (mg/dL) over Time, Overall

| Days Post-Exposure | N | Mean | SD | Min | Max | 95% CI |
| --- | --- | --- | --- | --- | --- | --- |
| 0 | 54 | 9.6 | 0.4 | 9.0 | 10.8 | 9.5, 9.8 |
| 1 | 2 | 9.1 | 0.6 | 8.7 | 9.5 | 4, 14.2 |
| 3 | 54 | 9.6 | 0.6 | 6.7 | 10.7 | 9.4, 9.7 |
| 4 | 4 | 9.2 | 0.4 | 8.7 | 9.5 | 8.7, 9.8 |
| 5 | 37 | 8.6 | 1.1 | 6.1 | 10.1 | 8.2, 9 |
| 6 | 22 | 8.0 | 1.8 | 5.1 | 11.0 | 7.2, 8.8 |
| 7 | 20 | 7.6 | 1.6 | 5.1 | 10.3 | 6.8, 8.4 |
| 8 | 5 | 7.1 | 0.5 | 6.6 | 7.8 | 6.4, 7.7 |
| 10 | 5 | 9.5 | 0.4 | 8.8 | 10.0 | 8.9, 10 |
| 14 | 2 | 8.8 | 1.6 | 7.7 | 10.0 | 0, 23.5 |
| T | 28 | 6.9 | 1.1 | 5.1 | 9.0 | 6.5, 7.3 |
