# Supplementary material for: Natural history of disease in cynomolgus monkeys exposed to Ebola virus Kikwit strain demonstrates the reliability of this non-human primate model for Ebola virus disease
Source: PLoS One. 2021 Jul 2;16(7):e0252874. doi: 10.1371/journal.pone.0252874 (PMC8253449; doi:10.1371/journal.pone.0252874)
Supplement: S35 Table — (DOCX) [file pone.0252874.s035.docx]

### S35 Table. Descriptive Statistics for Albumin (g/dL) over Time, Overall

| Days Post-Exposure | N | Mean | SD | Min | Max | 95% CI |
| --- | --- | --- | --- | --- | --- | --- |
| 0 | 103 | 3.7 | 0.6 | 2.6 | 4.9 | 3.6, 3.8 |
| 1 | 2 | 3.0 | 0.3 | 2.8 | 3.2 | 0.5, 5.5 |
| 3 | 103 | 3.7 | 0.6 | 2.5 | 4.9 | 3.6, 3.8 |
| 4 | 4 | 2.8 | 0.2 | 2.7 | 3.2 | 2.5, 3.2 |
| 5 | 71 | 3.3 | 0.8 | 1.4 | 4.5 | 3.1, 3.5 |
| 6 | 44 | 2.7 | 0.7 | 1.3 | 4.0 | 2.5, 2.9 |
| 7 | 53 | 2.9 | 0.9 | 1.4 | 4.3 | 2.6, 3.1 |
| 8 | 15 | 2.4 | 0.7 | 1.3 | 3.8 | 2, 2.8 |
| 9 | 7 | 2.6 | 0.2 | 2.2 | 2.8 | 2.4, 2.8 |
| 10 | 12 | 3.1 | 0.5 | 2.3 | 4.0 | 2.8, 3.4 |
| 11 | 1 | 2.6 | - - | 2.6 | 2.6 | - -, - - |
| 14 | 3 | 3.4 | 1.2 | 2.0 | 4.2 | 0.4, 6.4 |
| 21 | 1 | 4.0 | - - | 4.0 | 4.0 | - -, - - |
| T | 65 | 2.3 | 0.6 | 1.3 | 3.8 | 2.1, 2.4 |
