# Supplementary material for: Natural history of disease in cynomolgus monkeys exposed to Ebola virus Kikwit strain demonstrates the reliability of this non-human primate model for Ebola virus disease
Source: PLoS One. 2021 Jul 2;16(7):e0252874. doi: 10.1371/journal.pone.0252874 (PMC8253449; doi:10.1371/journal.pone.0252874)
Supplement: S36 Table — (DOCX) [file pone.0252874.s036.docx]

### S36 Table. Descriptive Statistics for Glucose (mg/dL) over Time, Overall

| Days Post-Exposure | N | Geometric Mean | Geometric CV(%) | Min | Max | 95% CI |
| --- | --- | --- | --- | --- | --- | --- |
| 0 | 54 | 64 | 17 | 46 | 105 | 61, 67 |
| 1 | 2 | 66 | 18 | 58 | 75 | 13, 338 |
| 3 | 54 | 68 | 16 | 48 | 105 | 65, 71 |
| 4 | 4 | 76 | 8 | 72 | 86 | 67, 87 |
| 5 | 38 | 62 | 23 | 32 | 79 | 57, 66 |
| 6 | 23 | 61 | 67 | 14 | 194 | 47, 79 |
| 7 | 22 | 54 | 67 | 12 | 117 | 41, 71 |
| 8 | 5 | 39 | 69 | 14 | 66 | 18, 84 |
| 10 | 5 | 59 | 5 | 56 | 64 | 56, 63 |
| 14 | 2 | 86 | 9 | 81 | 92 | 38, 194 |
| T | 33 | 59 | 63 | 17 | 194 | 48, 73 |

### 
