# Supplementary material for: Natural history of disease in cynomolgus monkeys exposed to Ebola virus Kikwit strain demonstrates the reliability of this non-human primate model for Ebola virus disease
Source: PLoS One. 2021 Jul 2;16(7):e0252874. doi: 10.1371/journal.pone.0252874 (PMC8253449; doi:10.1371/journal.pone.0252874)
Supplement: S37 Table — (DOCX) [file pone.0252874.s037.docx]

### S37 Table. Descriptive Statistics for Serum Viral Load by Plaque Assay (PFU/mL) over Time, Overall

| Days Post-Exposure | N | Geometric Mean | Geometric CV(%) | Min | Max | 95% CI |
| --- | --- | --- | --- | --- | --- | --- |
| 0 | 45 | 3.13e-02 | 1.45e+01 | 0e+00 | 1e+00 | 0.00e+00, 8.00e-02 |
| 3 | 38 | 3.12e-01 | 2.68e+02 | 0e+00 | 7.53e+03 | 0.00e+00, 1.11e+00 |
| 4 | 6 | 3.14e+02 | 9.58e+10 | 0e+00 | 5e+05 | 0.00e+00, 2.69e+05 |
| 5 | 23 | 6.13e+02 | 1.48e+09 | 0e+00 | 4.38e+07 | 5.01e+01, 7.36e+03 |
| 6 | 16 | 2.29e+06 | 1.21e+06 | 0e+00 | 2.44e+08 | 2.27e+05, 2.31e+07 |
| 7 | 29 | 8.77e+05 | 7.47e+06 | 0e+00 | 2.99e+08 | 1.45e+05, 5.32e+06 |
| 8 | 7 | 7.31e+06 | 1.19e+03 | 2.08e+05 | 9.25e+07 | 9.30e+05, 5.74e+07 |
| 9 | 6 | 5.51e+06 | 1.47e+04 | 1.25e+04 | 5.38e+07 | 2.00e+05, 1.52e+08 |
| 10 | 7 | 5.19e+04 | 9.08e+14 | 0e+00 | 1.89e+08 | 4.00e+01, 6.57e+07 |
| 11 | 1 | 1.13e+06 | - - | 1.13e+06 | 1.13e+06 | - -, - - |
| 14 | 2 | 0e+00 | - - | 0e+00 | 0e+00 | - -, - - |
| 21 | 1 | 0e+00 | - - | 0e+00 | 0e+00 | - -, - - |
| T | 26 | 6.58e+06 | 3.01e+03 | 1.25e+04 | 2.31e+08 | 2.29e+06, 1.89e+07 |

### 
