# Supplementary material for: Natural history of disease in cynomolgus monkeys exposed to Ebola virus Kikwit strain demonstrates the reliability of this non-human primate model for Ebola virus disease
Source: PLoS One. 2021 Jul 2;16(7):e0252874. doi: 10.1371/journal.pone.0252874 (PMC8253449; doi:10.1371/journal.pone.0252874)
Supplement: S38 Table — (DOCX) [file pone.0252874.s038.docx]

### S38 Table. Descriptive Statistics for Serum Viral Load by Plaque Assay (PFU/mL) over Time, by Exposure Dose (PFU)

| Challenge Dose (PFU) | Days Post-Exposure | N | Geometric Mean | Geometric CV(%) | Min | Max | 95% CI |
| --- | --- | --- | --- | --- | --- | --- | --- |
| (0.1-0.5) | 0 | 20 | 0e+00 | - - | 0e+00 | 0e+00 | - -, - - |
| (0.1-0.5) | 3 | 20 | 0e+00 | - - | 0e+00 | 0e+00 | - -, - - |
| (0.1-0.5) | 5 | 18 | 6.4e+01 | 2.11e+05 | 0e+00 | 6e+05 | 8.29e+00, 4.54e+02 |
| (0.1-0.5) | 7 | 19 | 5.37e+05 | 3.42e+08 | 0e+00 | 2.99e+08 | 3.82e+04, 7.56e+06 |
| (0.1-0.5) | 8 | 5 | 2.26e+07 | 1.98e+02 | 2.95e+06 | 9.25e+07 | 4.73e+06, 1.08e+08 |
| (0.1-0.5) | 9 | 4 | 2.91e+07 | 7.85e+01 | 1.3e+07 | 5.38e+07 | 9.68e+06, 8.77e+07 |
| (0.1-0.5) | 10 | 7 | 5.19e+04 | 9.08e+14 | 0e+00 | 1.89e+08 | 4.00e+01, 6.57e+07 |
| (0.1-0.5) | 11 | 1 | 1.13e+06 | - - | 1.13e+06 | 1.13e+06 | - -, - - |
| (0.1-0.5) | 14 | 2 | 0e+00 | - - | 0e+00 | 0e+00 | - -, - - |
| (0.1-0.5) | 21 | 1 | 0e+00 | - - | 0e+00 | 0e+00 | - -, - - |
| (0.1-0.5) | T | 14 | 2.13e+07 | 4.03e+02 | 9.38e+05 | 2.31e+08 | 8.03e+06, 5.64e+07 |
| (25-243) | 0 | 23 | 6.21e-02 | 2.02e+01 | 0e+00 | 1e+00 | 0.00e+00, 1.60e-01 |
| (25-243) | 3 | 15 | 9.68e-02 | 2.48e+01 | 0e+00 | 1e+00 | 0.00e+00, 2.60e-01 |
| (25-243) | 4 | 4 | 1.99e+01 | 1.06e+10 | 0e+00 | 1.91e+05 | 0.00e+00, 3.33e+05 |
| (25-243) | 5 | 2 | 6.37e+04 | 4.13e+03 | 9.25e+03 | 4.38e+05 | 0.00e+00, 2.80e+15 |
| (25-243) | 6 | 14 | 2.28e+06 | 3.62e+06 | 0e+00 | 2.44e+08 | 1.62e+05, 3.21e+07 |
| (25-243) | 7 | 7 | 4.18e+06 | 4.41e+03 | 2.88e+04 | 1.16e+08 | 3.28e+05, 5.33e+07 |
| (25-243) | 8 | 2 | 4.33e+05 | 1.39e+02 | 2.08e+05 | 9e+05 | 3.83e+01, 4.76e+09 |
| (25-243) | 9 | 2 | 1.97e+05 | 2e+05 | 1.25e+04 | 3.1e+06 | 0.00e+00, 3.21e+20 |
| (25-243) | T | 8 | 1.91e+06 | 8.43e+03 | 1.25e+04 | 1.16e+08 | 1.58e+05, 2.30e+07 |
| (320-1650) | 0 | 2 | 0e+00 | - - | 0e+00 | 0e+00 | - -, - - |
| (320-1650) | 3 | 3 | 1.86e+01 | 5.86e+07 | 0e+00 | 7.53e+03 | 0.00e+00, 7.12e+06 |
| (320-1650) | 4 | 2 | 7.18e+04 | 4.33e+03 | 1.03e+04 | 5e+05 | 0.00e+00, 3.70e+15 |
| (320-1650) | 5 | 3 | 1.97e+07 | 1.24e+02 | 6.75e+06 | 4.38e+07 | 1.80e+06, 2.16e+08 |
| (320-1650) | 6 | 2 | 2.36e+06 | 9.34e+03 | 2.81e+05 | 1.99e+07 | 0.00e+00, 1.34e+18 |
| (320-1650) | 7 | 3 | 5.13e+05 | 1.11e+04 | 1.75e+04 | 7e+06 | 2.50e+02, 1.05e+09 |
| (320-1650) | T | 4 | 1.28e+06 | 1.23e+04 | 1.75e+04 | 1.99e+07 | 9.19e+03, 1.78e+08 |
