# Supplementary material for: Natural history of disease in cynomolgus monkeys exposed to Ebola virus Kikwit strain demonstrates the reliability of this non-human primate model for Ebola virus disease
Source: PLoS One. 2021 Jul 2;16(7):e0252874. doi: 10.1371/journal.pone.0252874 (PMC8253449; doi:10.1371/journal.pone.0252874)
Supplement: S39 Table — (DOCX) [file pone.0252874.s039.docx]

### S39 Table. Descriptive Statistics for Serum Viral Load by qRT-PCR (GE/mL) over Time, Overall

| Days Post-Exposure | N | Geometric Mean | Geometric CV(%) | Min | Max | 95% CI |
| --- | --- | --- | --- | --- | --- | --- |
| 0 | 33 | 7.02e-01 | 4.04e+02 | 0e+00 | 3.99e+03 | 0.00e+00, 2.10e+00 |
| 3 | 34 | 1.92e+01 | 3.36e+08 | 0e+00 | 1.01e+08 | 1.98e+00, 1.36e+02 |
| 4 | 5 | 4.5e+05 | 2.02e+08 | 7.69e+03 | 2.85e+09 | 5.58e+02, 3.63e+08 |
| 5 | 28 | 4.25e+03 | 3.08e+23 | 0e+00 | 6.84e+11 | 8.88e+01, 2.01e+05 |
| 6 | 15 | 1.01e+08 | 2.25e+03 | 9.51e+05 | 7.51e+09 | 2.54e+07, 4.04e+08 |
| 7 | 31 | 1.48e+07 | 6.95e+10 | 0e+00 | 3.21e+10 | 1.43e+06, 1.54e+08 |
| 8 | 5 | 4.62e+08 | 2.3e+02 | 7.77e+07 | 2.43e+09 | 8.56e+07, 2.49e+09 |
| 9 | 5 | 2.19e+08 | 3.7e+02 | 1.7e+07 | 1.35e+09 | 2.85e+07, 1.67e+09 |
| 10 | 7 | 3.43e+05 | 3.37e+21 | 0e+00 | 4.46e+09 | 5.22e+01, 2.21e+09 |
| 11 | 1 | 1.68e+07 | - - | 1.68e+07 | 1.68e+07 | - -, - - |
| 14 | 2 | 0e+00 | - - | 0e+00 | 0e+00 | - -, - - |
| 21 | 1 | 0e+00 | - - | 0e+00 | 0e+00 | - -, - - |
| T | 27 | 2.06e+08 | 3.64e+04 | 6.99e+04 | 3.21e+10 | 5.30e+07, 8.02e+08 |

### 
