# Supplementary material for: Natural history of disease in cynomolgus monkeys exposed to Ebola virus Kikwit strain demonstrates the reliability of this non-human primate model for Ebola virus disease
Source: PLoS One. 2021 Jul 2;16(7):e0252874. doi: 10.1371/journal.pone.0252874 (PMC8253449; doi:10.1371/journal.pone.0252874)
Supplement: S40 Table — (DOCX) [file pone.0252874.s040.docx]

### S40 Table. Descriptive Statistics for Serum Viral Load by qRT-PCR (GE/mL) over Time, by Exposure Dose (PFU)

| Challenge Dose (PFU) | Days Post-Exposure | N | Geometric Mean | Geometric CV(%) | Min | Max | 95% CI |
| --- | --- | --- | --- | --- | --- | --- | --- |
| (0.1-0.5) | 0 | 20 | 0e+00 | - - | 0e+00 | 0e+00 | - -, - - |
| (0.1-0.5) | 3 | 20 | 0e+00 | - - | 0e+00 | 0e+00 | - -, - - |
| (0.1-0.5) | 5 | 20 | 1.59e+01 | 3.11e+05 | 0e+00 | 3.62e+04 | 1.59e+00, 1.10e+02 |
| (0.1-0.5) | 7 | 20 | 5e+06 | 3.24e+13 | 0e+00 | 1.62e+10 | 1.66e+05, 1.51e+08 |
| (0.1-0.5) | 8 | 5 | 4.62e+08 | 2.3e+02 | 7.77e+07 | 2.43e+09 | 8.56e+07, 2.49e+09 |
| (0.1-0.5) | 9 | 4 | 4.14e+08 | 1.18e+02 | 1.45e+08 | 1.35e+09 | 9.40e+07, 1.82e+09 |
| (0.1-0.5) | 10 | 7 | 3.43e+05 | 3.37e+21 | 0e+00 | 4.46e+09 | 5.22e+01, 2.21e+09 |
| (0.1-0.5) | 11 | 1 | 1.68e+07 | - - | 1.68e+07 | 1.68e+07 | - -, - - |
| (0.1-0.5) | 14 | 2 | 0e+00 | - - | 0e+00 | 0e+00 | - -, - - |
| (0.1-0.5) | 21 | 1 | 0e+00 | - - | 0e+00 | 0e+00 | - -, - - |
| (0.1-0.5) | T | 14 | 3.3e+08 | 9.57e+03 | 6.99e+04 | 1.62e+10 | 5.76e+07, 1.89e+09 |
| (25-243) | 0 | 13 | 2.86e+00 | 2.47e+03 | 0e+00 | 3.99e+03 | 0.00e+00, 1.68e+01 |
| (25-243) | 3 | 10 | 5.53e+01 | 5.16e+05 | 0e+00 | 5.4e+03 | 1.92e+00, 1.08e+03 |
| (25-243) | 4 | 4 | 5.05e+04 | 2.99e+03 | 7.69e+03 | 2.01e+06 | 7.95e+02, 3.20e+06 |
| (25-243) | 5 | 4 | 7.21e+07 | 5.86e+08 | 2.06e+05 | 1.8e+10 | 1.00e+04, 5.20e+11 |
| (25-243) | 6 | 15 | 1.01e+08 | 2.25e+03 | 9.51e+05 | 7.51e+09 | 2.54e+07, 4.04e+08 |
| (25-243) | 7 | 9 | 5.13e+07 | 9.27e+04 | 1.41e+05 | 3.46e+09 | 2.99e+06, 8.80e+08 |
| (25-243) | 9 | 1 | 1.7e+07 | - - | 1.7e+07 | 1.7e+07 | - -, - - |
| (25-243) | T | 11 | 6.99e+07 | 1.52e+05 | 1.41e+05 | 1.8e+10 | 5.34e+06, 9.14e+08 |
| (320-1650) | 3 | 4 | 5.27e+06 | 5.98e+03 | 1.12e+05 | 1.01e+08 | 5.56e+04, 5.00e+08 |
| (320-1650) | 4 | 1 | 2.85e+09 | - - | 2.85e+09 | 2.85e+09 | - -, - - |
| (320-1650) | 5 | 4 | 2.51e+11 | 9.54e+01 | 9.93e+10 | 6.84e+11 | 6.97e+10, 9.03e+11 |
| (320-1650) | 7 | 2 | 2.97e+09 | 2.88e+04 | 2.75e+08 | 3.21e+10 | 0.00e+00, 4.04e+22 |
| (320-1650) | T | 2 | 2.97e+09 | 2.88e+04 | 2.75e+08 | 3.21e+10 | 0.00e+00, 4.04e+22 |
