# Supplementary material for: Natural history of disease in cynomolgus monkeys exposed to Ebola virus Kikwit strain demonstrates the reliability of this non-human primate model for Ebola virus disease
Source: PLoS One. 2021 Jul 2;16(7):e0252874. doi: 10.1371/journal.pone.0252874 (PMC8253449; doi:10.1371/journal.pone.0252874)
Supplement: S41 Table — (DOCX) [file pone.0252874.s041.docx]

### S41 Table. Descriptive Statistics for Tissue Viral Load by Plaque Assay (PFU/g), Overall.

| Parameter Name | N | Geometric Mean | Geometric CV(%) | Min | Max | 95% CI |
| --- | --- | --- | --- | --- | --- | --- |
| Lung Plaque Assay | 37 | 1.1e+06 | 2.31e+09 | 0e+00 | 3.81e+09 | 1.57e+05, 7.64e+06 |
| Liver Plaque Assay | 35 | 2.27e+07 | 3.02e+05 | 0e+00 | 5.7e+09 | 5.73e+06, 8.96e+07 |
| Adrenal Gland Plaque Assay | 34 | 1.08e+07 | 7.42e+06 | 0e+00 | 2.67e+10 | 2.06e+06, 5.62e+07 |
| Kidney Plaque Assay | 23 | 3.27e+06 | 7.61e+04 | 0e+00 | 8.78e+07 | 6.77e+05, 1.58e+07 |
| Inguinal Lymph Node Plaque Assay | 21 | 4.92e+06 | 1.93e+05 | 0e+00 | 1.67e+08 | 8.38e+05, 2.89e+07 |
| Hilar Lymph Node Plaque Assay | 21 | 5.49e+06 | 8.36e+04 | 0e+00 | 1.08e+08 | 1.03e+06, 2.91e+07 |
| Axillary Lymph Node Plaque Assay | 17 | 7.11e+07 | 6.92e+03 | 7.93e+04 | 1.19e+10 | 1.59e+07, 3.18e+08 |
| Spleen Plaque Assay | 14 | 1.76e+08 | 2.7e+04 | 4.38e+04 | 9.92e+10 | 2.55e+07, 1.21e+09 |

### 
