# Supplementary material for: Natural history of disease in cynomolgus monkeys exposed to Ebola virus Kikwit strain demonstrates the reliability of this non-human primate model for Ebola virus disease
Source: PLoS One. 2021 Jul 2;16(7):e0252874. doi: 10.1371/journal.pone.0252874 (PMC8253449; doi:10.1371/journal.pone.0252874)
Supplement: S42 Table — (DOCX) [file pone.0252874.s042.docx]

### S42 Table. Descriptive Statistics for Tissue Viral Load by Plaque Assay (PFU/g), by Sex

| Sex | Parameter Name | N | Geometric Mean | Geometric CV(%) | Min | Max | 95% CI |
| --- | --- | --- | --- | --- | --- | --- | --- |
| Female | Lung Plaque Assay | 19 | 2.32e+06 | 2.11e+08 | 0e+00 | 7.25e+08 | 1.72e+05, 3.13e+07 |
| Female | Liver Plaque Assay | 19 | 6.54e+07 | 1.07e+03 | 9.71e+04 | 5.7e+09 | 2.29e+07, 1.87e+08 |
| Female | Adrenal Gland Plaque Assay | 19 | 3.08e+07 | 1.04e+03 | 1.09e+06 | 2.67e+10 | 1.08e+07, 8.74e+07 |
| Female | Kidney Plaque Assay | 12 | 8.35e+06 | 3.91e+02 | 4.79e+05 | 8.78e+07 | 2.89e+06, 2.41e+07 |
| Female | Inguinal Lymph Node Plaque Assay | 12 | 1.29e+07 | 2.82e+02 | 1.49e+06 | 1.42e+08 | 5.03e+06, 3.30e+07 |
| Female | Hilar Lymph Node Plaque Assay | 12 | 1.21e+07 | 1.16e+02 | 3.06e+06 | 3.98e+07 | 6.73e+06, 2.18e+07 |
| Female | Axillary Lymph Node Plaque Assay | 7 | 5.97e+07 | 7.79e+02 | 2.11e+06 | 3.46e+08 | 9.13e+06, 3.90e+08 |
| Female | Spleen Plaque Assay | 7 | 3.98e+08 | 1.33e+04 | 3.31e+06 | 9.92e+10 | 2.21e+07, 7.17e+09 |
| Male | Lung Plaque Assay | 18 | 4.96e+05 | 4.12e+10 | 0e+00 | 3.81e+09 | 2.16e+04, 1.14e+07 |
| Male | Liver Plaque Assay | 16 | 6.43e+06 | 9.39e+07 | 0e+00 | 4.55e+09 | 3.93e+05, 1.05e+08 |
| Male | Adrenal Gland Plaque Assay | 15 | 2.85e+06 | 2.72e+11 | 0e+00 | 3.24e+09 | 7.40e+04, 1.10e+08 |
| Male | Kidney Plaque Assay | 11 | 1.18e+06 | 1.56e+07 | 0e+00 | 3.26e+07 | 4.40e+04, 3.14e+07 |
| Male | Inguinal Lymph Node Plaque Assay | 9 | 1.36e+06 | 7.14e+08 | 0e+00 | 1.67e+08 | 1.81e+04, 1.02e+08 |
| Male | Hilar Lymph Node Plaque Assay | 9 | 1.91e+06 | 3.75e+08 | 0e+00 | 1.08e+08 | 2.78e+04, 1.31e+08 |
| Male | Axillary Lymph Node Plaque Assay | 10 | 8.03e+07 | 4.63e+04 | 7.93e+04 | 1.19e+10 | 6.55e+06, 9.85e+08 |
| Male | Spleen Plaque Assay | 7 | 7.79e+07 | 6.43e+04 | 4.38e+04 | 2.5e+09 | 2.80e+06, 2.17e+09 |
