# Supplementary material for: Natural history of disease in cynomolgus monkeys exposed to Ebola virus Kikwit strain demonstrates the reliability of this non-human primate model for Ebola virus disease
Source: PLoS One. 2021 Jul 2;16(7):e0252874. doi: 10.1371/journal.pone.0252874 (PMC8253449; doi:10.1371/journal.pone.0252874)
Supplement: S43 Table — (DOCX) [file pone.0252874.s043.docx]

### S43 Table. Descriptive Statistics for Tissue Viral Load by Plaque Assay (PFU/g), by Animal Origin

| Animal Origin | Parameter Name | N | Geometric Mean | Geometric CV(%) | Min | Max | 95% CI |
| --- | --- | --- | --- | --- | --- | --- | --- |
| Asian | Lung Plaque Assay | 28 | 3.6e+05 | 2.03e+10 | 0e+00 | 7.84e+07 | 3.27e+04, 3.96e+06 |
| Asian | Liver Plaque Assay | 26 | 9.95e+06 | 7.35e+05 | 0e+00 | 5.02e+08 | 1.81e+06, 5.47e+07 |
| Asian | Adrenal Gland Plaque Assay | 25 | 3.28e+06 | 6.62e+06 | 0e+00 | 8.48e+07 | 4.68e+05, 2.29e+07 |
| Asian | Kidney Plaque Assay | 23 | 3.27e+06 | 7.61e+04 | 0e+00 | 8.78e+07 | 6.77e+05, 1.58e+07 |
| Asian | Inguinal Lymph Node Plaque Assay | 21 | 4.92e+06 | 1.93e+05 | 0e+00 | 1.67e+08 | 8.38e+05, 2.89e+07 |
| Asian | Hilar Lymph Node Plaque Assay | 21 | 5.49e+06 | 8.36e+04 | 0e+00 | 1.08e+08 | 1.03e+06, 2.91e+07 |
| Asian | Axillary Lymph Node Plaque Assay | 8 | 1.7e+07 | 3.53e+03 | 7.93e+04 | 2.03e+08 | 1.83e+06, 1.59e+08 |
| Asian | Spleen Plaque Assay | 5 | 1.34e+07 | 9.34e+04 | 4.38e+04 | 4.6e+08 | 1.36e+05, 1.33e+09 |
| Mauritian | Lung Plaque Assay | 9 | 3.5e+07 | 1.69e+03 | 2.75e+06 | 3.81e+09 | 5.62e+06, 2.18e+08 |
| Mauritian | Liver Plaque Assay | 9 | 2.44e+08 | 6.99e+02 | 3.9e+07 | 5.7e+09 | 5.35e+07, 1.12e+09 |
| Mauritian | Adrenal Gland Plaque Assay | 9 | 2.94e+08 | 9.94e+03 | 3.06e+06 | 2.67e+10 | 2.85e+07, 3.02e+09 |
| Mauritian | Axillary Lymph Node Plaque Assay | 9 | 2.53e+08 | 3.08e+03 | 2.55e+06 | 1.19e+10 | 3.38e+07, 1.89e+09 |
| Mauritian | Spleen Plaque Assay | 9 | 7.35e+08 | 1.17e+03 | 4.64e+07 | 9.92e+10 | 1.34e+08, 4.04e+09 |
