# Supplementary material for: Natural history of disease in cynomolgus monkeys exposed to Ebola virus Kikwit strain demonstrates the reliability of this non-human primate model for Ebola virus disease
Source: PLoS One. 2021 Jul 2;16(7):e0252874. doi: 10.1371/journal.pone.0252874 (PMC8253449; doi:10.1371/journal.pone.0252874)
Supplement: S44 Table — (DOCX) [file pone.0252874.s044.docx]

### S44 Table. Descriptive Statistics for Tissue Viral Load by qRT-PCR (GE/µg), Overall

| Parameter Name | N | Geometric Mean | Geometric CV(%) | Min | Max | 95% CI |
| --- | --- | --- | --- | --- | --- | --- |
| Lung qRT-PCR | 29 | 1.12e+04 | 4.39e+07 | 0e+00 | 4.98e+06 | 1.62e+03, 7.81e+04 |
| Liver qRT-PCR | 30 | 4.58e+06 | 1.92e+04 | 6.23e+02 | 1.7e+08 | 1.36e+06, 1.54e+07 |
| Adrenal Gland qRT-PCR | 31 | 1.86e+06 | 6.75e+03 | 2.08e+02 | 7.5e+07 | 6.42e+05, 5.40e+06 |
| Kidney qRT-PCR | 23 | 5.19e+05 | 2.73e+04 | 1.69e+01 | 7.66e+06 | 1.22e+05, 2.21e+06 |
| Inguinal Lymph Node qRT-PCR | 22 | 1.47e+06 | 3.95e+03 | 4.75e+02 | 7.92e+07 | 4.43e+05, 4.91e+06 |
| Hilar Lymph Node qRT-PCR | 20 | 6.68e+06 | 2.06e+02 | 6.7e+05 | 4.25e+07 | 3.66e+06, 1.22e+07 |

### 
