# Supplementary material for: Natural history of disease in cynomolgus monkeys exposed to Ebola virus Kikwit strain demonstrates the reliability of this non-human primate model for Ebola virus disease
Source: PLoS One. 2021 Jul 2;16(7):e0252874. doi: 10.1371/journal.pone.0252874 (PMC8253449; doi:10.1371/journal.pone.0252874)
Supplement: S45 Table — (DOCX) [file pone.0252874.s045.docx]

### S45 Table. Descriptive Statistics for Tissue Viral Load by qRT-PCR (GE/µg), by Sex

| Sex | Parameter Name | N | Geometric Mean | Geometric CV(%) | Min | Max | 95% CI |
| --- | --- | --- | --- | --- | --- | --- | --- |
| Female | Lung qRT-PCR | 16 | 6.16e+04 | 4.93e+05 | 0e+00 | 4.98e+06 | 6.84e+03, 5.55e+05 |
| Female | Liver qRT-PCR | 16 | 1.72e+07 | 3.22e+02 | 1.51e+06 | 1.7e+08 | 7.48e+06, 3.94e+07 |
| Female | Adrenal Gland qRT-PCR | 16 | 5.58e+06 | 3.62e+02 | 6.53e+05 | 7.5e+07 | 2.34e+06, 1.33e+07 |
| Female | Kidney qRT-PCR | 12 | 1.41e+06 | 1.6e+02 | 1.36e+05 | 7.66e+06 | 6.86e+05, 2.88e+06 |
| Female | Inguinal Lymph Node qRT-PCR | 12 | 3.14e+06 | 3.58e+02 | 9.93e+04 | 7.92e+07 | 1.12e+06, 8.78e+06 |
| Female | Hilar Lymph Node qRT-PCR | 12 | 8.48e+06 | 1.49e+02 | 1.64e+06 | 4.25e+07 | 4.26e+06, 1.69e+07 |
| Male | Lung qRT-PCR | 13 | 1.38e+03 | 4.78e+08 | 0e+00 | 1.01e+06 | 4.84e+01, 3.95e+04 |
| Male | Liver qRT-PCR | 14 | 1.01e+06 | 3.05e+05 | 6.23e+02 | 1.11e+08 | 9.99e+04, 1.02e+07 |
| Male | Adrenal Gland qRT-PCR | 15 | 5.77e+05 | 4.86e+04 | 2.08e+02 | 2.39e+07 | 8.23e+04, 4.05e+06 |
| Male | Kidney qRT-PCR | 11 | 1.75e+05 | 3.28e+06 | 1.69e+01 | 6.75e+06 | 8.18e+03, 3.75e+06 |
| Male | Inguinal Lymph Node qRT-PCR | 10 | 5.96e+05 | 4.64e+04 | 4.75e+02 | 5.91e+07 | 4.86e+04, 7.31e+06 |
| Male | Hilar Lymph Node qRT-PCR | 8 | 4.68e+06 | 3.21e+02 | 6.7e+05 | 4.13e+07 | 1.27e+06, 1.72e+07 |

### 
