# Supplementary material for: Natural history of disease in cynomolgus monkeys exposed to Ebola virus Kikwit strain demonstrates the reliability of this non-human primate model for Ebola virus disease
Source: PLoS One. 2021 Jul 2;16(7):e0252874. doi: 10.1371/journal.pone.0252874 (PMC8253449; doi:10.1371/journal.pone.0252874)
Supplement: S46 Table — (DOCX) [file pone.0252874.s046.docx]

### S46 Table. Descriptive Statistics for Tissue Viral Load by qRT-PCR (GE/µg), by Animal Origin

| Animal Origin | Parameter Name | N | Geometric Mean | Geometric CV(%) | Min | Max | 95% CI |
| --- | --- | --- | --- | --- | --- | --- | --- |
| Asian | Lung qRT-PCR | 22 | 8.62e+03 | 7.69e+08 | 0e+00 | 4.98e+06 | 7.10e+02, 1.05e+05 |
| Asian | Liver qRT-PCR | 23 | 4.54e+06 | 7.57e+04 | 6.23e+02 | 1.7e+08 | 9.40e+05, 2.19e+07 |
| Asian | Adrenal Gland qRT-PCR | 23 | 2.04e+06 | 1.85e+04 | 2.08e+02 | 7.5e+07 | 5.04e+05, 8.25e+06 |
| Asian | Kidney qRT-PCR | 23 | 5.19e+05 | 2.73e+04 | 1.69e+01 | 7.66e+06 | 1.22e+05, 2.21e+06 |
| Asian | Inguinal Lymph Node qRT-PCR | 22 | 1.47e+06 | 3.95e+03 | 4.75e+02 | 7.92e+07 | 4.43e+05, 4.91e+06 |
| Asian | Hilar Lymph Node qRT-PCR | 20 | 6.68e+06 | 2.06e+02 | 6.7e+05 | 4.25e+07 | 3.66e+06, 1.22e+07 |
| Mauritian | Lung qRT-PCR | 7 | 2.58e+04 | 1e+04 | 7.55e+01 | 7.36e+05 | 1.56e+03, 4.28e+05 |
| Mauritian | Liver qRT-PCR | 7 | 4.7e+06 | 2.87e+02 | 1.51e+06 | 7.32e+07 | 1.19e+06, 1.87e+07 |
| Mauritian | Adrenal Gland qRT-PCR | 8 | 1.43e+06 | 4.85e+02 | 1.42e+05 | 6.61e+07 | 3.20e+05, 6.38e+06 |
